# Supplementary material for: Secondary influenza challenge triggers resident memory B cell migration and rapid relocation to boost antibody secretion at infected sites
Source: Immunity. 2022 Apr 12;55(4):718–733.e8. doi: 10.1016/j.immuni.2022.03.003 (PMC9044924; doi:10.1016/j.immuni.2022.03.003)
Supplement: Document S1. Figures S1–S7 [file mmc1.pdf]

Immunity, Volume *55*

Supplemental information

Secondary influenza challenge triggers resident memory B cell migration and rapid relocation to boost antibody secretion at infected sites

Andrew J. MacLean, Niamh Richmond, Lada Koneva, Moustafa Attar, Cesar A.P. Medina, Emily E. Thornton, Ariane Cruz Gomes, Aadil El-Turabi, Martin F. Bachmann, Pramila Rijal, Tiong Kit Tan, Alain Townsend, Stephen N. Sansom, Oliver Bannard, and Tal I. Arnon

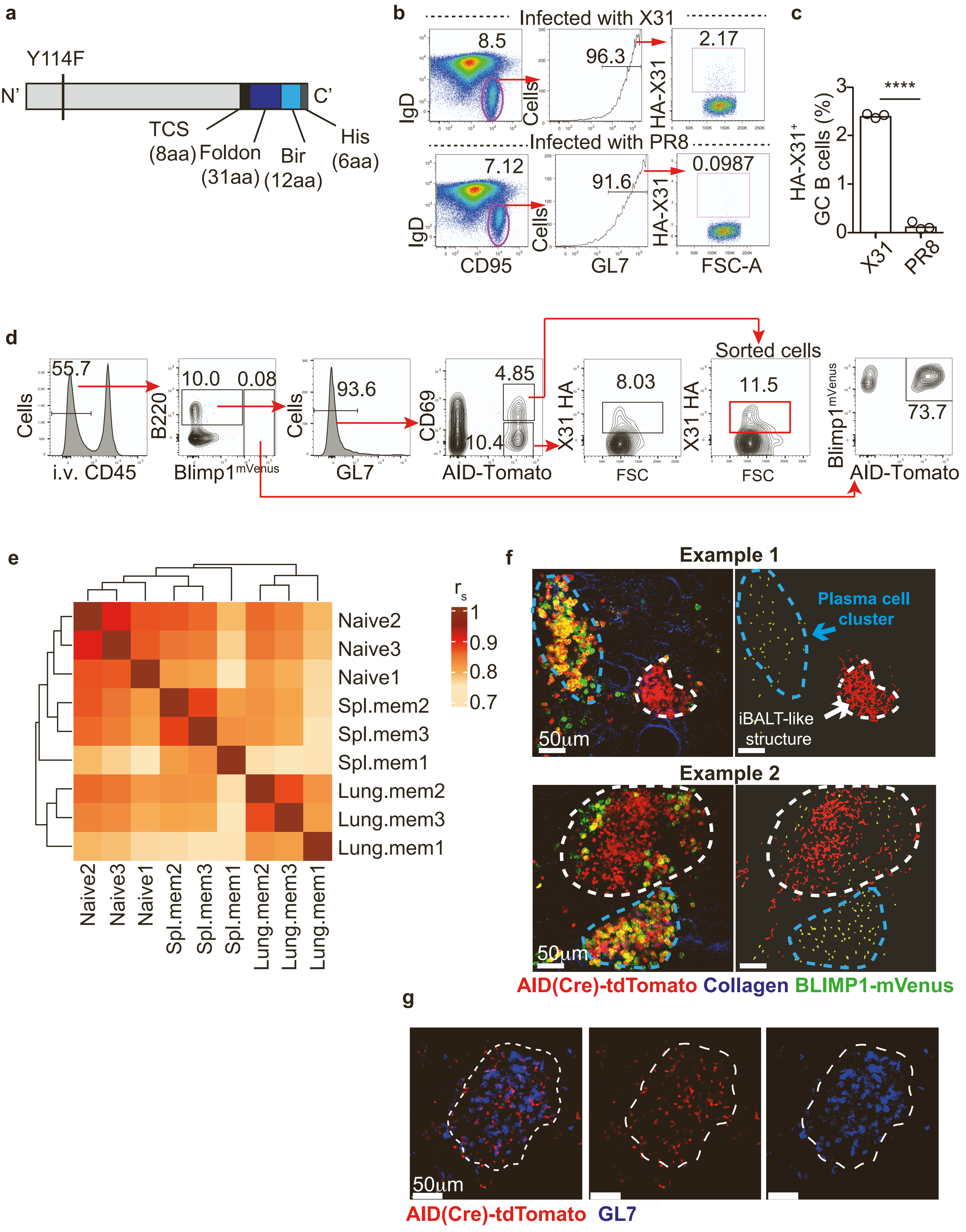

a
Y114F
N'
C'
TCS
(8aa)
Foldon
(31aa)
Bir
(12aa)
His
(6aa)
b
Infected with X31
Infected with PR8
8.5
96.3
2.17
7.12
91.6
0.0987
IgD
Cells
HA-X31
CD95
GL7
FSC-A
c
****
HA-X31+ GC B cells (%)
X31
PR8
d
55.7
10.0
0.08
93.6
4.85
10.4
8.03
11.5
73.7
Sorted cells
Cells
B220
CD69
X31 HA
Blimp1mVenus
i.v. CD45
GL7
AID-Tomato
FSC
e
rs
1
0.9
0.8
0.7
Naive2
Naive3
Naive1
Spl.mem2
Spl.mem3
Spl.mem1
Lung.mem2
Lung.mem3
Lung.mem1
f
Example 1
Example 2
Plasma cell cluster
iBALT-like structure
50μm
AID(Cre)-tdTomato Collagen BLIMP1-mVenus
g
50μm
AID(Cre)-tdTomato GL7

Supplementary Fig. 1 Detection of germinal centre B cells in influenza infected mice, related to Fig.1
(a-c) Detection of X31 hemagglutinin (X31-HA) specific B cells. (a) Construct design for HA probe. Y114F mutation (equivalent to Y98F in other strains) to reduce sialic acid binding, thrombin cleavage site (TCS), Foldon domain, BirA biotinylating site, and 6xHis-tag tag to allow purification. (b) Representative FACS plots displaying HA staining (Biotinylated) of GC B cells from mediastinal LN of X31 and PR8-infected mice at day 8 post infection, (c) enumerated for multiple mice. Each circle represents one mouse. (d) FACS gating strategy for sorting lung $CD69^{+}$ HA^{+} BRM cells for RNA-seq analysis and to quantify lung PCs and BRM cells. Figure shows representative FACS plots of lungs from infected BAT chimeras > 6 weeks post infection with X31 influenza. Mice were in vivo labelled with anti-CD45 (i.v. CD45) 4 mins before tissue collection. Plots are pre-gated on live cells. The gate used for sorting of HA^{+} $CD69^{+}$ BRM cells is highlighted in red. (e) Dendrogram and symmetrical heatmap visualisation of bulk RNA-seq correlation matrix showing inter-sample Spearman coefficients (r_s) between naïve, and memory B cells derived from the spleen (Spl.mem) and lung (Lung.mem). Scale bar represents the range of the correlation coefficients displayed. (f) Snapshots from videos of live explant BAT mice during the memory phase (42 days post primary infection) acquired with TPLSM. White dotted line outlines iBALT-like structures. Blue dotted line delineates cluster of PCs. Example 1 shows an iBALT-like structure containing $tdTomato^{+}mVenus^{-}$ cells with no PCs. Example 2 shows an iBALT-like structure containing $tdTomato^{+}mVenus^{-}$ cells surrounded by PCs. In both examples, a PC cluster containing primarily $tdTomato^{+}mVenus^{+}$ PCs is present adjacent to the iBALT-like structure. In each example, snapshots of cell distribution (left) and tracks of migrating cells (right) are shown. (g) Confocal microscopy of lungs from BAT mice stained with anti-GL7 (blue) 42 days post primary infection.

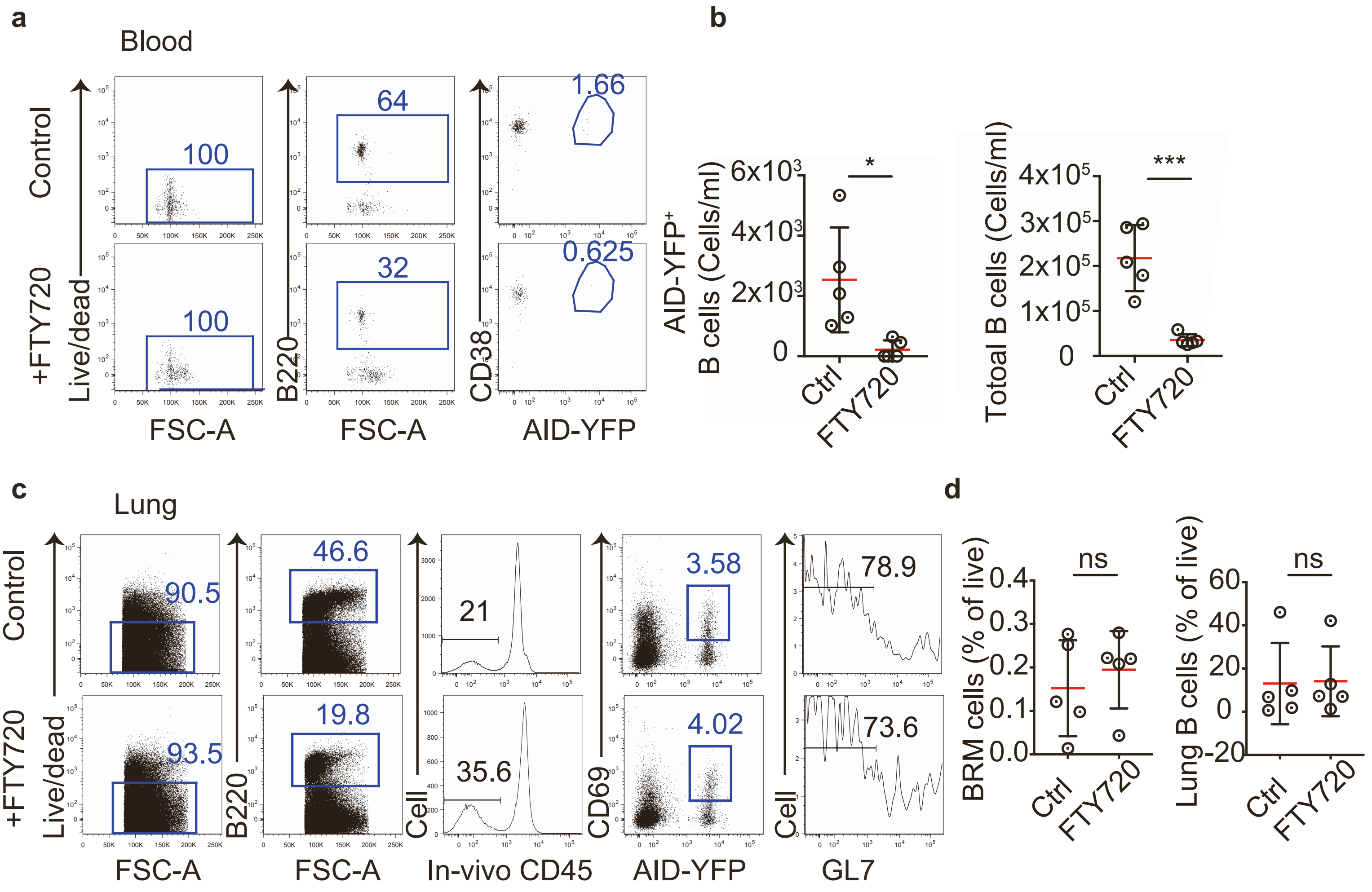

Supplementary Fig. 2. FTY720 treatment sequesters memory B cells in SLOs and removes them from the circulation, related to Fig.2

AID$^{Cre/+}$ Rosa26$^{-stop-EYFP}$ mice were infected with influenza. 45 days later, mice were injected in vivo with anti-CD45 antibodies prior to collection of blood and lung. When administered, FTY720 was injected i.v. 2 days prior to analysis. (a) Representative FACS plots showing abundance of memory/activated B cells (defined as Live B220$^+$ CD38$^+$ EYFP$^+$) in blood of FTY720 treated and untreated mice. (b) Memory/activated B cells (defined as in 'a') and naïve B cell (defined as Live B220$^+$ CD38$^+$ YFP$^-$) counts within blood. (c) Representative FACS plots of lung BRM cells (identified as Live B220$^+$ i.v. CD45$^-$ CD69$^+$ YFP$^+$ GL7$^-$). (d) Summary of lung BRM cell and total lung B cell (Live B220$^+$ i.v. CD45$^-$) frequencies in FTY720 treated and untreated animals. Data shows results of one experiment out of 2 performed. Each circle represents one mouse.

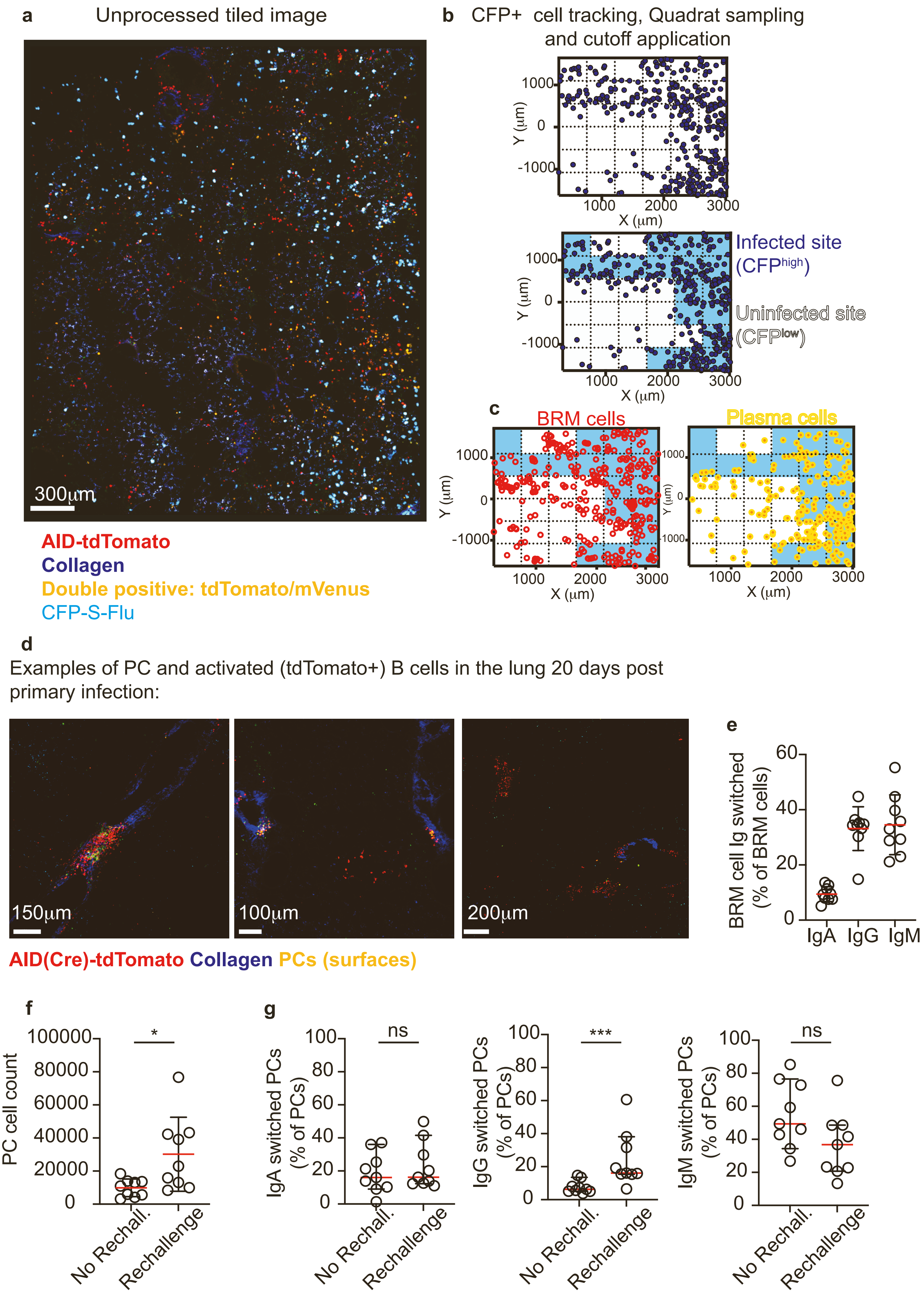
a
Unprocessed tiled image
300μm
AID-tdTomato
Collagen
Double positive: tdTomato/mVenus
CFP-S-Flu
b
CFP+ cell tracking, Quadrat sampling and cutoff application
Y (μm)
X (μm)
Infected site (CFPhigh)
Uninfected site (CFPlow)
c
BRM cells
Plasma cells
d
Examples of PC and activated (tdTomato+) B cells in the lung 20 days post primary infection:
150μm
100μm
200μm
AID(Cre)-tdTomato Collagen PCs (surfaces)
e
BRM cell Ig switched (% of BRM cells)
IgA IgG IgM
f
PC cell count
*
No Rechall.
Rechallenge
g
IgA switched PCs (% of PCs)
ns
IgG switched PCs (% of PCs)
***
IgM switched PCs (% of PCs)
ns

Supplementary Fig. 3 Visualization and quantification of PC distribution in influenza infected lungs form BAT mice, related to Fig.3

(a-c) Analysis pipeline for defining infected and uninfected sites and distribution of BRM and PCs within these sites. (a) An example of an unprocessed image of a tiled static lung section collected by TPLSM, 4 days post rechallenge of BAT mice with CFP-S-Flu. Collagen was captured in the second harmonic channel (blue). (b) Spatial point patterns, showing CFP infected cell tracking and positioning (black dots, top). The image was partitioned into sectors, as shown, and the density of CFP^+ cells within each sector was quantified to assign it a value as infected or uninfected (bottom). (c) BRM cells and PCs were identified (red and yellow circles, respectively) and their densities in infected (blue) or uninfected (white) sites was determined. (d) Distribution of PCs during primary infection. Large tiles of static lung sections from BAT mice imaged using TPLSM 20 days post primary infection. At this time point, PCs are detected in the lungs, but they are confined to PC clusters and iBALT-like structures and are not observed within alveoli. Images are representative of 3 mice imaged in 3 independent experiments. (e) Frequencies of IgA, IgM and a combination of IgG1, IgG2b and IgG2a[b] (collectively referred to as ‘IgG’) isotypes within the BRM cell subset (defined as i.v. $CD45^-$ $B220^+$ $GL7^-$ $tdTomato^+$ $mVenus^-$) in the lung of BAT mice during the memory phase (>6 weeks post primary infection). (f, g) Numbers (f) and Ig isotype frequencies (g) of lung PCs in BAT mice before and 4 days post rechallenge. PCs were defined as i.v. $CD45^-$ $GL7^-$ $tdTomato^+$ $mVenus^+$. Frequencies of IgA, IgM and IgG (including IgG1, IgG2b and IgG2a[b]) within the total PC subset were defined based on intracellular stain with the indicated Ig isotype. Figure shows pooled results of one from 3 independent experiments performed. Each circle represents one mouse.

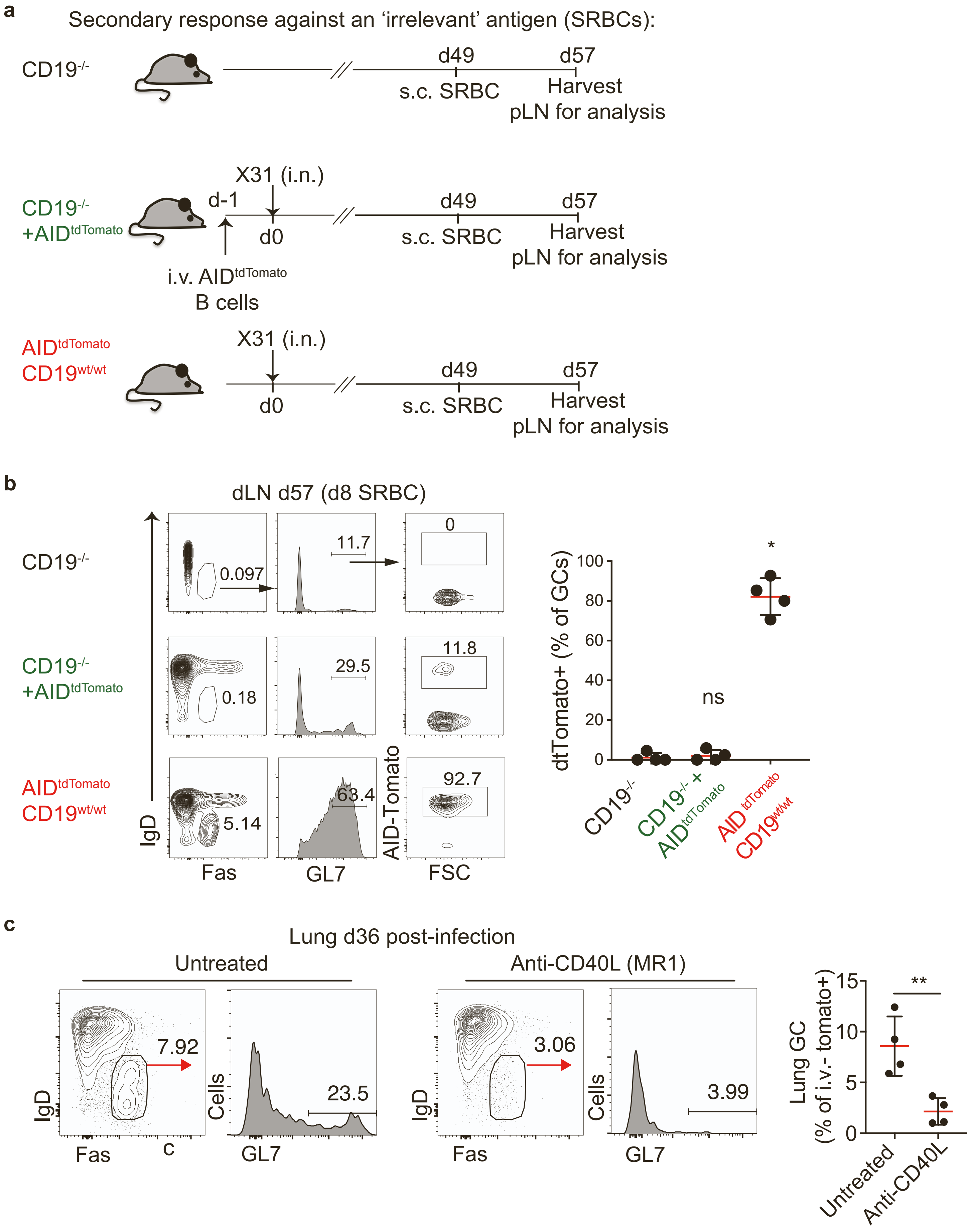
a
Secondary response against an 'irrelevant' antigen (SRBCs):
CD19-/-
d49
d57
s.c. SRBC
Harvest
pLN for analysis
CD19-/-
+AIDtdTomato
X31 (i.n.)
d-1
d0
i.v. AIDtdTomato
B cells
AIDtdTomato
CD19wt/wt
b
dLN d57 (d8 SRBC)
0.097
11.7
0
0.18
29.5
11.8
5.14
63.4
92.7
IgD
Fas
GL7
AID-Tomato
FSC
dtTomato+ (% of GCs)
100
80
60
40
20
0
*
ns
CD19-/-
CD19-/- +
AIDtdTomato
AID tdTomato
CD19wt/wt
c
Lung d36 post-infection
Untreated
Anti-CD40L (MR1)
7.92
23.5
3.06
3.99
IgD
Fas
Cells
GL7
Lung GC
(% of i.v.- tomato+)
15
10
5
0
**
Untreated
Anti-CD40L

Supplementary Fig. 4. Validation of approaches to track reactivation of memory B cells in the lung, related to Fig.5

(a-b) Validation of the CD19$^{-/-}$ transfer system as an effective approach to track memory, but not naïve B cell reactivation. Experimental outline. To generate mice in which only transferred labelled cells can generate memory B cell responses, CD19$^{-/-}$ mice were adoptively transferred with CD19^{WT} AID-Cre^{tdTomato} B cells. CD19-/- mice without adoptive transfers were used as negative controls (for mice that cannot generate memory B cells). As a positive control (for mice that are able to generate endogenous memory B cells), we used CD19^{WT} B cells from AID-Cre^{tdTomato} mice. Animals were infected with influenza virus and 45 days later were rechallenged s.c. with SRBCs. 8d later draining LNs were collected for analysis of GC B cells. (b) Left, representative FACS plots and gating strategy to identify tdTomato^{+} cells within GC (defined as IgD^{low} Fas^{+} GL7^{+} tdTomato^{+}). Plots are pre-gated on B220^{+} live cells. Right, quantification of frequencies of tdTomato^{+} cells within GC gates. Figure shows results from one experiment from two performed. Each circle represents one mouse. (c) Validation of anti-CD40L antibody treatment. Mice were infected with X31 influenza and on d26 started treatment with i.v. anti-CD40L antibodies for 10 days as illustrated in Fig. 5e. Mice were in vivo labelled with anti-CD45 4 mins prior to collection of lungs. The frequencies of lung GC B cells were determined by FACS, identified as i.v. CD45^{-} tdTomato^{+} IgD^{low} Fas^{+} GL7^{+} GC B cells, as indicated. Left, shown representative plots, pre-gated on B220^{+} i.v. CD45^{-} live cells. Right, quantification of GC B cell frequencies of i.v. CD45^{-} tdTomato^{+} gate. Figure shows results from one experiment from two performed. Each circle represents one mouse.

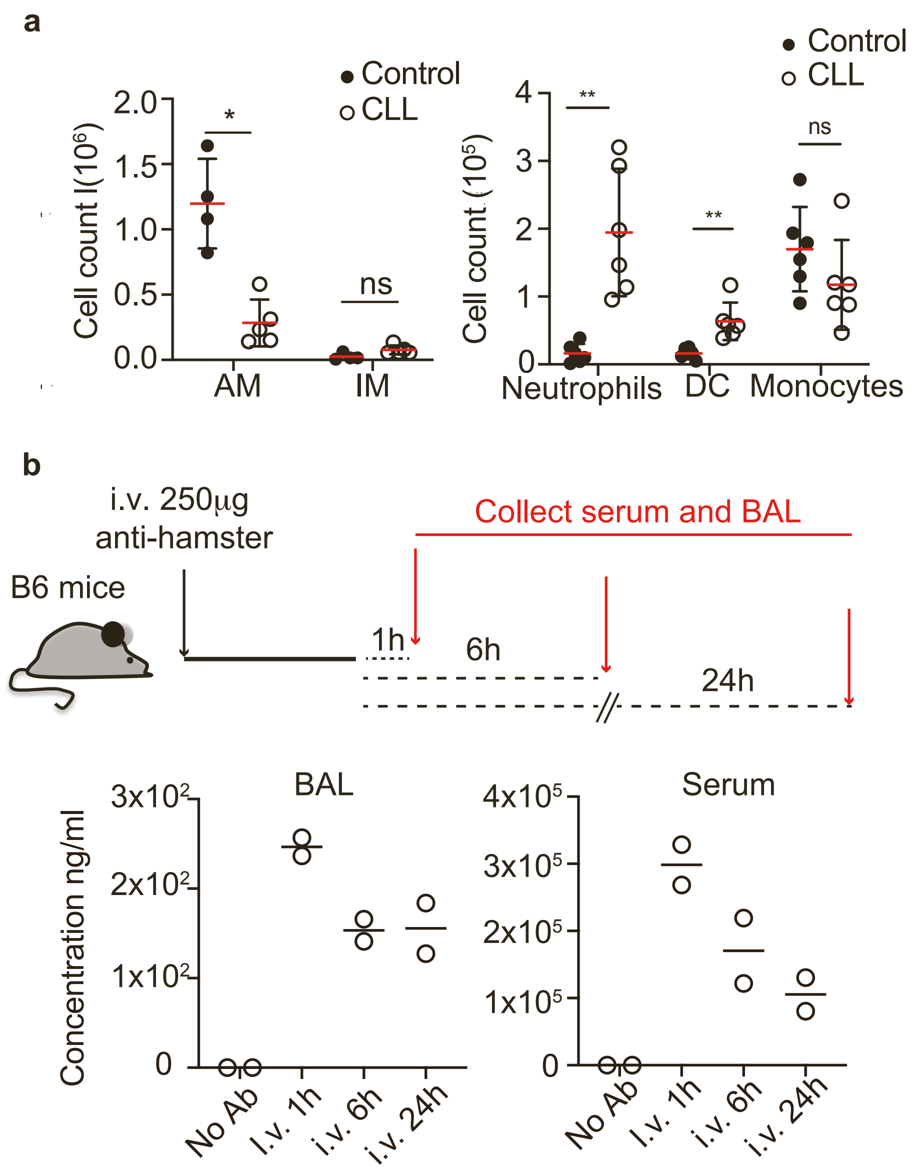

Supplementary Fig.5. Ablation of alveolar macrophages using CLL and kinetics of serum antibody saturation in the BAL, related to Fig.6

(a) Cell counts for alveolar macrophages (AM, i.v. CD45$^-$, ex vivo CD45$^+$ F4/80$^+$ CD11c$^+$ CD11b$^-$ SiglecF$^+$), interstitial macrophages (IM, i.v. CD45$^-$, ex vivo CD45$^+$ F4/80$^+$ CD11c$^+$ CD11b$^+$ SiglecF$^-$), neutrophil (i.v. CD45$^-$, ex vivo CD45$^+$ Ly6G$^+$ CD11b$^+$), dendritic cell (i.v. CD45$^-$, ex vivo CD45$^+$ F4/80$^-$ CD11c^{hi}) and monocyte (i.v. CD45$^-$, ex vivo CD45$^+$ Ly6G$^-$ Ly6C$^+$) in total lungs derived from memory mice treated intranasally with CLL 6 and 3 days prior to tissue collection. Figure shows results of one from three independent experiments performed. (b) Defining the kinetics of i.v. injected antibodies infiltration of the BAL. Top, experimental set up. Mice were injected i.v. with 250μg anti-hamster antibodies. 1, 6 and 24 hours later, blood and BAL were collected. The concentrations of hamster IgG in these compartments were determined by ELISA. Figure shows results of one from two independent experiments performed. Each circle represents one mouse.

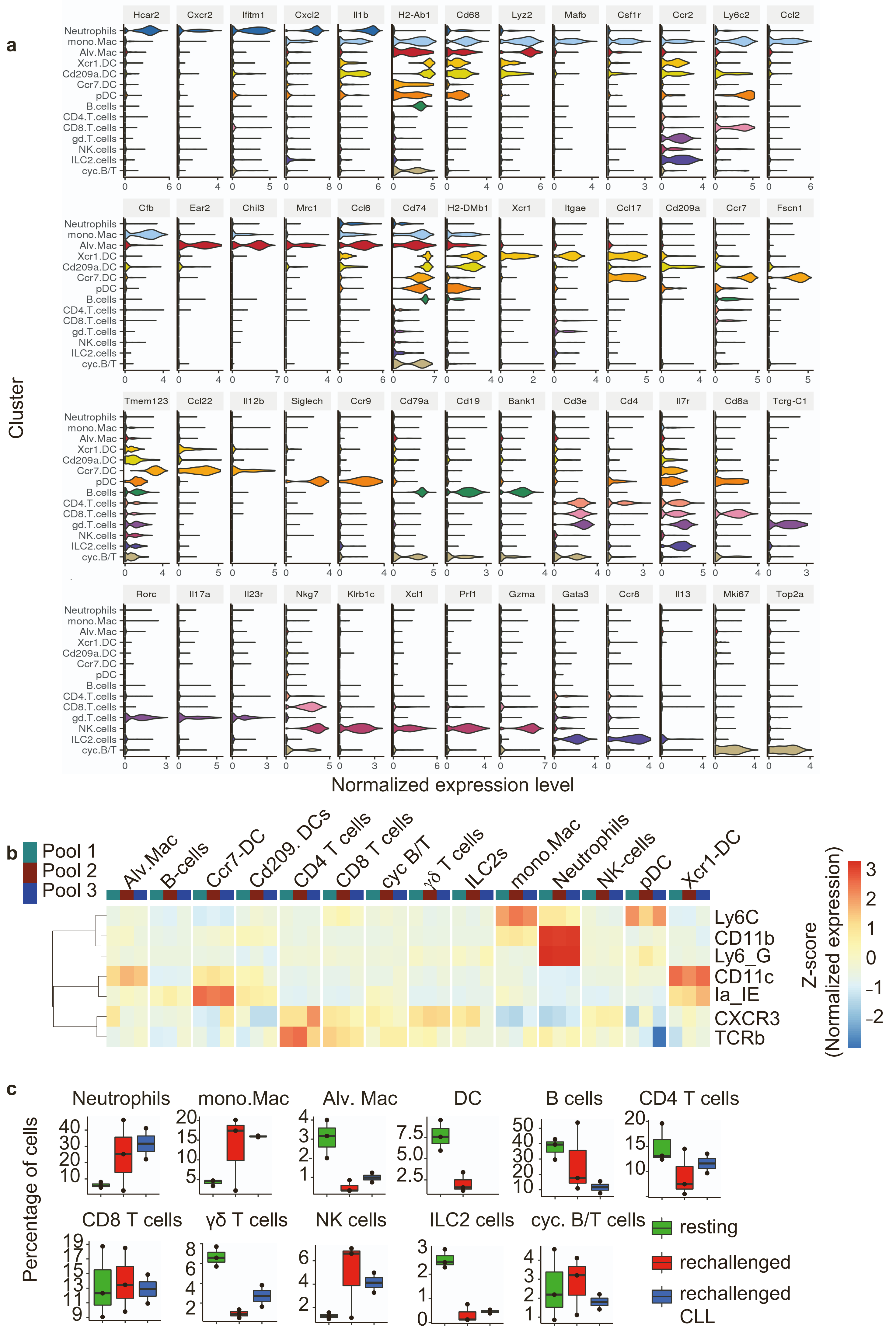
a
Cluster
Neutrophils
mono.Mac
Alv.Mac
Xcr1.DC
Cd209a.DC
Ccr7.DC
pDC
B.cells
CD4.T.cells
CD8.T.cells
gd.T.cells
NK.cells
ILC2.cells
cyc.B/T
Hcar2
Cxcr2
Ifitm1
Cxcl2
Il1b
H2-Ab1
Cd68
Lyz2
Mafb
Csf1r
Ccr2
Ly6c2
Ccl2
Cfb
Ear2
Chil3
Mrc1
Ccl6
Cd74
H2-DMb1
Xcr1
Itgae
Ccl17
Cd209a
Ccr7
Fscn1
Tmem123
Ccl22
Il12b
Siglech
Ccr9
Cd79a
Cd19
Bank1
Cd3e
Cd4
Il7r
Cd8a
Tcrg-C1
Rorc
Il17a
Il23r
Nkg7
Klrb1c
Xcl1
Prf1
Gzma
Gata3
Ccr8
Il13
Mki67
Top2a
Normalized expression level
b
Pool 1
Pool 2
Pool 3
Alv.Mac
B-cells
Ccr7-DC
Cd209. DCs
CD4 T cells
CD8 T cells
cyc B/T
γδ T cells
ILC2s
mono.Mac
Neutrophils
NK-cells
pDC
Xcr1-DC
Ly6C
CD11b
Ly6_G
CD11c
Ia_IE
CXCR3
TCRb
Z-score
(Normalized expression)
c
Percentage of cells
Neutrophils
mono.Mac
Alv. Mac
DC
B cells
CD4 T cells
CD8 T cells
γδ T cells
NK cells
ILC2 cells
cyc. B/T cells
resting
rechallenged
rechallenged CLL

Supplementary Fig.6 Changes in the cellular landscape of rechallenged mice in the presence and absence of alveolar macrophages, related to Fig.7

scRNA-seq analysis of lung leukocytes from mice treated as described in Figs. 7a-c. (a) Violin plots showing marker genes for the cell clusters that are conserved between the three experimental conditions. (b) Heatmap showing the expression of surface proteins by cluster (CITE-seq analysis). (c) Box plots showing the frequencies of the different cell types by experimental group.

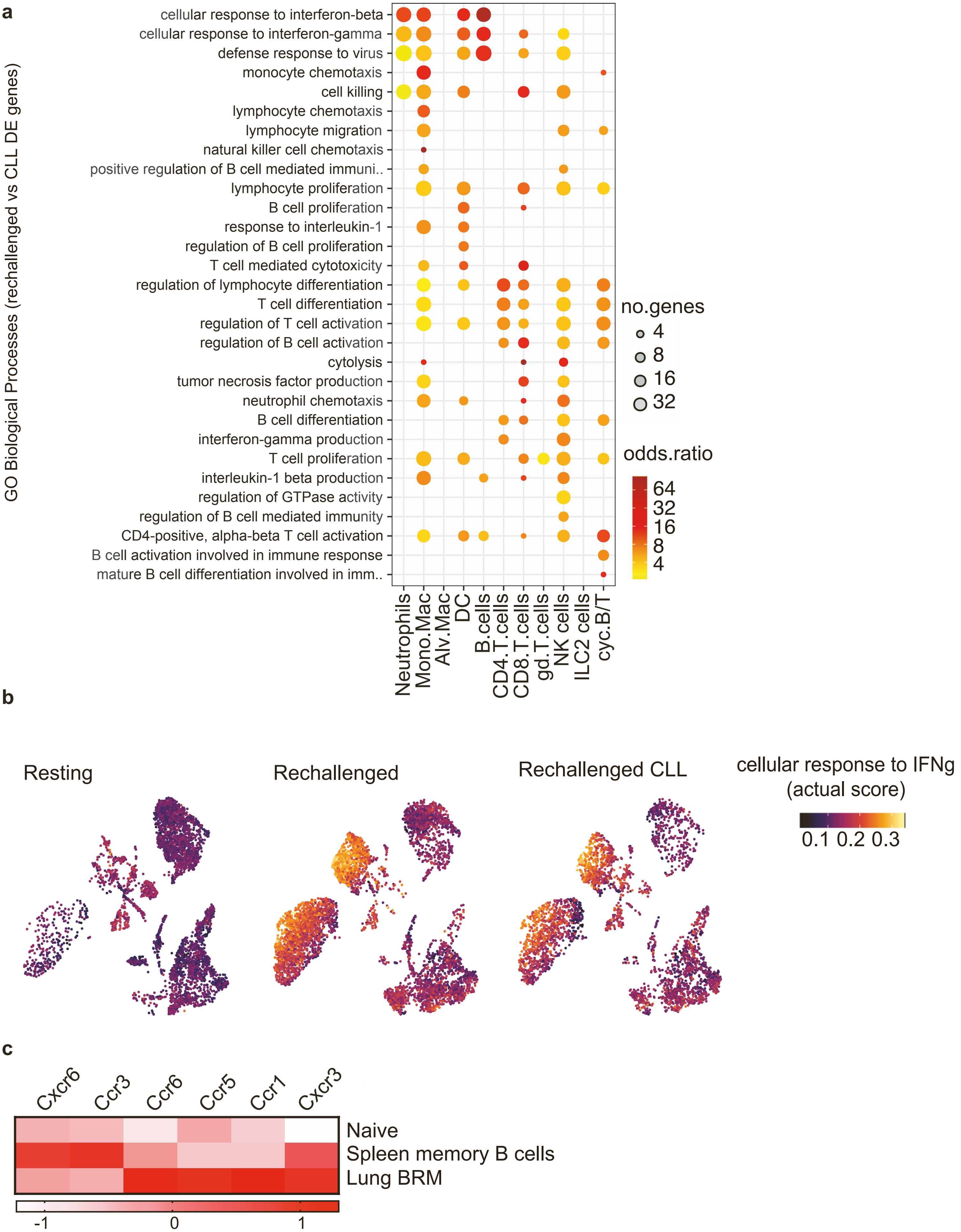
a
GO Biological Processes (rechallenged vs CLL DE genes)
cellular response to interferon-beta
cellular response to interferon-gamma
defense response to virus
monocyte chemotaxis
cell killing
lymphocyte chemotaxis
lymphocyte migration
natural killer cell chemotaxis
positive regulation of B cell mediated immuni..
lymphocyte proliferation
B cell proliferation
response to interleukin-1
regulation of B cell proliferation
T cell mediated cytotoxicity
regulation of lymphocyte differentiation
T cell differentiation
regulation of T cell activation
regulation of B cell activation
cytolysis
tumor necrosis factor production
neutrophil chemotaxis
B cell differentiation
interferon-gamma production
T cell proliferation
interleukin-1 beta production
regulation of GTPase activity
regulation of B cell mediated immunity
CD4-positive, alpha-beta T cell activation
B cell activation involved in immune response
mature B cell differentiation involved in imm..
Neutrophils
Mono.Mac
Alv.Mac
DC
B.cells
CD4.T.cells
CD8.T.cells
gd.T.cells
NK cells
ILC2 cells
cyc.B/T
no.genes
4
8
16
32
odds.ratio
64
32
16
8
4
b
Resting
Rechallenged
Rechallenged CLL
cellular response to IFNg
(actual score)
0.1 0.2 0.3
c
Cxcr6
Ccr3
Ccr6
Ccr5
Ccr1
Cxcr3
Naive
Spleen memory B cells
Lung BRM
-1
0
1

Supplementary Fig.7 Depletion of alveolar macrophages leads to impaired myeloid cell IFNG response, related to Fig.7

scRNA-seq analysis of lung leukocytes from mice treated as described in Figs. 7a-c. (a) Dotplots showing selected Gene Ontology (GO) Biological Processes (BP) that are significantly over-represented in genes differentially expressed between the CLL treated rechallenge and rechallenge conditions within the cell types (One sided Fisher's exact tests; BH adjusted $p < 0.05$). (b) UMAPs showing the expression of the GO BP "cellular response to IFNG" geneset in the cells from each of the three conditions (AUCell analysis). (c) Heatmap showing qPCR expression of the indicated inflammatory chemokine receptors from sorted naïve B cells, splenic memory B cells and lung BRM cells. Data in c represent analysis of the same experiment described in Fig. 1b.
