# Supplementary material for: Secondary influenza challenge triggers resident memory B cell migration and rapid relocation to boost antibody secretion at infected sites
Source: Immunity. 2022 Apr 12;55(4):718–733.e8. doi: 10.1016/j.immuni.2022.03.003 (PMC9044924; doi:10.1016/j.immuni.2022.03.003)
Supplement: Document S2. Article plus supplemental information [file mmc8.pdf]

Article

Immunity

Secondary influenza challenge triggers resident memory B cell migration and rapid relocation to boost antibody secretion at infected sites

Graphical abstract

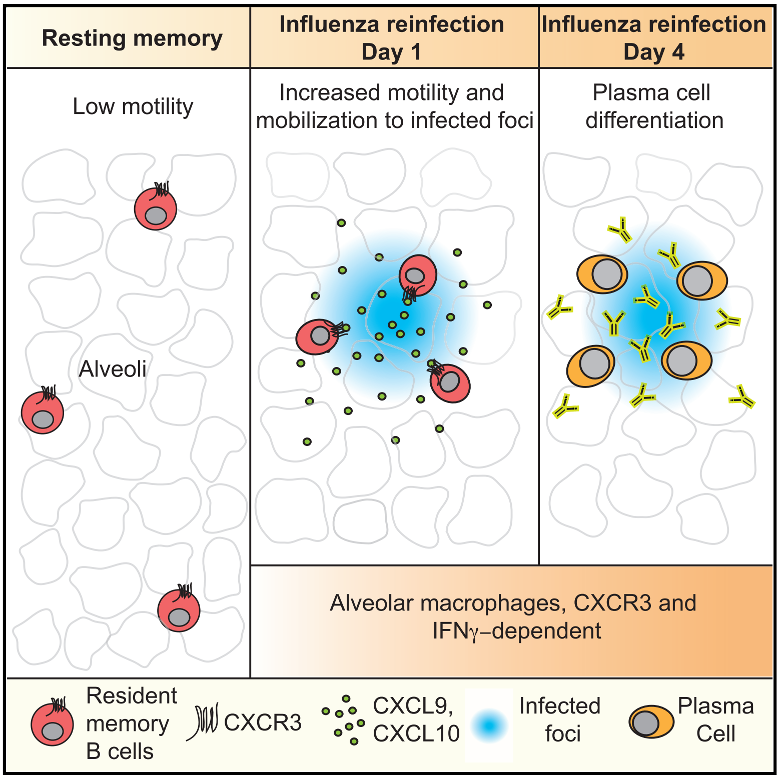

Highlights

- Lung BRM cells localize to the alveoli and display confined probing behavior
- Upon rechallenge, BRM cells increase their motility and accumulate in infected foci
- BRM cell mobilization is followed by PC formation in sites of viral replication
- Alveolar macrophages orchestrate this process via induction of IFNγ and CXCR3 ligands

Authors

Andrew J. MacLean, Niamh Richmond, Lada Koneva, ..., Stephen N. Sansom, Oliver Bannard, Tal I. Arnon

Correspondence

oliver.bannard@ndm.ox.ac.uk (O.B.), tal.arnon@kennedy.ox.ac.uk (T.I.A.)

In brief

Resident memory B cells develop and persist in the lungs of influenza-infected hosts. Using 3D and live-imaging analysis of explant lung sections, MacLean et al. uncover cellular and molecular mechanisms that orchestrate the activation of these cells during secondary infection, leading to rapid delivery of antibodies in a highly localized manner directly to sites of viral replication.

MacLean et al., 2022, Immunity *55*, 718–733
April 12, 2022 © 2022 The Author(s). Published by Elsevier Inc.
https://doi.org/10.1016/j.immuni.2022.03.003

CellPress

CellPress
OPEN ACCESS

Immunity

Article

Secondary influenza challenge triggers resident memory B cell migration and rapid relocation to boost antibody secretion at infected sites

Andrew J. MacLean,[1] Niamh Richmond,[1] Lada Koneva,[1] Moustafa Attar,[1] Cesar A.P. Medina,[1] Emily E. Thornton,[2] Ariane Cruz Gomes,[3] Aadil El-Turabi,[3] Martin F. Bachmann,[3,4] Pramila Rijal,[2] Tiong Kit Tan,[2] Alain Townsend,[2] Stephen N. Sansom,[1] Oliver Bannard,[2,*] and Tal I. Arnon[1,5,*]
[1]University of Oxford, Kennedy Institute of Rheumatology, Oxford, UK
[2]University of Oxford, MRC Human Immunology Unit, MRC Weatherall Institute of Molecular Medicine, Oxford, UK
[3]University of Oxford, The Jenner Institute, Nuffield Department of Medicine, Oxford, UK
[4]University of Bern, Rheumatology, Immunology and Allergology, Department of BioMedical Research, Bern, Switzerland
[5]Lead contact
*Correspondence: oliver.bannard@ndm.ox.ac.uk (O.B.), tal.arnon@kennedy.ox.ac.uk (T.I.A.)
https://doi.org/10.1016/j.immuni.2022.03.003

SUMMARY

Resident memory B (BRM) cells develop and persist in the lungs of influenza-infected mice and humans; however, their contribution to recall responses has not been defined. Here, we used two-photon microscopy to visualize BRM cells within the lungs of influenza -virus immune and reinfected mice. Prior to re-exposure, BRM cells were sparsely scattered throughout the tissue, displaying limited motility. Within 24 h of rechallenge, these cells increased their migratory capacity, localized to infected sites, and subsequently differentiated into plasma cells. Alveolar macrophages mediated this process, in part by inducing expression of chemokines CXCL9 and CXCL10 from infiltrating inflammatory cells. This led to the recruitment of chemokine receptor CXCR3-expressing BRM cells to infected regions and increased local antibody concentrations. Our study uncovers spatiotemporal mechanisms that regulate lung BRM cell reactivation and demonstrates their capacity to rapidly deliver antibodies in a highly localized manner to sites of viral replication.

INTRODUCTION

Influenza virus is a common airborne pathogen that infects cells of the respiratory tract. Despite progress in available treatments, influenza continues to present a significant medical burden and poses the risk of causing global pandemics similar to the one seen in 1918, which was responsible for over 40 million deaths (Saunders-Hastings and Krewski, 2016). While T cells are essential for clearance of the virus, pre-existing antibodies can provide sterilizing immunity and prevent spread from initial sites of viral infection (Chiu et al., 2015; Krammer, 2019; Sallusto et al., 2010). Pioneering studies conducted more than 100 years ago demonstrate that transfer of serum from immunized animals into naive hosts protects them even from lethal doses of influenza strains. Importantly, the most effective results were obtained when the antibodies were delivered directly into the lower airways (Weltzin and Monath, 1999). These and subsequent studies not only established the potential of antibodies to provide immunity against influenza but also demonstrate the importance of antibodies being localized to potential sites of infection as a major factor in achieving optimal results. Better understanding of the mechanisms that increase antibody titers locally within the lung may therefore help to guide the development of new and more effective vaccine strategies to prevent the spread of influenza variants.

Following primary infection or vaccination, B cells that are specific for virally derived antigens are activated in secondary lymphoid organs (SLOs) where they may subsequently mature the affinity of their antibodies through iterative rounds of activation-induced cytidine deaminase (AID)-catalyzed somatic hypermutation and selection in germinal centers (GCs) (Bannard and Cyster, 2017; Victora and Wilson, 2015). A subset of memory B cells emerges from this pathway and joins naive B cells that recirculate between SLOs, continuously scanning the body for secondary infection (Good-Jacobson and Tarlinton, 2012; Phan and Tangye, 2017; Shlomchik and Weisel, 2012; Suan et al., 2017). GC-derived plasma cells (PCs), which also develop during primary infection may migrate to the bone marrow, where they occupy defined niches and constitutively secrete antibodies to maintain serum concentrations (Cornelis et al., 2021; Ulbricht et al., 2021). However, recent work shows that, in addition to recirculating memory B cells, another memory B cell population expressing high levels of the tissue residency-associated marker, CD69, accumulates and persists in the lungs of influenza-infected mice for many months after viral clearance (Adachi et al., 2015; Joo et al., 2008; Onodera et al., 2012). The possibility

 © 2022 The Author(s). Published by Elsevier Inc.
This is an open access article under the CC BY license (http://creativecommons.org/licenses/by/4.0/).

CellPress
OPEN ACCESS

that these cells represent a distinct tissue-resident memory B (BRM) cell subset was confirmed by elegant experiments using parabiotic mice that demonstrate the nonrecirculatory nature of these cells and their ability to survive in the tissue for prolonged periods independently of input from the circulation (Allie et al., 2019; Allie and Randall, 2020). The strategic positioning of BRM cells near portals of viral entry suggests a superior capacity to promote rapid increase in local antibody concentrations and to confer long-lasting protection from infection. In line with this possibility, reactivation of BRM cells in the lungs has been suggested to lead to a rapid increase in local PC differentiation (Allie et al., 2019; Barker et al., 2021; Joo et al., 2008; Onodera et al., 2012). However, the cellular and molecular mechanisms that orchestrate this process have not been defined.

Here, we address these questions by investigating the spatiotemporal regulation of lung BRM cells, during immune phases and after secondary influenza-viral infection. Our study reveals the dynamic behaviors and molecular events that regulate humoral immunity in the lungs, and uncovers an unexpected migratory step that allows rapid and highly localized production of antibodies directly within sites of viral infection.

RESULTS

A fate-mapping approach for tracking lung-resident memory B cells

BRM cells have been detected inside the lung tissue of influenza-infected mice within areas that are sheltered from the blood, but the microanatomical sites of their residency are unknown. To address this question, we established a mouse model to track lung BRM cells *in situ*. To label previously activated B cells, we utilized *Aicda*(AID)$^{Cre/+}$ Rosa26^{tdTomato} reporter mice, in which B cells are irreversibly labeled after activation by expression of the fluorescent protein tdTomato (Roco et al., 2019; Rommel et al., 2013). We further crossed the mice to a *Prdm1*^{mVenus} (Blimp1^{mVenus}) strain (Ohinata et al., 2008), in which mVenus expression under the *Prdm1* regulator elements permits detection of PCs. We refer to these triple positive animals as Blimp1^{mVenus} AID$^{Cre/+}$ Rosa26^{tdTomato} ("BAT") mice (Figure 1A).

Six weeks after influenza infection, we identified a distinct tdTomato^{+} mVenus^{neg} GL7 (GC marker)^{neg} pulmonary B cell population that was sheltered from *in vivo* labeling following an intravenous injection of anti-CD45 (i.v. CD45) antibody shortly before tissue harvest. These cells exhibited a distinct phenotype compared with naive (B220^{+} tdTomato^{neg} mVenus^{neg} GL7^{neg}) and splenic memory (tdTomato^{+} mVenus^{neg} GL7^{neg}) B cells, including elevated surface-protein expression of CD69, CXCR3, CD80, and PDL2 as well as downregulation of CD62L (Figures 1B and 1C). An expression profile consistent with this phenotype was also observed when lung GL7^{-} tdTomato^{+} B cells specific for a hemagglutinin (HA) probe (Figures S1A–S1C) and which express the canonical residency marker CD69^{+}, were isolated and subjected to RNA-seq (gating scheme shown in Figure S1D). While all populations shared core B cell signatures (Figure S1E), differential mRNA gene expression and principal component analysis (PCA) indicated that specific differences between them exist (Figures 1D–1F; Table S1), possibly reflecting the unique positional characteristics and functions of each population. The localization of the cells in parenchymal sites, and the similarity of the above staining and gene expression characteristics to those of the nonrecirculatory memory B cell subset described by Allie et al. (2019), support that they are tissue resident. Consistent with this, these lung memory B cells showed selective downregulation of mRNA transcripts coding for *S1pr1*, a key receptor that controls lymphocyte recirculation by facilitating entry to the blood and lymph (Cyster and Schwab, 2012) and persisted in the tissue for many months after primary infection (Figure 1G). As expected from previous work (Adachi et al., 2015; Allie et al., 2019; Joo et al., 2008; Onodera et al., 2012), GC B cells (i.v. CD45^{-} B220^{+} Blimp-1^{-} GL7^{+}) were also present in the lungs of convalescent mice (Figure S1D), as were PCs co-expressing tdTomato and mVenus (Figures 1G and S1D). Thus, the above BAT reporter system allows tracing of lung memory B cells and PCs effectively, with the capacity to distinguish them from GC B cells.

Resident memory B cells transition from low to high motility upon rechallenge

To elucidate the distribution and cellular dynamics of lung BRM cells during the "memory phase" (>6 weeks postinfection), we performed live imaging of explanted lung sections using two-photon microscopy (Thornton et al., 2012). Aggregates of tdTomato^{+} cells were occasionally identified adjacent to bronchovascular bundles (Figures 2A, top left, and S1F). Within these inducible bronchus-associated lymphoid tissues (iBALT)-like structures, the cells displayed the typical extensive but confined motility behavior associated with GC B cell characteristics (Figure S1F; Video S1; Allen et al., 2007; Hauser et al., 2007; Schwickert et al., 2007). In agreement, confocal microscopy analysis indicated these cells expressed the GC B cell marker, GL7 (Figure S1G). PCs were also confined to large clusters found primarily around branching points of large airways (Figure 2A, top center), or contained within the outer perimeter of iBALT-like structures. In these locations, PCs had a rounded morphology consistent with the sessile nature of PCs (Allen et al., 2007; Fooksman et al., 2010; Schwickert et al., 2007; Zehentmeier et al., 2014; Figure S1F; Video S1). In contrast, many tdTomato^{+} mVenus^{-} cells did not aggregate but were instead distributed sparsely throughout the lung parenchyma in close contact with alveoli (Figure 2A, top right). In contrast to the high motility of tdTomato^{+} mVenus^{-} B cells within iBALT, these parenchymal BRM cells exhibited limited migratory capacity and were often seen to perform restricted surveillance behavior, locally probing alveolar walls (Figure 2A, bottom; Video S2).

To study how lung BRM cells become activated, we visualized them in live explant sections after rechallenge. Within 24 h after secondary infection, the cells doubled their mean migration speeds and were seen to displace across longer distances (Figures 2B–2E; Video S3). Occasionally, we detected cells undergoing the transition from low to high motility, supporting the notion that these highly migratory cells are derived from local lung BRM cells, rather than cells recruited from the blood (Figure 2C; Video S3). To further test this hypothesis, we treated mice with the S1PR1 agonist, FTY720, 2 days prior to rechallenge to "lock" recirculating cells within SLOs (Cyster and Schwab, 2012). Flow-cytometry analysis confirmed that this treatment sequestered naive and memory B cells from the blood (Figure S2). FTY720 administration did not inhibit the ability of

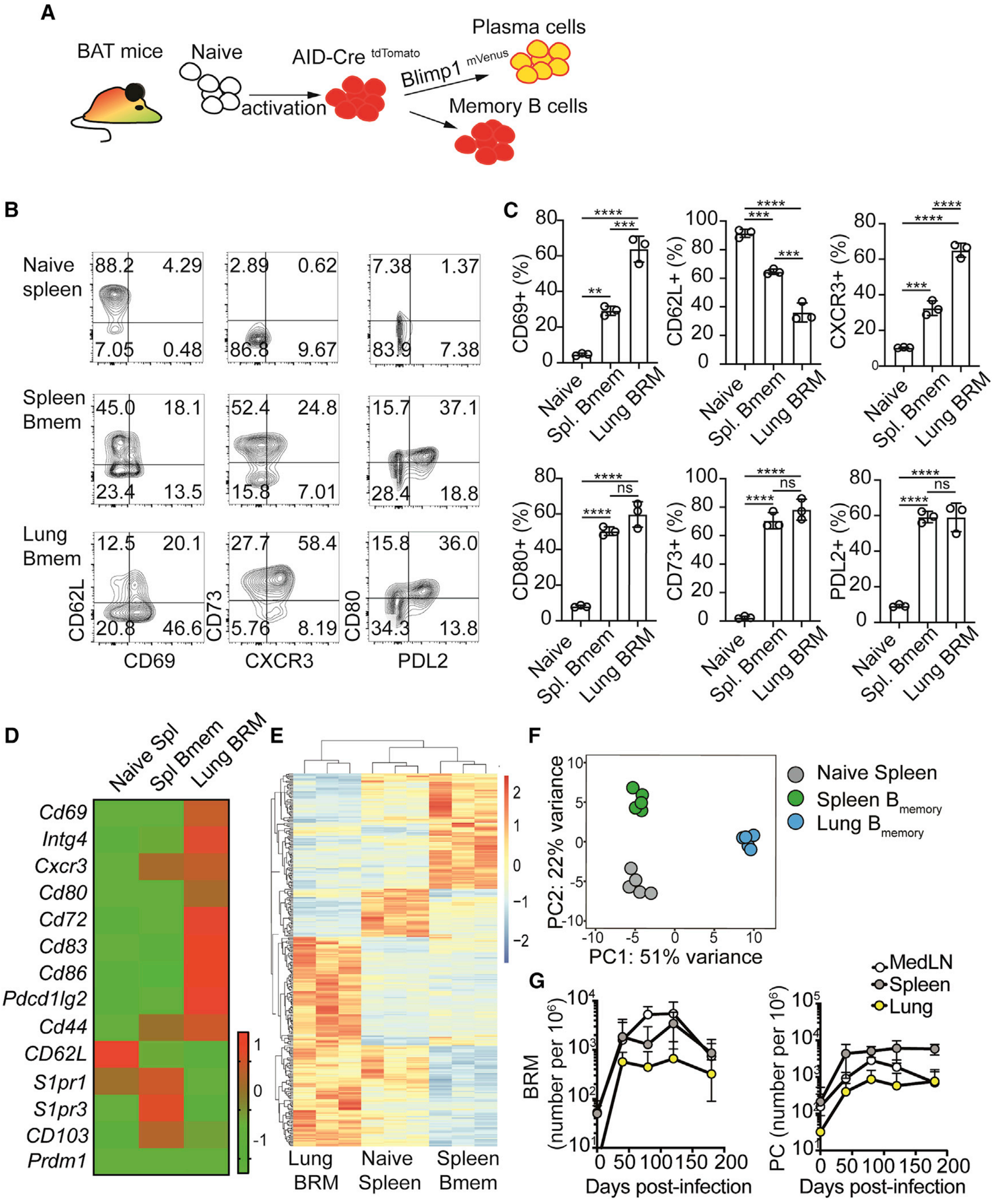

Figure 1. Tracking lung-resident memory B cells

(A) Schematic of BAT reporter mice.

(B) FACS plots of naive (B220^{+} tdTomato^{neg} mVenus^{neg} GL7^{neg}) and memory (tdTomato^{+} mVenus^{neg} GL7^{neg}) cells from spleens and lungs of BAT mice 6 weeks postinfection. In the lung, plots are also pregated on parenchymal cells (*in vivo* CD45^{neg}).

(C) Frequencies of cells expressing the indicated markers gated as in (B). Data represent one of 3 independent experiments. Statistical analysis was made using an ordinary one-way ANOVA. Error bars represent SD. **p < 0.01; ***p < 0.001; ****p < 0.0001.

(D–F) Bulk RNA-seq of naive B cells and HA^{+} memory B cells from lung and spleen derived from BAT mice. Lung B cells were also pre-gated on parenchymal CD69^{+} cells. Gating shown in Figure S1D. (D) Heatmap of key migration and residency genes. (E) Full heatmaps of all differentially expressed genes with an adjusted p value (FDR) < 0.05. (F) PCA plots, applied to the top 500 most variable genes. Shown are data from 3 samples collected in 3 independent experiments (n = 6 per experiment).

(G) HA specific memory B cell, as defined as in (E and F) and PC numbers (gating shown in Figure S1D) in BAT mice, quantified over time. Data in (G) are pooled from 6 independent experiments with 4–6 mice per group. See also Figure S1.

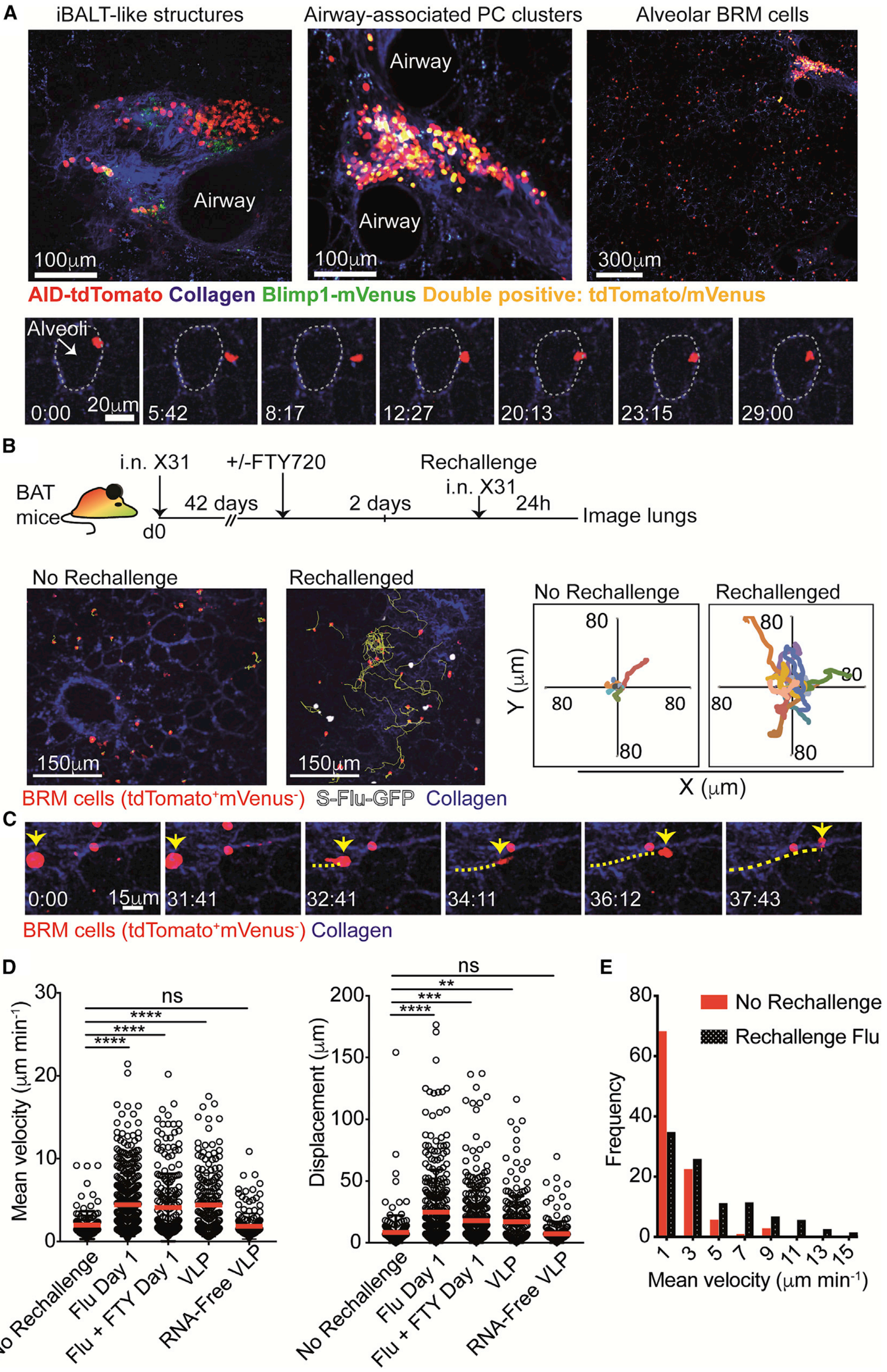

(legend on next page)

BRM cells to increase their motility after rechallenge, indicating that they were derived from a local, rather than a systemic source (Figure 2D).

The rapid increase in alveolar BRM motility shortly after rechallenge prompted us to ask whether this effect depends on recognition of cognate antigens, or if other mechanisms are involved. To address this, we performed experiments similar to those described above but this time swapped the viral rechallenge for a mock infection with Qβ virus-like particles (VLPs). These self-assembling protein structures share many molecular characteristics with live virions, but express no shared antigens with the influenza virus (Bachmann and Jennings, 2010). Live imaging at 24 h post-VLP challenge revealed rapid increases in the motility of alveoli-associated BRM cells, similar to that observed following rechallenge with the live virus (Figure 2D). This effect depended on the presence of RNA because it was lost when we used RNA-free VLPs (Figure 2D). Taken together, our findings demonstrate that during recall responses, local lung BRM cells rapidly increase their migration speeds in a manner that is independent of antigen specificity but may involve recognition of danger signals through innate sensing pathways.

Resident memory B cells cluster into foci of infection early after reactivation

During analysis we noticed that the overall distribution of the BRM cells changed after rechallenge, forming visible "patches" containing high densities of cells. To quantitatively assess this, we acquired multiple large tiles of lung sections and analyzed them using the spatial statistics function Ripley's K. By comparing the normalized value of Ripley's K (L values) of experimental data with those calculated for a simulated random distribution, we obtained a defined measure of cell clustering (Jafari-Mamaghani et al., 2010; Kiskowski et al., 2009; Ripley, 1977). We used two-photon microscopy for this analysis, to allow detection of large numbers of cells from 3D imaging volumes (~150 μm). Prior to rechallenge, the measured L values of alveolar BRM cells along a range of radiuses were consistent with a random pattern of positioning (Figure 3A, left). Upon rechallenge, these values increased, indicating a shift toward a nonrandom distribution (Figure 3A, right). To compare these parameters across multiple mice and conditions, we calculated the L value at a defined radial distance (200 μm) (Figure 3B). This analysis further indicated a consistent tendency of activated alveolar BRM cells to redistribute in a nonrandom manner, an effect that was already evident within 24 h of rechallenge and which persisted for at least 4 days. Importantly, pretreatment with FTY720 did not impact this behavior, indicating that it was independent of circulating B cell recruitment (Figure 3B).

We next asked whether BRM cell repositioning reflects preferential association with sites of infection. To test this, we generated a CFP-expressing influenza strain, leading to the labeling of infected cells with the fluorescent CFP protein (CFP-S-Flu) (Powell et al., 2012). Lung sections were collected and analyzed 24 h post rechallenge. Infected foci were defined as sites in which the density of CFP^+ cells was >225 per mm^3 and the frequencies of BRM cells within these regions were measured (Figures S3A–S3C). Applying this analysis across multiple mice and sections revealed a consistent tendency of alveolar BRM cells to accumulate in highly infected foci, with an average of 2- to 3-fold higher density measured in these sites (Figures 3C and 3D). Injection of pertussis toxin (PTX), which blocks signaling via Gi protein-coupled receptors (GPCRs), 2 h after rechallenge completely inhibited this effect. Under these conditions BRM cells maintained their homogenous distribution and displayed similar densities within infected and uninfected regions (Figures 3E–3G). Thus, the relocalization of BRM cells to infected sites is an active process that is likely regulated by the engagement of chemotactic receptors.

Plasma cells localize within infected alveoli during recall responses

Memory B cells can quickly differentiate into PCs. We therefore asked whether the observed accumulation of lung BRM cells within newly infected regions is associated with changes in PC distribution. Consistent with our previous observations (Figure 2A), during the memory phase PCs were largely absent from the alveoli and were primarily confined to clusters near large airways (Figure 4A, left). In contrast, within 4 days of rechallenge, an additional population of PCs appeared within the parenchyma (Figure 4A, right). We refer to these cells as "alveolar PCs" to distinguish them from PCs within airway-associated clusters. Similar to activated BRM cells, alveolar PCs showed preferential accumulation in infected sites, as indicated by their higher density in these regions 4 days post rechallenge (Figures 4A–4C). Furthermore, at this time point, a significant reduction in the Ripley's K values of the total PC population was observed, reflecting a partial loss of the tightly "clustered" organization and a shift toward sparsely distributed cells (Figures 4D and 4E). To identify whether this population was unique to the anamnestic response, we analyzed PC positioning in mice during primary infection with influenza. No PCs were observed in the lungs in any site during the first 4 days postinfection. Within 20 days, PCs appeared in infected lungs, but they were confined to airway branch points, similar to their distribution during the memory phase (Figure S3D). This similarity was further reflected by the Ripley's K values of the cells which were comparable with those measured

Figure 2. Lung-resident memory B cells increase motility following rechallenge

(A) Snapshots from two-photon laser-scanning microscopy (TPLSM) of live explant lungs of BAT >6 weeks postinfection. Top, a typical iBALT-like structure (left), airway-associated PC clusters (middle), and BRM cells (right). Bottom, a BRM cell probing an alveolus. Time lapse is shown in minutes:seconds. White dotted line, alveolus boundaries. Data are representative of 9 videos.

(B) Top, experimental design. Bottom left, snapshots from live imaging 24 h post rechallenge or resting memory. Yellow, BRM cell migration tracks. Bottom right, plots displaying tracks of BRM cells from common origin.

(C) Time lapse of BRM cells migrating 24 h post rechallenge. Yellow line, migration path of a BRM cell.

(D) Mean velocities (left) and displacement (right) of BRM cells in lungs treated as indicated. Each dot represents one tracked cell.

(E) Frequency of BRM cell migration velocities. Data in (D and E) were pooled from 4 independent experiments with a total of 4–6 mice per group. Statistical analysis in (D) was made using Kruskal-Wallis tests. Error bars represent SD. $^{**}p < 0.01$; $^{***}p < 0.001$; $^{****}p < 0.0001$. See also Figure S2.

CellPress
OPEN ACCESS

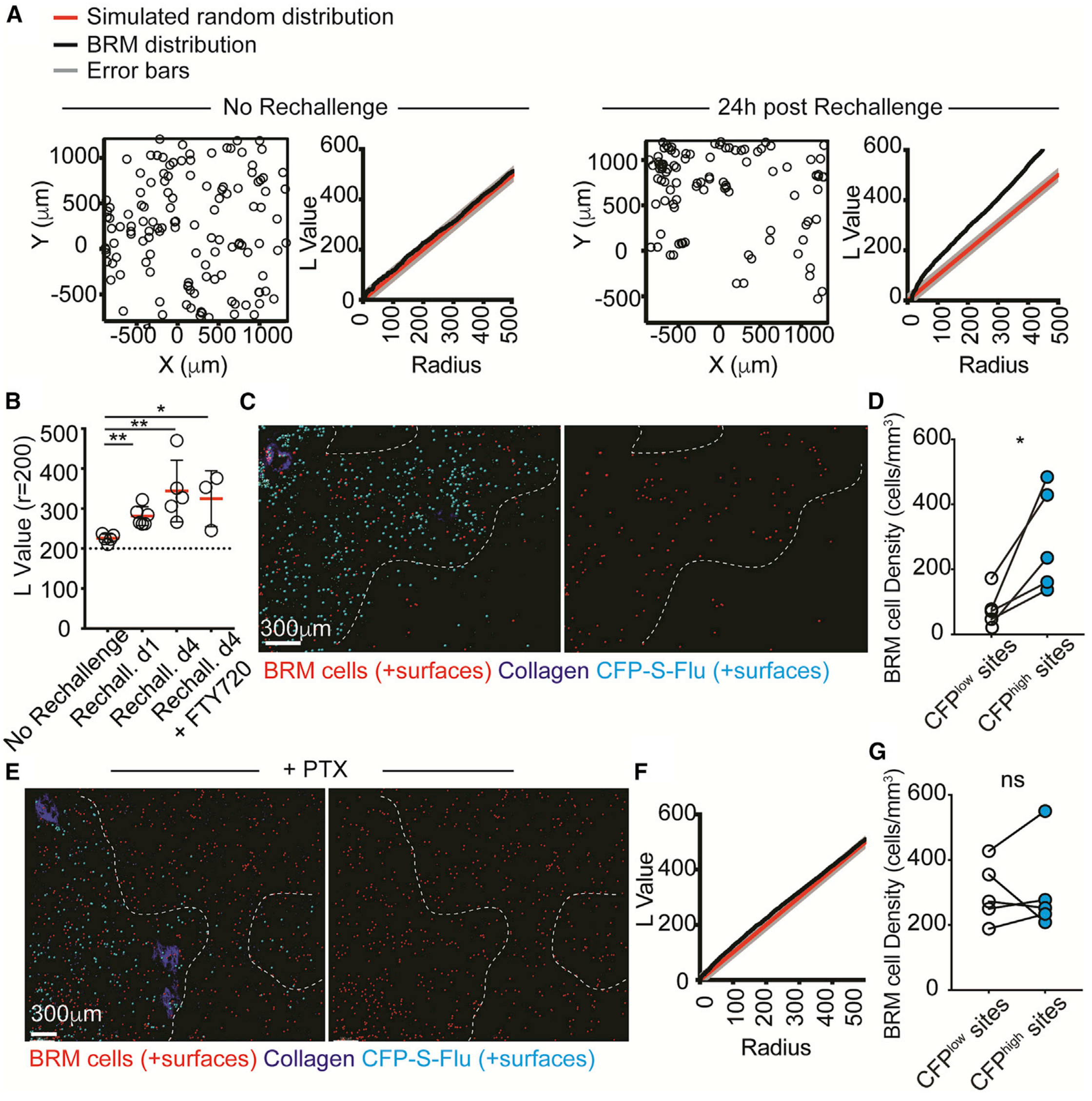

Figure 3. Memory B cells colocalize with infected cells early after activation

(A) Point patterns of BRM cell distribution before and after rechallenging. Plots display clustering L values of observed data versus complete spatial random simulation (simulation n = 1,000).

(B) L values at r = 200 of BRM cells. Data are pooled from 3–5 independent experiments per group. Each circle represents the mean L value calculated for one mouse (± SD), based on multiple images tiles collected from each mouse.

(C) BRM cells 24 h post rechallenge with CFP-S-Flu. Dotted lines demarcate border between highly infected and uninfected areas. Cells are highlighted using Imaris-created spots.

(D) BRM cell density in infected and uninfected sites 24 h after rechallenge. Each pair of points represents the average BRM cell density in one mouse, obtained by averaging data from multiple large tiles per animal.

(E) A representative image from mice treated with PTX 2 h after CFP-S-Flu rechallenge.

(F) L value plot of BRM cells in PTX-treated rechallenged mice.

(G) BRM cell density in infected and uninfected areas of PTX-treated, rechallenged lungs.

Data are pooled from 4 (C and D) or 3 (E and G) independent experiments. Statistical analyses were made using Mann-Whitney test (B) and paired t tests (D and G). Error bars represent SD. *p < 0.5; **p < 0.01. See also Figure S3.

during the memory phase (Figure 4E). Thus, the alveolar localization of PCs is unique to secondary responses, consistent with their development requiring the presence of pre-existing memory B cells. Moreover, treatment with FTY720 did not block alveolar PC differentiation, supporting the notion that they are derived from local cells (Figure 4E).

The above findings led us to hypothesize that, during secondary infections newly generated PCs primarily localize to infected alveoli, and while memory phase PCs remain largely confined to airway-associated clusters. To explore this possibility, we quantified the density of cluster-associated and alveolar PCs before and after reinfection. We found that while the concentration of

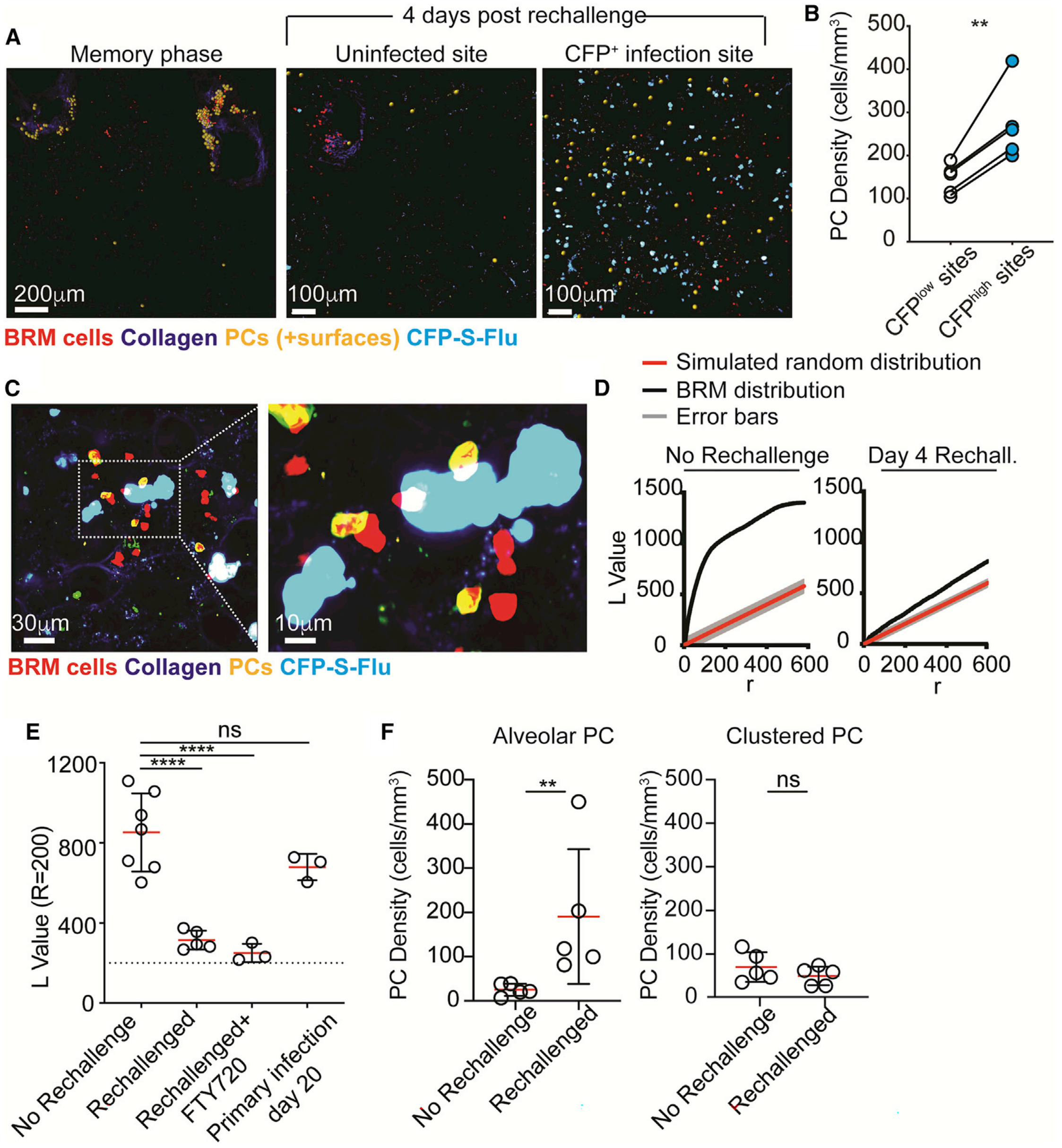

Figure 4. Plasma cells appear within infected alveoli within 4 days of rechallenge

(A) TPLSM images of infected and uninfected BAT mice 4 days post rechallenge with CFP-S-Flu. PCs are highlighted using Imaris-created spots (yellow).

(B) Alveolar PC density in infected or uninfected areas 4 days after rechallenge with CFP-S-Flu. Data are pooled from 4 independent experiments.

(C) Left, confocal microscopy of alveolar PCs near infected cells. Right, zoom of indicated boxed region.

(D) L values of observed PC positioning compared with spatial random simulation. Data show the results of one experiment out of 5 performed (at least one mouse per group per experiment).

(E) PC L values at r = 200.

(F) Densities of alveolar and clustered PCs. Each circle represents the mean densities measured in one mouse, obtained by averaging data from multiple large tiles per animal.

Data in (E) and (F) are pooled from 3–5 independent experiments. Statistical analyses were made using a paired t test (B), one-way ANOVA (E), and Mann-Whitney U test (F). Error bars represent SD. $^{**}p < 0.01$;$^{****}p < 0.0001$.

alveolar PCs increased by ~7-fold after rechallenge (Figure 4F, left), the density of PCs within clusters did not change significantly (Figure 4F, right). Of note, while BRM cells and lung PCs comprised IgM-, IgG-, and IgA-switched cells (Figure S3E; Allie et al., 2019; Oh et al., 2021), the frequencies of the IgG-switched fraction preferentially expanded during the initial wave of PC generation, potentially indicating a preferential differentiation of BRM cells with this isotype (Figures S3F and S3G).

Thus, we propose that during the early phase of the recall response, the main wave of newly generated PCs localizes to

CellPress
OPEN ACCESS

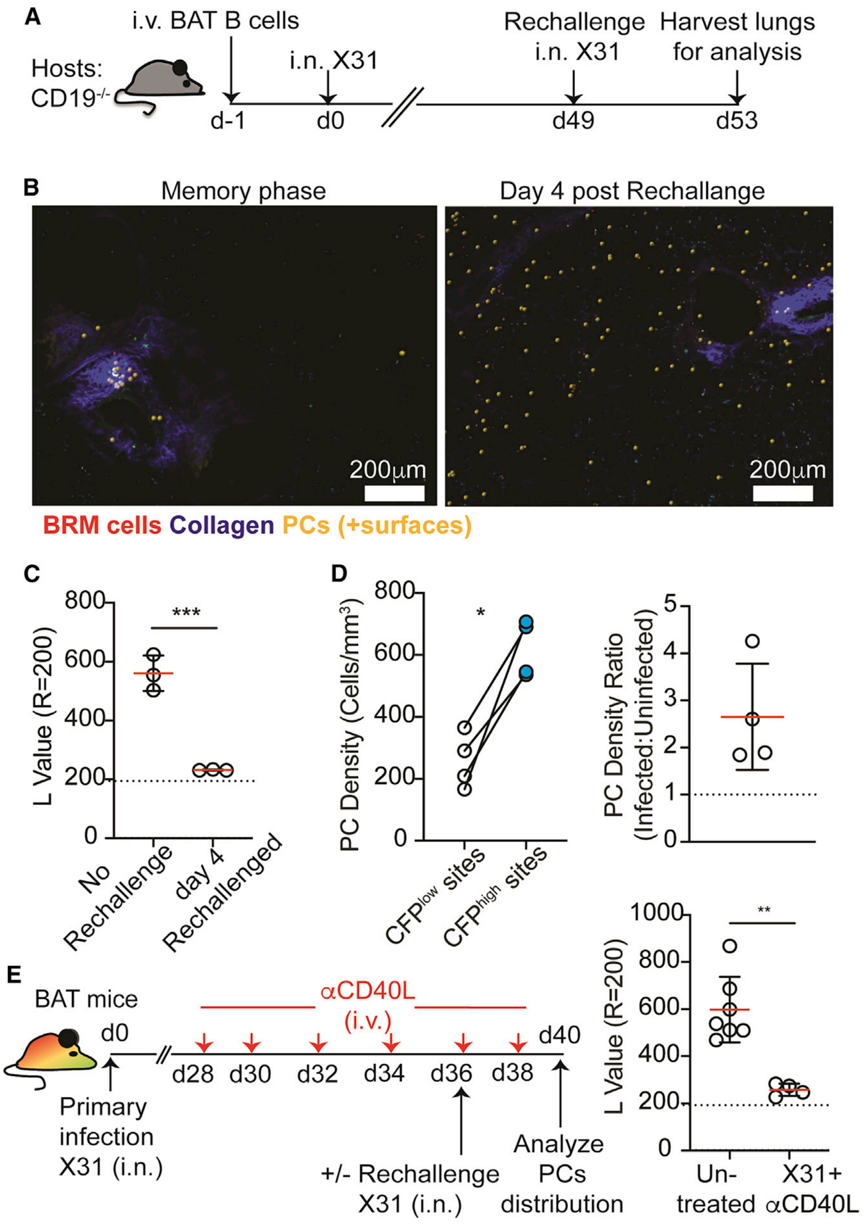

Figure 5. Alveolar plasma cells are derived from memory B cells

(A) Experimental outline for (A–C).

(B) TPLSM images of CD19$^{-/-}$ mice transferred with BAT B cells as described in (A) before and after rechallenge. PCs are highlighted using Imaris-created spots (yellow).

(C) PCs L values at r = 200.

(D) Alveolar PC density in infected or uninfected sites. Right, data represented as the fold-change difference.

(E) Left, experimental outline. Right, PCs L values in untreated resting memory mice or rechallenged mice treated with anti-CD40L.

Statistical analysis were made using an unpaired t test (C), a paired t test (D), and a Mann-Whitney U test (E). Error bars represent SD. *p < 0.05; **p < 0.01; ***p < 0.001. See also Figure S4.

infected alveoli. Given that PCs are remarkably efficient antibody factories capable of producing up to ~1,000 antibody molecules per second (Khodadadi et al., 2019), this process may represent a powerful mechanism that dramatically increases local antibody concentrations at sites of infection and thereby facilitate virus neutralization prior to systemic recall antibody responses increasing to the required degree.

Alveolar PCs develop from BRM cells independently of naive B cell input

Our findings so far favored the possibility that alveolar PCs are generated *in situ* from lung BRM cells. To further test this hypothesis and to formally exclude a requirement for input from newly activated naive B cells, we developed an adoptive transfer approach to follow memory B cell responses. For this, we injected CD19^{+} B cells into CD19$^{-/-}$ hosts, in which endogenous B cells fail to establish mature GCs (Carter and Myers, 2008). This strategy allowed us to reduce competition between transferred and endogenous B cells and to track the response of transferred polyclonal B cells to infection over time. As expected, CD19$^{-/-}$ hosts that were reconstituted with CD19^{+} B cells (derived from BAT) (Figure 5A) developed a population of lung BRM cells that persisted >70 days after primary infection and displayed characteristic alveolar surveillance behavior (Video S4), whereas PCs were distributed in clusters near large airways (Figure 5B, left). Consistent with our previous results, within 4 days of secondary infection, PCs appeared throughout the alveoli (Figure 5B, right) leading to reduced clustering values as measured by Ripley's K (Figure 5C). As before, these cells preferentially accumulated at infected sites (Figure 5D). Importantly, CD19$^{-/-}$ reconstituted mice that were infected with influenza 49 days earlier did not respond to secondary challenge with the irrelevant antigen sheep red blood cells (Figures S4A and S4B), confirming that transferred naive CD19^{+} B cells did not persist within these hosts, and consequently that the secondary influenza responses were indeed memory B cell dependent. We conclude that the development of alveolar PCs is independent of input from newly activated naive B cells.

Within the alveoli, PCs at days 1–4 postinfection were sessile. However, on rare occasions a few migratory PCs were detected near GC-like structures (Video S5). This raised the possibility that alveolar PCs may arise from local GCs prior to relocating to infected sites. To address this, we depleted pre-existing GC B cells prior to rechallenging the mice and assessed the effect on alveolar PC formation. BAT mice were infected with influenza and 28 days later were treated with anti-CD40L antibody every

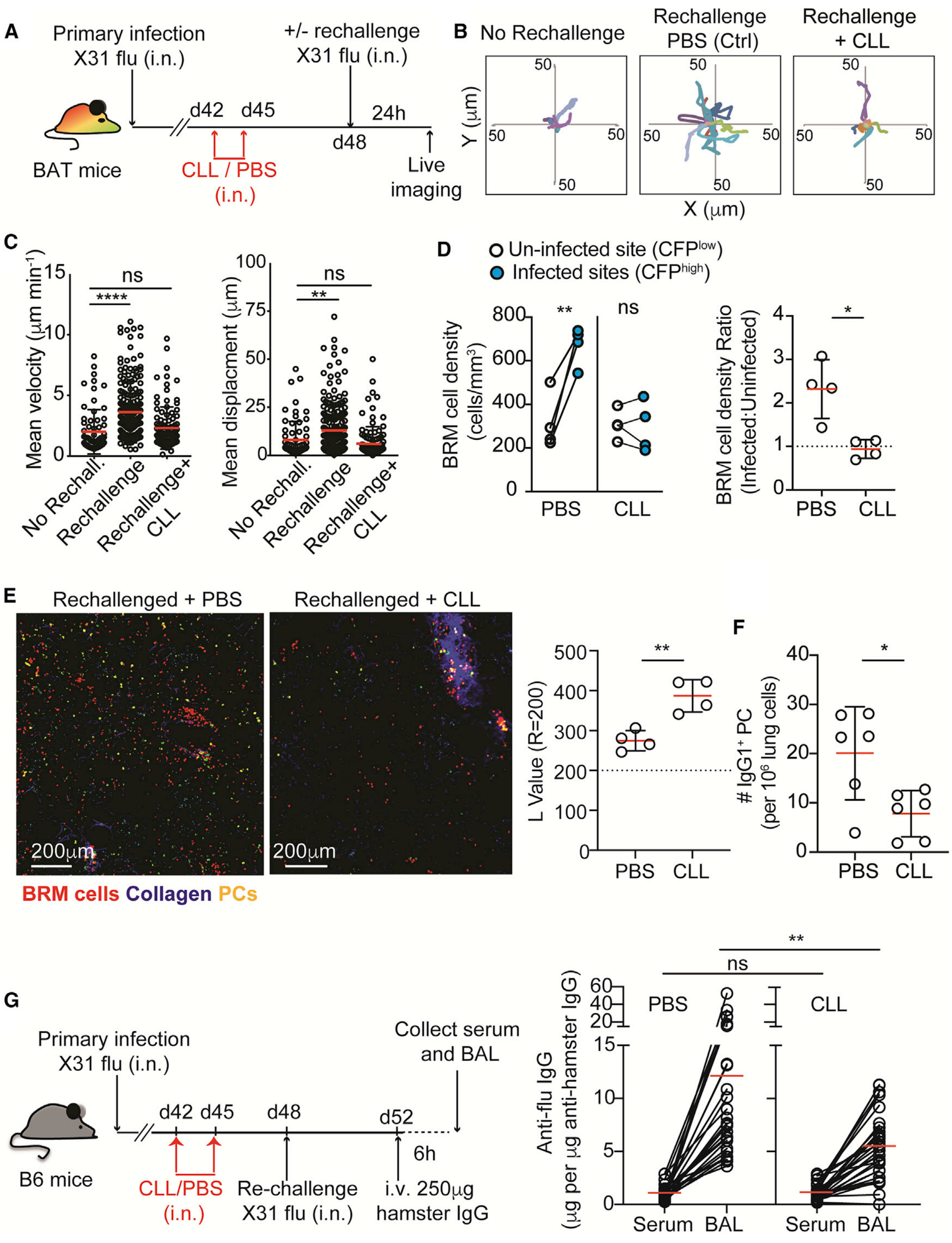

Figure 6. Depletion of alveolar macrophages leads to loss of resident memory B cell mobilization and plasma cell differentiation in infected lungs

(A) Experimental setup for (A–C).

(B) Plots of BRM cell tacks migrating from a common origin.

(C) Mean velocities and displacements of BRM cells. Data are pooled from 4 independent experiments with a total of 3–4 mice per group.

(D) BRM density in uninfected and infected sites of mice treated as in (A) and infected with CFP-S-Flu. Right, data represented as the fold-change difference. Data are pooled from 4 independent experiments.

(E) PCs 4 days post rechallenge in PBS- and CLL-treated mice. Left, representative images using TPLSM. Right, L values of PCs at r = 200. Plots are pooled from 4 independent experiments.

(legend continued on next page)

other day for 10 days (Figure 5E). As previously reported (Allie et al., 2019), this treatment led to loss of GC B cells in the lungs and other sites (Figure S4C). On day 36, half of the mice were subject to a viral rechallenge and their lung PC distribution was compared with that of nonrechallenged mice 4 days later (Figure 5E). Despite the absence of GC B cells, alveolar PCs were readily detected within these mice, and their Ripley's K L value dropped compared with the unchallenged animals (Figure 5E).

Taken together, these result show that while the development of alveolar PC depends on the presence of pre-existing memory B cells, reactivation of the GC reaction is dispensable for this process.

Alveolar macrophages orchestrate the localization and activation of lung BRM cells during secondary responses

Alveolar macrophages are located in the airway lumen, where they can interact with newly inhaled virions and infected cells. To test whether these cells are involved in regulating the recruitment of BRM to infected sites, we infected BAT mice with influenza and 6 weeks later injected clodronate-loaded liposomes (CLLs) intranasally. As previously reported (Leemans et al., 2001), this approach removed the majority of alveolar macrophages without depleting parenchymal phagocytes (Figure S5A). Control (PBS-loaded liposomes) or CLL-treated mice were rechallenged and BRM cell movement was monitored in live explant lung sections using two-photon microscopy (Figures 6A–6C). While BRM cells in the lungs of control mice demonstrated the expected increases in migration velocities and cell displacement after rechallenge, this effect was completely lost in animals depleted of alveolar macrophages (Figures 6B and 6C). Under these conditions, the distribution of alveolar BRM cells remained unchanged, with no detectable accumulation at infected sites (Figure 6D). This effect also correlated with a partial but significant impairment in the accumulation of PCs within alveolar regions, with very few PCs being detected outside of clusters in CLL-treated mice 4 days post rechallenge (Figure 6E, left). As expected, the L values for PCs in CLL-treated mice remained relatively high (Figure 6E, right). Consistent with these observations, we found that the total IgG1 PC numbers derived from the lungs of CLL versus control treated mice at day 4 post rechallenge were reduced (Figure 6F).

These above findings indicated that alveolar PC generation may be a mechanism to quickly increase antibody concentrations in the lung. However, we were conscious that antibodies are also produced by pre-existing long-lived PCs in other tissues/lung regions. To formally test whether alveolar PCs make a measurable contribution to local antibody concentrations, mice received intravenous injection of hamster IgG before the relative abundance of anti-influenza antibodies in the serum and bronchoalveolar lavage (BAL) of CLL-treated and control rechallenged mice was measured 4 days post rechallenge. Tracing transferred hamster IgG allowed us to quantify local versus transuded IgG from the serum (Alley et al., 1980; Burnett, 1986). Because hamster IgG concentrations plateaued in the lungs within 6 h of transfer (Figure S5B), subsequent measurements were made at this time point. The relative abundance of anti-influenza IgG and hamster IgG and in the BAL and serum were determined (Figure 6G). We detected an average increase of $\sim$12-fold in the ratio of anti-influenza IgG:hamster IgG in the BAL compared with the serum derived from the same animal, indicating that significant local production of anti-virus IgG occurs within the lung parenchyma. In contrast, in the CLL-treated animals, a more modest change was measured with an average increase of $\sim$5-fold in BAL over serum (Figure 6G).

These observations suggest that alveolar macrophages are likely necessary for recruitment of BRM cells to infected sites, a migratory step that correlates with rapid differentiation of PCs and localized increase in virus-specific antibodies within infected lungs.

CXCR3 mediates BRM cell accumulation in infected sites

We next aimed to define the mechanisms that facilitate BRM cell accumulation in sites of viral entry. Since alveolar macrophages die shortly after infection, we hypothesized that they act upstream to other leukocytes, which actively secrete chemotactic cues that attract BRM cells. To address this, we performed single-cell RNA-seq analysis on total lung leukocytes before and 1 day after rechallenge in control and CLL-treated mice. We aimed to identify inflammatory chemokines that are induced after rechallenge in an alveolar macrophage-dependent manner and which may attract BRM cells.

After quality control and removal of doublets and contaminating cells, 13,172 cells were retained for downstream analysis, comprising 4,387 control, 5,574 rechallenged, and 3,211 CLL-treated and rechallenged cells. Fourteen well-defined clusters were identified (Figures 7A, S6A, and S6B). As expected (Alon et al., 2021; Kulikauskaite and Wack, 2020), rechallenge induced marked increases in neutrophil, mono.Mac and NK cell numbers while alveolar macs, DC, $\gamma\delta$ T cells, and ILC2 cells were reduced in frequency (Figure S6C). Comparison of chemokine and cytokine gene expression in the CLL-treated and untreated rechallenged animals identified 10 genes with significantly lower expression following CLL treatment in at least one cell type (Figures 7B and 7C). Of those, the most significant reduction was observed in *Cxcl10* expression by neutrophils and mono.Mac. In addition, expression of *Cxcl9*, which similarly to CXCL10 activates CXCR3, was also significantly reduced in neutrophils. These results were confirmed by qPCR analysis (Figure 7D). Further analysis showed that interferon gamma (IFNγ), a potent inducer of *Cxcl10* and *Cxcl9*, was lower in NK and CD8 T cells

(F) Lung PCs analyzed by flow cytometry 4 days post rechallenge of mice treated with CLL or PBS liposomes as in (A). Data represent one of 3 independent experiments.

(G) Left, experimental design. Right, ELISA of anti-influenza (flu) ratios between concentrations of anti-influenza and hamster antibodies measured in the serum and BAL of PBS and CLL-treated rechallenged mice. Each circle represents one mouse. Data are pooled from 3 independent experiments performed. Lines indicate matched data from individual animals.

Statistical analysis were made using Kruskal-Wallis tests (C), a paired t test (D, left plot), unpaired t tests (E and F), and Mann-Whitney U test (D, right plot, and G). Error bars represent SD. *$p < 0.05$; **$p < 0.01$; ****$p < 0.0001$. See also Figure S5.

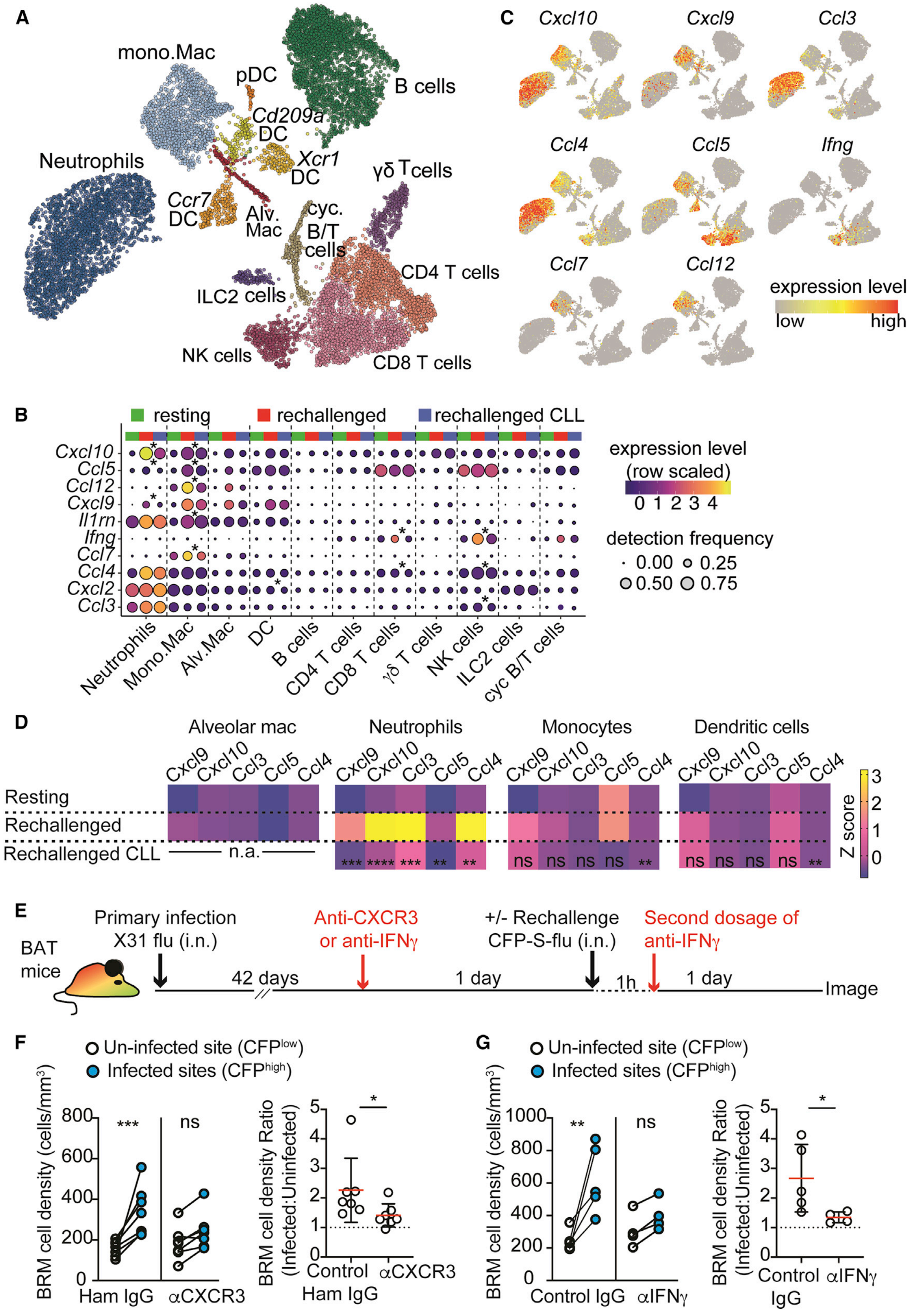

(legend on next page)

CellPress
OPEN ACCESS

in CLL rechallenged mice (Figures 7B and 7C), suggesting that alveolar macrophages may be necessary for optimal activation of this pathway. Gene-set-pathway analysis further supported this hypothesis, indicating that CLL treatment resulted in an impaired IFNγ response by myeloid cells (Figures S7A and S7B).

BRM cells express genes that encode several inflammatory GPCRs (Figure S7C) including CXCR3, which is activated by CXCL9 and CXCL10. While this receptor has been shown to be dispensable for recruitment of B cells into infected lungs (Denton et al., 2019), its impact on BRM cell localization within the tissue is unknown. To address this, we treated mice with CXCR3 blocking antibodies 1 day prior to rechallenge with CFP-S-Flu and determined the distribution of BRM cells in infected and uninfected areas, as before. Blocking CXCR3 led to a reduction in accumulation of BRM cells in infected sites, leading to a modest 1.2-fold increase of density in these regions after reinfection (compared with >2-fold increase in the control group) (Figures 7E and 7F). A similar effect was observed when IFNγ was neutralized (Figure 7G), consistent with it being a potent inducer of CXCL9 and CXCL10 expression (Groom and Luster, 2011; Luster et al., 1985). Thus, we conclude that activation of the chemokine receptor CXCR3 plays an important role in mobilizing BRM cells to sites of infection.

DISCUSSION

A hallmark of adaptive immunity is that antibody and T cell receptor repertoires are "educated" based on prior experiences, a process that involves selective expansion and long-term maintenance of antigen-experienced clones, or memory cells. However, in recent years, added layers of complexity have been revealed, and it has become clear that adaptive immune populations are capable of remembering not only the nature of the antigens they encountered but also the specific sites in which the infection is likely to occur, such that susceptible areas are reinforced with protective clones (Iwasaki, 2016; Masopust and Soerens, 2019; Mueller and Mackay, 2016). While this type of localized immunity has proven highly effective in the case of resident memory T cells, far less is known about the humoral components of the response.

Here, we explored the spatiotemporal regulation of BRM cells within live peripheral tissue. Using a reporter mouse to monitor BRM cells and PCs by two-photon microscopy, and by employing robust quantitative approaches to define dynamic changes in the distribution of these cells within infected lungs, we traced key steps that lead to local production of antibodies. We showed that prior to rechallenge, BRM cells were randomly distributed throughout the lungs where they appear to be in a relative state of rest while probing local alveoli. However, upon rechallenge, these cells quickly increased their migration capacity and relocated to sites of infection, where they differentiated into antibody-secreting PCs. Localization to sites of infection was independent of recruitment of recirculating B cells or antigen specificity but required the presence of alveolar macrophages and innate signals. Single-cell RNA-seq and QPCR analysis of rechallenged lungs revealed a likely role for alveolar macrophages in orchestrating this process, in part by promoting local production of IFNγ by infiltrating NK cells, subsequently leading to induction of inflammatory chemokines, including CXCL9 and CXCL10. This, in turn, promoted CXCR3-dependent accumulation of BRM cells within sites of infection where they quickly differentiated into PCs. Since alveolar macrophages are not major producers of CXCR3 ligands, we propose that they are necessary for triggering, rather than directly instructing, BRM cell responses in the lung. This hypothesis is compatible with previous demonstrations that alveolar macrophages are an important source of cytokines during influenza infection, but that this response lasts ~1 day, after which these cells die and are replaced by infiltrating monocytes (Aegerter et al., 2020; Kulikauskaite and Wack, 2020).

While our work establishes a major role for CXCR3 in regulating BRM mobilization and activation, other inflammatory chemokine receptors may also contribute to this effect. This possibility is supported by our observation that depletion of alveolar macrophages prevented BRM cell mobilization more effectively than blocking CXCR3. Moreover, we found that alveolar macrophage depletion was also associated with reduced expression of *Ccl4*, *Ccl5*, and *Ccl3*, transcripts encoding inflammatory chemokines that engage the chemotactic receptors CCR1 and CCR5, both of which are expressed in BRM cells. Expression of *Cxcl9*, and *Cxcl10* in rechallenged mice was most impacted in neutrophils and monocytes when alveolar macrophages were depleted, suggesting a possible contribution of these cell subsets to BRM cell accumulation within infected sites. Alternatively, it remains possible that other cell types, including nonhematopoietic populations that line the lung parenchyma and have not been included in our single-cell RNA analysis, contribute to this process. Additional work is needed to test these hypotheses and identify the cellular mechanisms that drive BRM cell recruitment to infected foci.

Figure 7. Migration of resident memory B cells to sites of infection is regulated by CXCR3

(A–C) scRNA-seq of lung leukocytes prior to rechallenge and 1 day post rechallenge of PBS or CLL-treated mice (treated as in Figure 6A). (A) UMAP showing clusters of total cells detected under all 3 conditions combined. (B) Dot plots showing the expression of chemokines and cytokines significantly downregulated in at least one cell type in the CLL versus PBS treated rechallenge mice. Genes in which most significant changes are observed are at the top (p ranges from $<10^{-23}$ to 10^{-3}). Significant differences are indicated by asterisks (Wilcoxon tests, >1.5× change, BH adjusted $p < 0.05$). (C) UMAPs showing the expression of selected chemokines and cytokines of interest under all conditions combined.

(D) Heatmap of qPCR data from the indicated cell subsets sorted from mice treated as in (A), showing results of one of two independent experiments. The average expression from 3 biological replicates is shown. Statistical symbols indicate results of one-way ANOVA, comparison between "rechallenged CLL" and "rechallenged" groups.

(E) Experimental setup for (E–G). Animals received one of the two blocking treatments options shown.

(F and G) BRM cell density (left), and fold difference of BRM cell densities between infected and uninfected areas (right), in rechallenged mice treated with anti-CXCR3 (F), or anti-IFNγ (G). Data in (F) and (G) are pooled from 4–7 independent experiments. Statistical analysis (F and G) was done using a paired t test (left plots) and Mann-Whitney U tests (right plots). Error bars represent SD. *$p < 0.05$; **$p < 0.01$; ***$p < 0.001$; ****$p < 0.0001$.

See also Figures S6 and S7.

An important advantage of our approach is that it allowed us to simultaneously visualize BRM cells and PCs. We find that within 2–3 weeks of primary infection, PCs were detected in the lungs. At this early time point, the cells were largely confined to airway-associated clusters ideally positioned to secrete antibodies into the BAL. This distribution remained unchanged throughout the memory phase. Given that the number of PCs within the lung was stable for up to 200 days postinfection, it is likely that these PC clusters contain long-lived cells that provide continuous protection through antibody secretion. However, this initial layer of protection may not be sufficient to prevent all infections. Under these conditions, BRM cells were locally reactivated and rapidly differentiated into PCs, which associated directly with infected alveoli. This result uncovered a previously unappreciated and important feature of BRM cells: the ability to deliver antibodies in a highly localized manner to sites of viral replication. PCs possess substantial secretory capacity, producing up to ~1,000 antibodies per second (Khodadadi et al., 2019), and therefore the presence of even just a few cells at sites of high viral load may provide substantial protection by inhibiting or slowing viral spread. Such a strategy may be particularly relevant for defense against pathogens such as influenza virus that are limited to one organ due to tissue tropism and for which systemic distribution of antibodies may therefore not provide a significant advantage.

Several lines of evidence support the notion that alveolar PCs differentiate directly from lung BRM cells. In our mouse model, expression of tdTomato indicates either previous or active upregulation of AID, a hallmark of B cell activation. Our finding that tdTomato^{+} mVenus^{+} alveolar PCs developed in the absence of ongoing GC responses suggests that tdTomato expression in these cells was induced during earlier events, i.e., at the time of the primary infection when humoral memory was being established. Moreover, while primary infection led to the development of airway-associated PCs, alveolar PCs were only detected after rechallenge, further supporting their arising from memory, rather than naive, newly activated B cells. This conclusion is further reinforced by our adoptive transfer experiments of B cells into CD19$^{-/-}$ hosts. In this system, naive transferred B cells did not survive for prolonged periods of time after infection, as indicated by their inability to respond to immunization with a new antigen. Yet, when the mice were reinfected with the same pathogen, the differentiation of donor-derived alveolar PCs was intact, demonstrating their memory-derived origin. Finally, we showed that in all the settings above, the development of alveolar PCs could come from a local source without the recruitment of circulating B cells, as indicated by the fact that FTY720 treatment had no effect on BRM cell movement or alveolar PC development. Together, these results support the notion that alveolar PCs can arise from pre-existing memory B cells that reside in the lung.

While our study uncovers a local mechanism to expedite the recruitment of local memory B cells directly to infected regions, this does not exclude the possibility that memory B cells recruited from the blood also contribute to the generation of alveolar PCs. In line with this possibility, recirculating memory B cells were shown to use CXCR3 to localize to the female reproductive tract of mice infected with genital herpes virus, where they subsequently differentiate into PCs (Oh et al., 2019). Notably however, in this tissue, neither BRM cells nor long-lived PCs develop. Instead, increases in local antibody concentrations during rechallenge entirely depend on recruitment of B cells from the circulation. It is possible that the development of BRM cells evolved specifically in the lung to provide enhanced protection from seasonally circulating airborne pathogens that are likely to be encountered again over the course of several months. Alternatively, BRM cells may represent a broader phenomenon that can be induced in other mucosal barriers, with some tissues being restrictive.

Most current vaccines aiming to elicit anti-influenza immunity are delivered peripherally in the hope that sufficiently high concentrations of neutralizing antibodies are induced to penetrate the lung and confer protection. In recent years, significant progress in understanding the 3D nature of how broadly neutralizing antibodies bind conserved influenza virus epitopes has led to substantial progress in the field, leading to the development of immunizing antigens that mimic stable structures and drive antibody responses against them (Corti et al., 2017; Krammer, 2019; Neu et al., 2016; Wrammert et al., 2008; Wu and Wilson, 2018). Yet, a major limitation to this approach remains that the titers generated through immunization are often too low to prevent infection (Coughlan and Palese, 2018). It is therefore highly plausible that combining strategies that induce cross-reactive antibodies with approaches that increase their concentrations near or at sites of viral entry will be beneficial (Iwasaki, 2016). Our study shows that natural mechanisms that support secretion of antibodies in a highly localized manner evolved in the lung and provides insights into the underlying pathways that facilitate this process. We anticipate that, in the near future, more studies exploring the factors that promote BRM cell retention and maintenance in the lung will help to exploit this mechanism for the development of better vaccines.

Limitations of the study

We have shown that BRM cells accumulate in infected sites and that CXCR3 was a major chemokine receptor that contributed to this process. However, whether CXCR3 ligands act either through directional chemotaxis or by generally increasing motility and thereby facilitating access to retention factors at infected sites, was not clear from our imaging studies because we did not see direct evidence of directional movement of cohorts of cells. This may reflect inherent limitations of relatively short imaging periods focused on small areas, where capturing synchronized cell movement is challenging. Additionally, while we found that depletion of alveolar macrophages led to impaired BRM cell activation, we have not established a direct role for these cells in regulating these events. Development of selective approaches to genetically target alveolar macrophages, and comprehensive analysis of the changes that are induced within these cells during the early phase of secondary infection, are needed to identify the roles and mechanisms by which they orchestrate BRM cell responses. Finally, more work is needed to definitively identify the key cellular sources of the chemotactic ligands that facilitate BRM cell positioning within infected regions. It should be noted that because our single-cell RNA-seq analysis focused on changes that occurred in leukocytes 24 h post rechallenge, the contribution of nonhematopoietic cells could not be investigated and the potential upregulation of chemotactic ligands at earlier

time points (e.g., prior to alveolar macrophage decline), was not evaluated.

STAR★METHODS

Detailed methods are provided in the online version of this paper and include the following:

- KEY RESOURCES TABLE
- RESOURCE AVAILABILITY
  - Lead contact
  - Materials availability
  - Data and code availability
- EXPERIMENTAL MODEL AND SUBJECT DETAILS
  - Mice
  - Viruses
- METHOD DETAILS
  - Influenza infection, rechallenge with VLPs and immunization
  - Influenza hemagglutinin (HA) production and biotinylation
  - VLP production
  - Generation of CFP-S-Flu
  - Flow cytometry, *in vivo* labeling, and cell sorting
  - Bulk and single cell RNA-seq analysis
  - Quantitative PCR
  - Adoptive cell transfer
  - Live explant imaging
  - Imaging of static lung sections using TPLSM
  - Immunofluorescence staining and confocal microscopy
  - Image acquisition and analysis
  - FTY720 administration
  - Cell depletion, antibody treatments and PTX administration
  - ELISA
- QUANTIFICATION AND STATISTICAL ANALYSIS

SUPPLEMENTAL INFORMATION

Supplemental information can be found online at https://doi.org/10.1016/j.immuni.2022.03.003.

ACKNOWLEDGMENTS

We thank M. Saitou and E. Robertson for the Blimp1^{mVenus} mice, N. Ashley and E. Repapi (WIMM single cell and CBRG facilities) for RNA-seq library generation and analysis assistance, and C. Lagerholm (Wolfson Imaging Centre Oxford) for imaging assistance. We also thank Anne Chauveau for critical reading of the manuscript. This work was supported by a Wellcome Trust Investigator Award (106245/Z/14/Z) and a Kennedy Trust Senior Fellowship (T.I.A.), Kennedy Trust Studentship Prizes (A.M.), the Clarendon Fund Scholarship (N.R.), and Wellcome Henry Dale/Senior Fellowships (105654/Z/14/Z, 220219/Z/20/Z) (O.B.), and by the Medical Research Council (MRC) through core funding to the MRC Human Immunology Unit and MRC WIMM.

AUTHOR CONTRIBUTIONS

A.J.M. provided conceptual contributions and designed, performed, and analyzed the majority of the experiments in the manuscript, including all of the live-imaging work and analysis of thick vibratome cut sections. N.R. provided conceptual contribution and designed, performed, and analyzed (non-live-imaging) experiments, including some sequencing analysis. M.F.B., A.E.-T., and A.C.G. provided expertise and reagents to generate and validate the quality of VLPs used for immunization. A.T., P.R., and T.K.T. provided expertise for viral assays, generating influenza reagents and engineering fluorescently labeled viral strains. E.E.T. provided expertise in establishing the live and static imaging procedures of lungs. M.A. performed the cell capture and generated the libraries for the scRNA-seq experiments. L.K. performed exploratory scRNA-seq analysis. C.A.P.M. analyzed the CITE-seq data. S.N.S. performed the final scRNA-seq data analysis, prepared the related figures and supervised the single-cell work. A.J.M. prepared most of the main and supplementary figures and all the videos, and T.I.A. wrote it with input from A.J.M., N.R., S.N.S., and O.B. T.I.A. and O.B. conceived the project and T.I.A supervised it.

DECLARATION OF INTERESTS

The authors declare no competing interests.

Received: September 9, 2021
Revised: January 25, 2022
Accepted: March 7, 2022
Published: March 28, 2022

REFERENCES

Adachi, Y., Onodera, T., Yamada, Y., Daio, R., Tsuiji, M., Inoue, T., Kobayashi, K., Kurosaki, T., Ato, M., and Takahashi, Y. (2015). Distinct germinal center selection at local sites shapes memory B cell response to viral escape. J. Exp. Med. *212*, 1709–1723.

Aegerter, H., Kulikauskaite, J., Crotta, S., Patel, H., Kelly, G., Hessel, E.M., Mack, M., Beinke, S., and Wack, A. (2020). Influenza-induced monocyte-derived alveolar macrophages confer prolonged antibacterial protection. Nat. Immunol. *21*, 145–157.

Aibar, S., González-Blas, C.B., Moerman, T., Huynh-Thu, V.A., Imrichova, H., Hulselmans, G., Rambow, F., Marine, J.C., Geurts, P., Aerts, J., et al. (2017). SCENIC: single-cell regulatory network inference and clustering. Nat. Methods *14*, 1083–1086.

Allen, C.D., Okada, T., Tang, H.L., and Cyster, J.G. (2007). Imaging of germinal center selection events during affinity maturation. Science *315*, 528–531.

Alley, M.R., Wells, P.W., Smith, W.D., and Gardiner, A.C. (1980). The distribution of immunoglobulin in the respiratory tract of sheep. Vet. Pathol. *17*, 372–380.

Allie, S.R., Bradley, J.E., Mudunuru, U., Schultz, M.D., Graf, B.A., Lund, F.E., and Randall, T.D. (2019). The establishment of resident memory B cells in the lung requires local antigen encounter. Nat. Immunol. *20*, 97–108.

Allie, S.R., and Randall, T.D. (2020). Resident memory B cells. Viral Immunol. *33*, 282–293.

Alon, R., Sportiello, M., Kozlovski, S., Kumar, A., Reilly, E.C., Zarbock, A., Garbi, N., and Topham, D.J. (2021). Leukocyte trafficking to the lungs and beyond: lessons from influenza for COVID-19. Nat. Rev. Immunol. *21*, 49–64.

Bachmann, M.F., and Jennings, G.T. (2010). Vaccine delivery: a matter of size, geometry, kinetics and molecular patterns. Nat. Rev. Immunol. *10*, 787–796.

Bannard, O., and Cyster, J.G. (2017). Germinal centers: programmed for affinity maturation and antibody diversification. Curr. Opin. Immunol. *45*, 21–30.

Barker, K.A., Etesami, N.S., Shenoy, A.T., Arafa, E.I., Lyon de Ana, C., Smith, N.M., Martin, I.M., Goltry, W.N., Barron, A.M., Browning, J.L., et al. (2021). Lung-resident memory B cells protect against bacterial pneumonia. J. Clin. Invest. *131*, e141810.

Bergen, V., Lange, M., Peidli, S., Wolf, F.A., and Theis, F.J. (2020). Generalizing RNA velocity to transient cell states through dynamical modeling. Nat. Biotechnol. *38*, 1408–1414.

Burnett, D. (1986). Immunoglobulins in the lung. Thorax *41*, 337–344.

Carter, R.H., and Myers, R. (2008). Germinal center structure and function: lessons from CD19. Semin. Immunol. *20*, 43–48.

Chiu, C., Ellebedy, A.H., Wrammert, J., and Ahmed, R. (2015). B cell responses to influenza infection and vaccination. Curr. Top. Microbiol. Immunol. *386*, 381–398.

COVID-19 Multi-omics Blood Atlas (COMBAT) Consortium, Ahern, D.J., Ai, Z., Ainsworth, M., Allan, C., Allcock, A., Ansari, A., Arancibia-Carcamo, C.V., Aschenbrenner, D., Attar, M., et al. (2021). A blood atlas of COVID-19 defines hallmarks of disease severity and specificity. Preprint at medRxiv. https://doi.org/10.1101/2021.05.11.21256877.

Cornelis, R., Chang, H.D., and Radbruch, A. (2021). Keeping up with the stress of antibody production: BAFF and APRIL maintain memory plasma cells. Curr. Opin. Immunol. *71*, 97–102.

Corti, D., Cameroni, E., Guarino, B., Kallewaard, N.L., Zhu, Q., and Lanzavecchia, A. (2017). Tackling influenza with broadly neutralizing antibodies. Curr. Opin. Virol. *24*, 60–69.

Coughlan, L., and Palese, P. (2018). Overcoming barriers in the path to a universal influenza virus vaccine. Cell Host Microbe *24*, 18–24.

Cyster, J.G., and Schwab, S.R. (2012). Sphingosine-1-phosphate and lymphocyte egress from lymphoid organs. Annu. Rev. Immunol. *30*, 69–94.

Denton, A.E., Innocentin, S., Carr, E.J., Bradford, B.M., Lafouresse, F., Mabbott, N.A., Mörbe, U., Ludewig, B., Groom, J.R., Good-Jacobson, K.L., and Linterman, M.A. (2019). Type I interferon induces CXCL13 to support ectopic germinal center formation. J. Exp. Med. *216*, 621–637.

Dobin, A., Davis, C.A., Schlesinger, F., Drenkow, J., Zaleski, C., Jha, S., Batut, P., Chaisson, M., and Gingeras, T.R. (2013). STAR: ultrafast universal RNA-seq aligner. Bioinformatics *29*, 15–21.

Fooksman, D.R., Schwickert, T.A., Victora, G.D., Dustin, M.L., Nussenzweig, M.C., and Skokos, D. (2010). Development and migration of plasma cells in the mouse lymph node. Immunity *33*, 118–127.

Frank, G.M., Angeletti, D., Ince, W.L., Gibbs, J.S., Khurana, S., Wheatley, A.K., Max, E.E., McDermott, A.B., Golding, H., Stevens, J., et al. (2015). A simple flow-cytometric method measuring B cell surface immunoglobulin avidity enables characterization of affinity maturation to influenza A virus. mBio *6*, e01156.

Gomes, A.C., Flace, A., Saudan, P., Zabel, F., Cabral-Miranda, G., Turabi, A.E., Manolova, V., and Bachmann, M.F. (2017). Adjusted particle size eliminates the need of linkage of antigen and adjuvants for appropriated T cell responses in virus-like particle-based vaccines. Front. Immunol. *8*, 226.

Good-Jacobson, K.L., and Tarlinton, D.M. (2012). Multiple routes to B-cell memory. Int. Immunol. *24*, 403–408.

Groom, J.R., and Luster, A.D. (2011). CXCR3 ligands: redundant, collaborative and antagonistic functions. Immunol. Cell Biol. *89*, 207–215.

Hao, Y., Hao, S., Andersen-Nissen, E., Mauck, W.M., 3rd, Zheng, S., Butler, A., Lee, M.J., Wilk, A.J., Darby, C., Zager, M., et al. (2021). Integrated analysis of multimodal single-cell data. Cell *184*, 3573–3587.e29.

Hauser, A.E., Junt, T., Mempel, T.R., Sneddon, M.W., Kleinstein, S.H., Henrickson, S.E., von Andrian, U.H., Shlomchik, M.J., and Haberman, A.M. (2007). Definition of germinal-center B cell migration *in vivo* reveals predominant intrazonal circulation patterns. Immunity *26*, 655–667.

Iwasaki, A. (2016). Exploiting mucosal immunity for antiviral vaccines. Annu. Rev. Immunol. *34*, 575–608.

Jafari-Mamaghani, M., Andersson, M., and Krieger, P. (2010). Spatial point pattern analysis of neurons using Ripley's K-function in 3D. Front. Neuroinform. *4*, 9.

Joo, H.M., He, Y., and Sangster, M.Y. (2008). Broad dispersion and lung localization of virus-specific memory B cells induced by influenza pneumonia. Proc. Natl. Acad. Sci. USA *105*, 3485–3490.

Khodadadi, L., Cheng, Q., Radbruch, A., and Hiepe, F. (2019). The maintenance of memory plasma cells. Front. Immunol. *10*, 721.

Kiskowski, M.A., Hancock, J.F., and Kenworthy, A.K. (2009). On the use of Ripley's K-function and its derivatives to analyze domain size. Biophys. J. *97*, 1095–1103.

Korsunsky, I., Millard, N., Fan, J., Slowikowski, K., Zhang, F., Wei, K., Baglaenko, Y., Brenner, M., Loh, P.R., and Raychaudhuri, S. (2019). Fast, sensitive and accurate integration of single-cell data with Harmony. Nat. Methods *16*, 1289–1296.

Kozlovska, T.M., Cielens, I., Dreilinņa, D., Dislers, A., Baumanis, V., Ose, V., and Pumpens, P. (1993). Recombinant RNA phage Q beta capsid particles synthesized and self-assembled in *Escherichia coli*. Gene *137*, 133–137.

Krammer, F. (2019). The human antibody response to influenza A virus infection and vaccination. Nat. Rev. Immunol. *19*, 383–397.

Kulikauskaite, J., and Wack, A. (2020). Teaching old dogs new tricks? The plasticity of lung alveolar macrophage subsets. Trends Immunol. *41*, 864–877.

Leemans, J.C., Juffermans, N.P., Florquin, S., van Rooijen, N., Vervoordeldonk, M.J., Verbon, A., van Deventer, S.J., and van der Poll, T. (2001). Depletion of alveolar macrophages exerts protective effects in pulmonary tuberculosis in mice. J. Immunol. *166*, 4604–4611.

Lelkes, E., Headley, M.B., Thornton, E.E., Looney, M.R., and Krummel, M.F. (2014). The spatiotemporal cellular dynamics of lung immunity. Trends Immunol. *35*, 379–386.

Liao, Y., Smyth, G.K., and Shi, W. (2013). The Subread aligner: fast, accurate and scalable read mapping by seed-and-vote. Nucleic acids research *41* (10), e108. https://doi.org/10.1093/nar/gkt214.

Liao, Y., Smyth, G.K., and Shi, W. (2014). featureCounts: an efficient general purpose program for assigning sequence reads to genomic features. Bioinformatics *30*, 923–930.

Lun, A.T.L., Riesenfeld, S., Andrews, T., Dao, T.P., Gomes, T., and Marioni, J.C.; Participants in the 1st Human Cell Atlas Jamboree (2019). EmptyDrops: distinguishing cells from empty droplets in droplet-based single-cell RNA sequencing data. Genome Biol. *20*, 63.

Luster, A.D., Unkeless, J.C., and Ravetch, J.V. (1985). Gamma-interferon transcriptionally regulates an early-response gene containing homology to platelet proteins. Nature *315*, 672–676.

Masopust, D., and Soerens, A.G. (2019). Tissue-resident T cells and other resident leukocytes. Annu. Rev. Immunol. *37*, 521–546.

Matloubian, M., Lo, C.G., Cinamon, G., Lesneski, M.J., Xu, Y., Brinkmann, V., Allende, M.L., Proia, R.L., and Cyster, J.G. (2004). Lymphocyte egress from thymus and peripheral lymphoid organs is dependent on S1P receptor 1. Nature *427*, 355–360.

McCarthy, D.J., Campbell, K.R., Lun, A.T., and Wills, Q.F. (2017). Scater: pre-processing, quality control, normalization and visualization of single-cell RNA-seq data in R. Bioinformatics *33*, 1179–1186.

Mueller, S.N., and Mackay, L.K. (2016). Tissue-resident memory T cells: local specialists in immune defence. Nat. Rev. Immunol. *16*, 79–89.

Neu, K.E., Henry Dunand, C.J., and Wilson, P.C. (2016). Heads, stalks and everything else: how can antibodies eradicate influenza as a human disease? Curr. Opin. Immunol. *42*, 48–55.

Oh, J.E., Iijima, N., Song, E., Lu, P., Klein, J., Jiang, R., Kleinstein, S.H., and Iwasaki, A. (2019). Migrant memory B cells secrete luminal antibody in the vagina. Nature *571*, 122–126.

Oh, J.E., Song, E., Moriyama, M., Wong, P., Zhang, S., Jiang, R., Strohmeier, S., Kleinstein, S.H., Krammer, F., and Iwasaki, A. (2021). Intranasal priming induces local lung-resident B cell populations that secrete protective mucosal antiviral IgA. Sci. Immunol. *6*, eabj5129.

Ohinata, Y., Sano, M., Shigeta, M., Yamanaka, K., and Saitou, M. (2008). A comprehensive, non-invasive visualization of primordial germ cell development in mice by the Prdm1-mVenus and Dppa3-ECFP double transgenic reporter. Reproduction *136*, 503–514.

Onodera, T., Takahashi, Y., Yokoi, Y., Ato, M., Kodama, Y., Hachimura, S., Kurosaki, T., and Kobayashi, K. (2012). Memory B cells in the lung participate in protective humoral immune responses to pulmonary influenza virus reinfection. Proc. Natl. Acad. Sci. USA *109*, 2485–2490.

Phan, T.G., and Tangye, S.G. (2017). Memory B cells: total recall. Curr. Opin. Immunol. *45*, 132–140.

Powell, T.J., Rijal, P., McEwen-Smith, R.M., Byun, H., Hardwick, M., Schimanski, L.M., Huang, K.A., Daniels, R.S., and Townsend, A.R.M. (2019). A single cycle influenza virus coated in H7 haemagglutinin generates

CellPress
OPEN ACCESS

neutralizing antibody responses to haemagglutinin and neuraminidase glycoproteins and protection from heterotypic challenge. J. Gen. Virol. *100*, 431–445.

Powell, T.J., Silk, J.D., Sharps, J., Fodor, E., and Townsend, A.R. (2012). Pseudotyped influenza A virus as a vaccine for the induction of heterotypic immunity. J. Virol. *86*, 13397–13406.

Radtke, D., and Bannard, O. (2018). Expression of the plasma cell transcriptional regulator Blimp-1 by dark zone germinal center B cells During periods of proliferation. Front. Immunol. *9*, 3106.

Rickert, R.C., Rajewsky, K., and Roes, J. (1995). Impairment of T-cell-dependent B-cell responses and B-1 cell development in CD19-deficient mice. Nature *376*, 352–355.

Ripley, B.D. (1977). Modelling spatial patterns. J. R. Stat. Soc. B Methodol. *39*, 172–192.

Robinson, M.D., McCarthy, D.J., and Smyth, G.K. (2010). edgeR: a Bioconductor package for differential expression analysis of digital gene expression data. Bioinformatics *26*, 139–140.

Roco, J.A., Mesin, L., Binder, S.C., Nefzger, C., Gonzalez-Figueroa, P., Canete, P.F., Ellyard, J., Shen, Q., Robert, P.A., Cappello, J., et al. (2019). Class-switch recombination occurs infrequently in germinal centers. Immunity *51*, 337–350.e7.

Rommel, P.C., Bosque, D., Gitlin, A.D., Croft, G.F., Heintz, N., Casellas, R., Nussenzweig, M.C., Kriaucionis, S., and Robbiani, D.F. (2013). Fate mapping for activation-induced cytidine deaminase (AID) marks non-lymphoid cells during mouse development. PLoS One *8*, e69208.

Sallusto, F., Lanzavecchia, A., Araki, K., and Ahmed, R. (2010). From vaccines to memory and back. Immunity *33*, 451–463.

Saunders-Hastings, P.R., and Krewski, D. (2016). Reviewing the history of pandemic influenza: understanding patterns of emergence and transmission. Pathogens *5*, 66.

Schwickert, T.A., Lindquist, R.L., Shakhar, G., Livshits, G., Skokos, D., Kosco-Vilbois, M.H., Dustin, M.L., and Nussenzweig, M.C. (2007). *In vivo* imaging of germinal centres reveals a dynamic open structure. Nature *446*, 83–87.

Shlomchik, M.J., and Weisel, F. (2012). Germinal center selection and the development of memory B and plasma cells. Immunol. Rev. *247*, 52–63.

Stoeckius, M., Hafemeister, C., Stephenson, W., Houck-Loomis, B., Chattopadhyay, P.K., Swerdlow, H., Satija, R., and Smibert, P. (2017). Simultaneous epitope and transcriptome measurement in single cells. Nat. Methods *14*, 865–868.

Suan, D., Sundling, C., and Brink, R. (2017). Plasma cell and memory B cell differentiation from the germinal center. Curr. Opin. Immunol. *45*, 97–102.

Thornton, E.E., Looney, M.R., Bose, O., Sen, D., Sheppard, D., Locksley, R., Huang, X., and Krummel, M.F. (2012). Spatiotemporally separated antigen uptake by alveolar dendritic cells and airway presentation to T cells in the lung. J. Exp. Med. *209*, 1183–1199.

Ulbricht, C., Niesner, R.A., and Hauser, A.E. (2021). Imaging of bone marrow plasma cells and of their niches. Methods Mol. Biol. *2308*, 163–176.

Victora, G.D., and Wilson, P.C. (2015). Germinal center selection and the antibody response to influenza. Cell *163*, 545–548.

Weltzin, R., and Monath, T.P. (1999). Intranasal antibody prophylaxis for protection against viral disease. Clin. Microbiol. Rev. *12*, 383–393.

Wolf, F.A., Angerer, P., and Theis, F.J. (2018). SCANPY: large-scale single-cell gene expression data analysis. Genome Biol. *19*, 15.

Wolock, S.L., Lopez, R., and Klein, A.M. (2019). Scrublet: computational identification of cell doublets in single-cell transcriptomic data. Cell Syst. *8*, 281–291.e9.

Wrammert, J., Smith, K., Miller, J., Langley, W.A., Kokko, K., Larsen, C., Zheng, N.Y., Mays, I., Garman, L., Helms, C., et al. (2008). Rapid cloning of high-affinity human monoclonal antibodies against influenza virus. Nature *453*, 667–671.

Wu, N.C., and Wilson, I.A. (2018). Structural insights into the design of novel anti-influenza therapies. Nat. Struct. Mol. Biol. *25*, 115–121.

Xin, H., Lian, Q., Jiang, Y., Luo, J., Wang, X., Erb, C., Xu, Z., Zhang, X., Heidrich-O'Hare, E., Yan, Q., et al. (2020). GMM-Demux: sample demultiplexing, multiplet detection, experiment planning, and novel cell-type verification in single cell sequencing. Genome Biol. *21*, 188.

Young, M.D., Wakefield, M.J., Smyth, G.K., and Oshlack, A. (2010). Gene ontology analysis for RNA-seq: accounting for selection bias. Genome Biol. *11*, R14.

Zehentmeier, S., Roth, K., Cseresnyes, Z., Sercan, Ö., Horn, K., Niesner, R.A., Chang, H.D., Radbruch, A., and Hauser, A.E. (2014). Static and dynamic components synergize to form a stable survival niche for bone marrow plasma cells. Eur. J. Immunol. *44*, 2306–2317.

STAR★METHODS

KEY RESOURCES TABLE

REAGENT or RESOURCE	SOURCE	IDENTIFIER
Antibodies		
Anti-B220 AlexaFluor700, Clone RA3-6B2	Biolegend	Cat# 103232; RRID:AB_493717
Anti-Ly6G PE, Clone 1A8	Biolegend	Cat# 127608; RRID:AB_1186099
Anti-CD69 BV711, Clone H1.2F3	Biolegend	Cat# 104537; RRID:AB_2566120
Anti-GL7 AlexaFluor647	Biolegend	Cat# 144606; RRID:AB_2562185
Anti-GL7 Pacific Blue	Biolegend	Cat# 144614; RRID:AB_2563292
Anti-GL7 PerCP/Cy5.5	Biolegend	Cat# 144610; RRID:AB_2562979
Anti-PDL2 PeCy7, Clone B7-DC	Biolegend	Cat# 107214; RRID:AB_2728123
Anti-CD80 BV750, Clone B7-1	BD Biosciences	Cat# 747436; RRID:AB_2872118
Anti-CD73 APC, Clone TY/11.8	Biolegend	Cat# 127210; RRID:AB_11218786
Anti-CXCR3 BV605, Clone S18001A	Biolegend	Cat# 155915; RRID:AB_2892317
Anti-CD45 APC, Clone 30F11	Biolegend	Cat# 103112; RRID:AB_312977
Anti-CD45.1 Pacific Blue,Clone A20	Biolegend	Cat# 110722; RRID:AB_492866
Anti-CD45.2 Pacific Blue, Clone 104	Biolegend	Cat# 109820; RRID:AB_492872
Anti-IgM PECy7, Clone RMM-1	Biolegend	Cat# 406514; RRID:AB_10642031
Anti-IgG1 Biotin, Clone RMG1-1	Bioloegend	Cat# 406604; RRID:AB_315063
Anti-IgG2b Biotin, Clone RMG2b-1	Biolegend	Cat# 406704; RRID:AB_315067
Anti-IgG2a[b] Biotin, Clone 5.7	BD Biosciences	Cat# 553504; RRID:AB_394889
Anti-IgA AlexaFluor647	Southern Biotech	Cat# 1040-31; RRID:AB_2794377
InVivoMAb anti-mouse IFNg, Clone XMG1.2	BioXCell	Cat# BE0055; RRID:AB_1107694
InVivoMAb anti-mouse CD40L, Clone MR-1	BioXCell	Cat# BE0017-1; RRID:AB_1107601
InVivoMAb anti-mouse CXCR3, Clone CXCR3-173	BioXCell	Cat# BE0249; RRID:AB_2687730
InVivoMAb rat IgG2a isotype control, anti-trinitrophenol	BioXCell	Cat# BE0089; RRID:AB_1107769
InVivoMAb Armenian Hamster IgG Isotype Control; anti GST, Clone PIP	BioXCell	Cat# BE0260; RRID:AB_2687739
TotalSeq™-C0914 anti-mouse CD273, Clone B7-DC	Biolegend	Cat# 107229; RRID:AB_2860615
TotalSeq™-C0015 anti-mouse Ly-6G,Clone 1A8	Biolegend	Cat# 127657; RRID:AB_2819863
TotalSeq™-C0810 anti-mouse CD138, Clone 281-2	Biolegend	Cat# 142538; RRID:AB_2860696
TotalSeq™-C0093 anti-mouse CD19, Clone 6D5	Biolegend	Cat# 115571; RRID:AB_2832392
TotalSeq™-C0114 anti-mouse F4/80, Clone BM8	Biolegend	Cat# 123157; RRID:AB_2832437
TotalSeq™-C0228 anti-mouse CXCR3, Clone CXCR3-173	Biolegend	Cat# 126545; RRID:AB_2832454
TotalSeq™-C0197 anti-mouse CD69, Clone H1.2F3	Biolegend	Cat# 104551; RRID:AB_2832333
TotalSeq™-C0120 anti-mouse TCR β chain, Clone H57-597	Biolegend	Cat# 109259; RRID:AB_2819820
TotalSeq™-C0013 anti-mouse Ly-6C, Clone HK1.4	Biolegend	Cat# 128051; RRID:AB_2832461
TotalSeq™-C0014 anti-mouse/human CD11b, Clone M1/70	Biolegend	Cat# 101275; RRID:AB_2832272

(*Continued on next page*)

CellPress
OPEN ACCESS

Continued

REAGENT or RESOURCE	SOURCE	IDENTIFIER
TotalSeq™-C0117 anti-mouse I-A/I-E, Clone M5/114.15.2	Biolegend	Cat# 107659; RRID:AB_2832368
TotalSeq™-C0112 anti-mouse CD62L, Clone MEL-14	Biolegend	Cat# 104455; RRID:AB_2819800
TotalSeq™-C0106 anti-mouse CD11c, Clone N418	Biolegend	Cat# 117361; RRID:AB_2819834
TotalSeq™-C0103 anti-mouse/human B220, Clone RA3-6B2	Biolegend	Cat# 103273; RRID:AB_2832307
TotalSeq™-C0301 anti-mouse Hashtag 1, Clone M1/42; 30-F11	Biolegend	Cat# 155861; RRID:AB_2800693
TotalSeq™-C0302 anti-mouse Hashtag 2, Clone M1/42; 30-F11	Biolegend	Cat# 155863; RRID:AB_2800694
TotalSeq™-C0303 anti-mouse Hashtag 3, Clone M1/42; 30-F11	Biolegend	Cat# 155865; RRID:AB_2800695
Purified Goat anti-mouse IgG (minimal x-reactivity), Poly4053	Biolegend	Cat# 405301; RRID:AB_315005
Purified anti-mouse Ig light chain κ Antibody, Clone RMK-12	Biolegend	Cat# 407202; RRID:AB_345326
Mouse Anti-Hamster IgG Antibody, Clone MAH1.12	R and D Systems	Cat# MAB011; RRID:AB_357352
IgG from Mouse Serum	Sigma-Aldrich	Cat# I5381; RRID:AB_1163670
**Bacterial and virus strains**		
X31 influenza; H3N2 strain	Cloned and propagated in house	N/A
CFP-S-Flu; [S-eCFP/N1(PR8)].H1(PR8)	Generated, cloned and propagated in house	N/A
GFP-S-Flu; [S-eGFP/N1(PR8)].H1(PR8)	Provided by Alain Townsend, (Powell et al., 2012)	N/A
**Chemicals, peptides, and recombinant proteins**		
Pertussis Toxin Islet Activating Protein Salt-Free (PTX)	Quadratech Diagnostics Ltd.	Cat# 181
Clodronated liposomes (CLL)	Liposoma BV	Cat# CP-010-010
Control liposomes (PBS)	Liposoma BV	Cat# CP-010-010
Fixable Viability Dye e780	Life Technologies Ltd	Cat# 65-0865-14
Fingolimod (FTY720) HCl	Stratech	Cat# S5002
Collagenase D	Roche	Cat# 11088858001
DNase I	Merck	Cat# DN25
RNase A	Merck	Cat# R4875
Ultra TMB-ELISA Substrate Solution	Thermo Scientific	Cat# 34028
Streptavidin-BV605	Biolegend	Cat# 405229
Streptavidin-Horseradish Peroxidase	Jackson ImmunoResearch	Cat# 016-030-084; RRID: AB_2337238
Biotin-X-NHS	Sigma-Aldrich	Cat# 203188
**Critical commercial assays**		
Cytofix/Cytoperm W/GolgiPlug Kit	BD Biosciences	Cat# 555028, RRID:AB_2869013
MagniSort Mouse B cell Enrichment Kit	Thermo Fisher Scientific	Cat# 8804-6827-74, RRID:AB_2575267
**Deposited data**		
Murine bulk RNA-Seq: lung BRM (ivCD45$^-$ B220$^+$ CD69$^+$ GL7$^-$ dTomato$^+$ HA$^+$), splenic Bmem (B220$^+$ CD38$^+$ GL7$^-$ dTomato$^+$ HA$^+$) and splenic naïve B cell (B220$^+$ CD38$^+$ GL7$^-$ dTomato$^-$)	This paper	Gene Expression Omnibus: GSE183135; (https://www.ncbi.nlm.nih.gov/geo/query/acc.cgi?acc=GSE183135)

(*Continued on next page*)

Continued

REAGENT or RESOURCE	SOURCE	IDENTIFIER
Murine scRNA-Seq: Lung ivCD45⁻ ex-vivo CD45⁺ cells; d0 resting, D1 X31 rechallenge and D1 X31 rechallenge+CLL treatment	This paper	Gene Expression Omnibus: GSE194058
Experimental models: Organisms/strains		
Mouse: Rosa26-tdTomato: B6.Cg-Gt(ROSA)26Sortm9(CAG-tdTomato)Hze/J	Jackson Laboratory	Cat# 007909; RRID:IMSR_JAX:007909
Mouse: BLIMP1-mVenus: Prdm1-mVenus	Provided by M. Saitou, (Ohinata et al., 2008)	Riken accession CDB0460T
Mouse: AID-cre: B6.129P2-Aicdatm1(cre)Mnz/J	Jackson Laboratory	Cat# 007770; RRID:IMSR_JAX:007770
Mouse: CD19Cre/Cre: B6.129P2(C)-Cd19tm1(Cre)Cgn/J	Jackson Laboratory	Cat# 006785; RRID:IMSR_JAX:006785
Mouse: Rosa26-stop-YFP: B6.129X1-Gt(ROSA)26Sortm1(EYFP)Cos/J	Jackson Laboratory	Cat# 006148; RRID:IMSR_JAX:006148
Mouse: Ub-GFP: C57BL/6-Tg(UBC-GFP)30Scha/J	Jackson Laboratory	Cat# 004353; RRID:IMSR_JAX:004353
Oligonucleotides		
TaqMan Gene Expression Assay. Mm00434946_m1 Cxcl9, FAM-MGB	Life Technologies Ltd	Cat# 4331182
TaqMan Gene Expression Assay. Mm00445235_m1 Cxcl10	Life Technologies Ltd	Cat# 4331182
TaqMan Gene Expression Assay. Mm00441259_g1 Ccl3	Life Technologies Ltd	Cat# 4331182
TaqMan Gene Expression Assay. Mm01302427_m1 Ccl5	Life Technologies Ltd	Cat# 4331182
TaqMan Gene Expression Assay. Mm00443111 Ccl4	Life Technologies Ltd	Cat# 4331182
TaqMan Gene Expression Assay. Mm99999915_g1 Gapdh gene	Life Technologies Ltd	Cat# 4331182
Software and algorithms		
Flowjo, v10.8, Treestar Inc.	https://www.flowjo.com/	RRID: SCR_008520
Graphpad Prism v9	https://www.graphpad.com/scientificsoftware/	RRID: SCR_002798
Adobe Illustrator CS6	http://www.adobe.com/products/illustrator.html	RRID: SCR_010279
Imaris v9.2.1	http://www.bitplane.com/imaris/imaris	RRID: SCR_007370
Zen Digital Imaging for Light Microscopy	http://www.zeiss.com/microscopy/en_us/products/microscope-software/	RRID: SCR_013672
R Studio	https://rstudio.com/	RRID:SCR_000432

RESOURCE AVAILABILITY

Lead contact

Further information and requests for resources and reagents should be directed to and will be fulfilled by the lead contact, Tal Arnon (tal.arnon@kennedy.ox.ac.uk).

Materials availability

This study did not generate new unique reagents.

Data and code availability

- RNA-Seq data generated in this work have been deposited at GEO and are publicly available as of the date of publication. Accession numbers are listed in the key resources table.
- This paper does not report original code.
- Any additional information required to reanalyze the data reported in this paper is available from the lead contact upon request.
- All data are available in the manuscript or the supplemental information.

CellPress
OPEN ACCESS

EXPERIMENTAL MODEL AND SUBJECT DETAILS

Mice

Male and female mice aged 8-16 weeks were used for all experiments. C57BL/6 (B6, CD45.2+) or B6 Ly5.2 (CD45.1+) mice were purchased from Charles River. CD19 KO mice were on a B6 background and were generated by intercrossing CD19 $Cre^{+/+}$ (Rickert et al., 1995) mice to obtain $CD19^{Cre/Cre}$ mice. Mice expressing GFP under the human ubiquitin promoter (Ub-GFP, 004353; Tg(UBC-GFP)30Scha/J), MGI:2158677), AID-Cre (007770; B6.129P2Aicdatm1(cre)Mnz/J), Rosa26-stop-tdTomato (007914; B6.Cg-Gt(ROSA)26Sortm14(CAG-tdTomato)Hze/J), and Rosa26-stop-YFP (006148; B6.129X1-Gt(ROSA)26Sortm1(EYFP)Cos/J) were from Jackson Laboratories. $Prdm1^{mVenus}$ ($BLIMP1^{mVenus}$) mice were described previously (Ohinata et al., 2008). (Riken Accession CDB0460T, http://www2.clst.riken/jp/arg/TG%20mutant%20mice%20list.html) BAT mice experiments were performed using marrow chimeras, in which lethally irradiated C57BL/6 mice were reconstituted with bone marrow from $BLIMP1^{mVenus}$ $AID^{Cre/+}$ $Rosa26^{stop\text{-}tdTomato}$ animals. We used this approach to allow generation of large cohort of triple positive mice, needed for the study. To generate chimeras, 8-12w old C57BL/6 mice were lethally irradiated (11Gy) in two dosages separated by 4h, followed by injection of >5x10^{6} $Blimp1^{mVenus}$ $AID^{Cre/+}$ $Rosa26^{stop\text{-}tdTomato}$ bone marrow cells per mouse.

Animals were bred and maintained under specific pathogen-free (SPF) conditions in accredited animal facilities at Kennedy Institute of Rheumatology, University of Oxford and experiments were in accordance with the UK Scientific Procedures Act (1986) under a Project License (PPL) authorized by the UK Home Office.

Viruses

For infections using non-fluorescent influenza virus, we used the A/HK-x31 (x31, H3N2) strain. The reporter strains expressing GFP (GFP-S-Flu; [S-eGFP/N1(PR8)]) or CFP (CFP-S-Flu; [S-eCFP/N1(PR8)]) were generated using the Cambridge strain of A/Puerto Rico/8/34 (Powell et al., 2012, 2019). The viruses used were cloned and propagated in-house.

METHOD DETAILS

Influenza infection, rechallenge with VLPs and immunization

For primary infection, mice weighing >20g were anaesthetised using isoflurane and intranasally administered with 2x10^{4} PFU of X31 influenza A virus in PBS. Mice were monitored for 14d following infection and all displayed characteristic weight loss. For rechallenge experiments, high-dose challenge was performed using 1x10^{6} PFU of the X31 or CFP-S-Flu strain. For VLP challenge of influenza-immune animals, Qβ-VLPs were diluted in PBS to give a final i.n. dosage of 50μg in 50ul. For immunization with sheep red blood cell (SRBC), 3ml SRBC (Fisher Scientific UK Ltd) were washed in 20ml PBS twice, then resuspended in 5ml and given subcutaneously in 4 sites (50μl/site).

Influenza hemagglutinin (HA) production and biotinylation

cDNA encoding X31 H3 was generated by PCR. This sequence was codon-optimised and synthesised by GeneArt, introducing the Y98F mutation to reduce non-specific binding to sialic acid (Frank et al., 2015). This codon corresponded to residue 114 in our virus H3 haemagglutinin. This HA^{Y114F} sequence was ligated into a retroviral plasmid containing a thrombin cleavage site, foldon trimerization sequence, Bir biotinylation site and His tag for purification (as described in Figure S1A). The entire construct was then transferred into pQCXIX (Clontech) containing an IRES-eGFP expression cassette.

Retroviral particles were packaged in GP-293T cells by co-transfection with pVSVg, and 293T cells were transduced. GFP^{+} 293T cells were sorted to establish a stable HA-secreting cell line. For HA purification, supernatants from cultures of these cells were diluted 1:1 in binding buffer (PBS, 0.05% sodium azide, pH8) and incubated with Ni-NTA beads (Qiagen) overnight. Beads were then applied to a centrifuge column (Pierce) and washed in buffers containing imidazole (10mM, 20mM in PBS pH8) before elution (250mM imidazole, PBS). Fractions were pooled and dialysed in PBS using a 10kDa dialysis cassette (Bio-Rad).

For biotinylation, HA was incubated with biotin at a 1:4.5 molar ratio (HA:biotin) with 0.5M EDTA and 0.1M sodium bicarbonate for 2h. Excess biotin was removed by dialysis as above. Staining concentrations were optimised for each preparation of HA-bio, but optimal labelling was typically of the range of 30-50ng/10^{6} lymphocytes.

VLP production

RNA sufficient and RNA-free Qβ-VLPs were produced in E. coli and purified by chromatography by A. Cruz-Gomes as previously described (Gomes et al., 2017; Kozlovska et al., 1993). For RNA removal, Qβ-VLPs were buffer-exchanged by diafiltration to 20mM HEPES, pH7. VLPs were concentrated to 2mg/ml and incubated with 1mg/ml RNAse A at 37°C for 3h. Degraded RNA and RNase was removed by diafiltration against 20mM HEPES followed by PBS. After diafiltration, RNA removal was confirmed by running a native 0.8% agarose gel with nucleic acid stain, followed by coomassie stain to confirm RNA degradation.

Generation of CFP-S-Flu

Single-cycle fluorescent reporter strain of influenza A virus was prepared as previously described (Powell et al., 2012, 2019). Briefly, plasmids encoding eCFP with SapI restriction sites were synthesised by GeneArt and cloned into the pPolI vector. 293T cells were transfected with Lipofectamine 2000 (Invitrogen), in the presence of expression plasmid pCDNA3.1, which contained expression of

full-length HA. This permits viral coating of HA and secretion from 293T. The resulting viral particles were used as seed to infect a stable H3-expressing MDCK-SIAT1 cell line, thus generating viral particles which carry the genes for all IAV proteins except HA, which is replaced by eCFP, but that are coated with cell-derived H3 to allow single cycle infection. Stocks of this CFP-S-Flu were produced by infecting HA-expressing SIAT1 cells in DMEM (0.1%BSA, 1%P/S) and 2h later adding 1μg/ml TPCK-treated trypsin (Sigma T-1426). The virus supernatant was collected after 48h of incubation at 37°C.

Flow cytometry, *in vivo* labeling, and cell sorting

In vivo labelling was performed as described previously. Mice were intravenously injected with 2.5μg PE/Pacific Blue-conjugated anti-CD45 or anti-CD19 in PBS. After 4min mice were euthanized and lungs perfused with 10ml cold PBS through the right ventricle. Lungs were removed and roughly dissected with scissors before digestion in 1mg/ml collagenase D (Roche) and 10μg/ml DNAseI in RPMI for 45min at 37°C. Tissue was homogenised through a 70μm mesh and for experiments assessing BRM, lymphocytes were enriched by Ficoll-Paque density centrifugation (GE Healthcare). Cell suspensions were incubated with FC block in FACS buffer (2% FBS, 0.1% Sodium Azide, 1mM EDTA in PBS) for 15min and then stained in FACS buffer using predetermined optimal antibody concentrations for 30min. Cells were then washed and labelled with secondary labelling agents for 20min. For intracellular staining of antibody isotypes, Cytofix/Cytoperm Staining Buffer Kit (BD Biosciences) was used as per manufacturer's instructions. Data acquisition was performed using a BD Fortessa X20 (BD Biosciences) and analysed using FlowJo v10.8 (Tree Star Inc.). For BRM cell phenotypic characterisation (Figures 1B and 1C), data acquisition was performed on a Cytek Aurora (Cytek Bioscienes). For cell sorting, samples were prepared as above and sorted using a FACSAria III (BD Biosciences).

Bulk and single cell RNA-seq analysis

For bulk RNAseq analysis, BAT mice were infected with X31 influenza. 45 days later, lung BRM cells (defined as i.v. $CD45^-$ $B220^+$ $CD69^+$ $GL7^-$ AID-$tdTomato^+$ HA^+), splenic memory B cells ($B220^+$ $GL7^-$ $CD38^+$ AID-$tdTomato^+$ HA^+) and splenic naïve B cells ($B220^+$ $GL7^-$ $CD38^+$ AID-$tdTomato^-$) were sorted from samples derived from mice that have been *in vivo* labelled and perfused, and which have been subjected to the same digestion and processing procedures, as described above. In each experiment, 200 cells of each population were sorted from a pool of cells derived from 6 mice. Cells were collected directly into lysis buffer (total n=18). Three experiments were performed. In the first two experiments two technical replicates were included. In the third experiment a single technical replicate was performed.

Library construction was performed as previously described, using a Smart-seq2 protocol adapted to low cell numbers (Radtke and Bannard, 2018). 19 cycles of preamplification were used for all samples. cDNA purification was performed using Ampure XP beads (Beckman Coulter). Libraries were analyzed with a High Sensitivity Analyser (Agilent) and cDNA tagmentation was performed with the Nextera XT DNA Sample Preparation kit (Illumina). Libraries were quantified using PicoGreen (Illumina), sized using the High Sensitivity Analyser and equal amounts of tagmented cDNA from each library were pooled. Sequencing was performed on an Illumina NextSeq500 using FC-404-2005 NextSeq 500/550 High Output Kits v2 (75 cycles).

QC analysis was preformed with the fastQC package (http://www.bioinformatics.babraham.ac.uk/projects/fastqc). Reads were then aligned using STAR (Dobin et al., 2013) against the mouse genome assembly (GRCm38 (mm10) UCSC transcripts). Gene expression levels were quantified as read counts using the featureCounts function (Liao et al., 2014) from the Subread package (Liao et al., 2013)(The Subread package: a toolkit for processing next-gen sequencing data. http://subread.sourceforge.net SourceForge package version 1.4.5.) with default parameters. The read counts were used for the identification of global differential gene expression between specified populations using the edgeR package (Robinson et al., 2010). RPKM values were also generated using the edgeR package. Genes were considered differentially expressed between populations if they had an adjusted p-value (FDR) of less than 0.05. The Gene Ontology analysis was performed using the goseq R package (Young et al., 2010) accounting for gene length bias and GO categories were considered significantly enriched if they had an FDR less than 0.05. Inter- and intragroup variability was assessed by principal component (PC) analysis applied to the filtered and variance stabilised transformed (VST) count data generated using the DESeq2 package (PCA plots applied to the top 500 most variable genes), and by Spearman's correlation (r_s) values between samples calculated from TMM-normalised and filtered count data (gene counts were filtered for features detected at least 50x across all samples). Technical replicates were treated as separate libraries for PC analysis and collapsed for differential expression and correlation analyses. Data have been deposited in NCBI's Gene Expression Omnibus and are available under GEO Series accession number GSE183135 (https://www.ncbi.nlm.nih.gov/geo/query/acc.cgi?acc=GSE183135).

For single cell RNAseq, B6 mice were infected with X31 influenza and were allowed to reach a memory phase (day 42 post primary infection). The mice were divided into 3 groups (n=3). One group was assessed without rechallenge. The second group was pretreated with PBS liposomes intranasally on days 6 and 3 before being rechallenged with X31 influenza. The third group was pretreated as above with CLL liposomes followed by rechallenge with X31 influenza. One day after rechallenge, the mice were in vivo labelled with CD45 for 4min prior to tissue collection. Lungs were processed, stained and sorted as described above. Approximately 20,000 cells per sample pool were loaded onto the 10X Genomics Chromium Controller (Chip K). Gene expression, feature barcoding and BCR sequencing libraries were prepared using the 10x Genomics Single Cell 5' Reagent Kits v2 (Dual Index) following manufacturer user guide (CG000330 Rev B). The final libraries were diluted to ∼10nM for storage. The 10nM library was denatured and further diluted prior to loading on the NovaSeq6000 sequencing platform (Illumina, v1.5 chemistry, 28bp/98bp paired end for gene expression and feature barcoding, 150bp paired end for BCR libraries).

Sequence reads were mapped using CellRanger multi (version 6.0.0) with the 10x mouse reference transcriptome (version 2020-A). Cells were demultiplexed using GMM-Demux (version 0.2.1.3) (Xin et al., 2020). The data were analysed in two stages: an "initial" analysis with permissive thresholds was used to help identify and remove low quality cells, contaminants and doublets before a "final" analysis was performed. For the initial analysis we selected cells with >200 genes and <10% mitochondrial reads (n=19,146 cells), pre-processed the data with SCANPY (Wolf et al., 2018) (version 1.8.1), integrated the cells from the different samples with Harmony (Korsunsky et al., 2019) and identified and characterised the cell clusters using pipeline_scxl.py (https://github.com/sansomlab/tenx) (COVID-19 Multi-omics Blood Atlas (COMBAT) Consortium et al, 2021). This analysis identified (i) two clusters of apoptotic (or otherwise of low quality) B and T cells marked by very low expression of ribosomal genes and other ubiquitously expressed cytoplasmic RNAs (such as Tpt1, Tmb4X) together with high expression of nuclear lncRNAs (such as MALAT1) and higher expression of mitochondrial genes, (ii) a small cluster of doublets that expressed markers of both B and T cells and (iii) a cluster of contaminating thymocytes (Rag1, Rag2, CD4/CD8 double positive T cells) that was largely (80.2%) comprised of cells from a single replicate (CLL-treated rechallenged replicate 3). For the final analysis we selected demultiplexed singlet cells (GMM-Demux confidence >=0.8) with > 200 genes and < 5% mitochondrial genes. Based on the initial analysis we additionally filtered out, (i) all of the cells from the heavily thymocyte-contaminated replicate (CLL-treated rechallenged replicate 3), (ii) n=125 cells from other replicates that were also present in the thymocyte cluster and (iii) any remaining cells that had been identified as low-quality/apoptotic B and T cells (n=838 cells). To remove doublets, we next excluded cells with scrublet (Wolock et al., 2019) scores>mean + three standard deviations (n=292 cells) and then removed any remaining cells that had been identified as B-T doublets in the initial clustering analysis (n=96 cells). The data from the sanitised set of cells (n=13,172 cells) was total count normalised and log1p transformed with SCANPY (Wolf et al., 2018) (version 1.8.1). Highly variable genes (HVG) were identified within each sample separately (n_top_genes=2000, flavour="Seurat_v3", using counts) and then combined, retaining only the subset that was discovered in at least 2 samples (n=2906 genes). The effect of total UMI number was regressed out and the data scaled. The data were integrated with the python SCANPY implementation of Harmony ("harmony_integrate") using n = 30 principal components ("key" parameter set to the sample identifier). The integrated data was then analysed using pipeline_scxl.py (https://github.com/sansomlab/tenx). An exact neighbor graph was computed with Scikit-learn (as implemented in scVelo; Bergen et al., 2020) using n=30 Harmony components, n=20 neighbours and the euclidean distance metric. This neighbor graph was used to compute the UMAP and for Leiden clustering across a range of resolutions. The final cluster assignments were prepared by combining/merging the assignments from the different clustering resolutions to parsimoniously describe the major cell types/subtypes present in the different regions of the manifold for subsequent analyses. Conserved cluster markers were identified as those that were commonly identified for the clusters in each of the three conditions (Seurat FindMarkers, Wilcoxon test, BH adjusted $p < 0.05$). For composition and within-cluster differential expression analysis the dendritic cell (DC) clusters were merged into a single DC cluster. As cell numbers for individual replicates precluded a pseudobulking-based approach, exploratory within cluster differential expression analysis between the cells of the "rechallenged" and "CLL-treated rechallenged" conditions was performed using the Seurat (Hao et al., 2021) FindMarkers function (Wilcoxon test, BH adjusted $p < 0.05$). The set of secreted cytokines/chemokines that was significantly down-regulated in the "rechallenged + CLL" cells vs the "rechallenged" cells was obtained by intersecting the differential expression results with the sets of genes present in the KEGG "cytokine-cytokine receptor interaction" (mmu04060) and "chemokine signalling pathway" (mmu04062) genesets (excluding genes known to encode surface receptors or intracellular signalling molecules). Over-representation of Gene Ontology Biological Processes amongst the differentially expressed genes was computed using one-sided Fisher's exact tests as implemented in gsfisher (https://github.com/sansomlab/gsfisher/) using a background gene universe comprised of genes detected in at least 10% of the cells of at least one cluster in one of the conditions. Geneset scores were computed using the AUCell algorithm (Aibar et al., 2017) and were visualised without thresholding.

CITE-seq (Stoeckius et al., 2017) counts were normalized following a background aware library-size approach (As described in https://github.com/Bioconductor/OrchestratingSingleCellAnalysis v1.0.6). First, we estimated the background signal for each antibody as the lower mode of a fitted bimodal distribution using the "inferAmbience" function implemented in the "DropUtils" R library (Lun et al., 2019). Then, the library size factor for each cell was calculated as the median of the ratios of the features read outs in relation to the estimated backgrounds. The median-based library size factors were then used for scaling normalization and log-transformation with the "logNormCounts" function of the R "scuttle" library (McCarthy et al., 2017). Normalization was performed independently for each sample batch to account for differences in background staining levels between the batches.

Data have been deposited in NCBI's Gene Expression Omnibus and are available under GEO Series accession number GSE194058 (https://www.ncbi.nlm.nih.gov/geo/query/acc.cgi?acc=GSE194058).

Quantitative PCR

Quantitative PCR was performed on lung alveolar macrophages (i.v. $CD45^-$ $F4/80^+$ $CD11c^+$ $SiglecF^+$), monocytes (i.v. $CD45^-$ $F4/80^-$ $CD11c^-$, $Ly6g^-$ $Ly6c^+$) and neutrophils (i.v. $CD45^-$ $F4/80^-$ $CD11c^-$ $Ly6g^+$ $Ly6c^-$) and dendritic cells (i.v. $CD45^-$ $F4/80^-$ $Ly6G^-$ $MHCII^{hi}$ $CD11c^+$), sorted directly into lysis buffer with 2-ME (Qiagen RNeasy Plus Micro Kit). Reverse transcription was performed with High-Capacity cDNA Reverse Transcription Kit (Applied Biosystems, 4368814). Quantitative PCR was performed using the following taqman assays: GAPDH (Mm99999915), *Cxcl9* (Mm00434946), *Cxcl10* (Mm00445235), *Ccl3* (Mm00441259), *Ccl5* (Mm01302427) and *Ccl4* (Mm00443111). Data acquisition was performed on an Applied Biosystems Viia 7 Real-Time PCR System. All gene expression were normalized to an internal housekeeping gene (GAPDH) mRNA and calculated as $2^{-(CThk-CTgene)*10,000}$.

Adoptive cell transfer

CD19ko mice were adoptively transferred i.v. with ~10x10^6 B cells purified B cells isolated from BAT splenocytes using Magnisort B cell Enrichment Kit (Affymetrix eBioscience) as per manufacturers protocol.

Live explant imaging

Live imaging was performed as previously described (Lelkes et al., 2014; Thornton et al., 2012). Briefly, mice were euthanized with i.p. pentobarbitone and then perfused with 10ml cold PBS through the right ventricle. Mice were then injected with 1-1.5ml of ~30-35°C 1% low-melt agarose in PBS into the lungs via a tracheal incision. Individual lobes were dissected and stored in RT RPMI before being sectioned at 450μm on a vibratome (Campden Instruments Ltd.). Sections were mounted on plastic coverslips using Vetbond tissue glue (3M), placed in RT RPMI and immediately imaged.

During two-photon imaging an explant setup was established to maintain sample viability at 37°C under continuous flow of RPMI perfused with oxygen and carbon dioxide. Explants were imaged on an upright Zeiss LSM 880 microscope, equipped with two Mai Tai lasers (690-849nm, 850-990nm), using a primary excitation wavelength of 930nm, with an Apochromat 20x1.0 DIC VIS-IR D=0/0 (UV) objective lens. Series of planes of ≤3μm z-spacing spanning a depth of 80-110μm were recorded every 15-30s. Videos were made and analysed using Imaris (Bitplane, v9.2.1).

Imaging of static lung sections using TPLSM

All quantitative assessments of BRM cell or PC distribution, including calculations of clustering L values and cell densities in uninfected and infected sites, were done by analysing large tiles of static lung sections using TPLSM. Samples were prepared similarly to the described above for explant live imaging. However, instead of mounting the freshly cut tissues in a perfusion chamber, the samples were placed in room temperature PBS without perfusion. This maintained the overall architecture of the samples during imaging, but prevented cell movement, allowing acquisition of large tiles.

Immunofluorescence staining and confocal microscopy

For immunofluorescence, mice were euthanized with pentobarbitone, and lungs were inflated with optimal cutting temperature compound (OCT, TissueTek), as described (Lelkes et al., 2014; Thornton et al., 2012). Individual lobes were dissected and fixed in 1% PFA in PLP buffer (1mM sodium periodate, 0.2M L-lysine, pH7.4) for 24h. Lung lobes were washed and immersed in 30% sucrose for cryoprotection before embedding in OCT. 16-20μm sections were taken on a cryostat (Leica Biosystems). Sections were blocked with serum and FC block for 30min. After staining, slides were washed in PBS and mounted using fluoromount-G (Cambridge Bioscience Ltd.). Confocal microscopy was performed on an upright Zeiss LSM880.

Image acquisition and analysis

Image files obtained from confocal or two-photon microscopes collected in Zen Black were analysed using Imaris software (Bitplane, v9.2.1). Cells were tracked in Imaris using the surface function. All tracks were manually examined and verified. Migratory parameters were calculated using Imaris (Bitplane) and MATLAB (MathWorks), as described before (Allen et al., 2007). Displacement was calculated for 30min tracks.

For calculation of Ripley's K statistic, images were collected from large tiles of static lung sections (Z=80-150μm) using TPLSM. The Imaris' Spots function was used to define BRM cells and PCs. Spots were manually verified before being exported and used with image metadata to supply an R script with cell coordinates and image boundaries that allowed the generation of spatial point patterns. The spatstat package in R was used to calculate Ripley's K and L values using Kest and Lest functions. In all plots displaying L values, each circle represents the mean L value calculated for one mouse (+/-SD), based on multiple imaged tiles collected from each mouse.

For determination of infection foci, images were collected from large tiles of static lung sections (Z=80-150μm) using TPLSM. We used the Spots function in Imaris to generate points based on the fluorescent signal from CFP-S-Flu. Spot coordinates and image parameters were exported for use in the spatstat package in R, where the quadratcount function was used to assess the density of infected cells across the tiled fluorescent image, and areas with infected cells at a density of >225cells/mm3 were classified as infected. Subsequently, BRM and PC densities within these regions were quantified using a similar approach. Plots displaying densities in uninfected vs. uninfected sites show pair of points that represent the average density of BRM cells or alveolar PCs in one mouse, obtained by averaging data from multiple large tiles per animal. In plots displaying ratios of densities of cells in uninfected and infected sites, each circle represents the mean densities measured in one mouse, obtained by averaging data from multiple large tiles per animal.

In all cases when quantifying cell positioning in large tiles, at least 2 tiled images per animal were analysed and means were plotted.

FTY720 administration

FTY720 (Stratech, cat. S5002) was administered intravenously at 1mg/kg in PBS 2 days prior to rechallenge, as previously described (Matloubian et al., 2004). For experiments requiring analysis at day 4 post infection, treatment was administered a second time at day 2 after rechallenge.

Cell depletion, antibody treatments and PTX administration

Depletion of alveolar macrophages was performed by intranasal CLL administration, as previously described (Leemans et al., 2001). Mice were anaesthetised and 45μl of Clodrosome® (Liposoma BV) was administered two times each on d-6 and d-3 before influenza rechallenge.

Anti-CD40L (clone MR1, BioXCell) was administered in a regimen that successfully diminished lung GC B cells, as previously reported (Allie et al., 2019). Briefly, 250μg anti-CD40L was delivered intravenously to X31-immune mice every 2 days for 10 days (day 28-38 post infection). Anti-CXCR3 (500ug, Cxcr3-173, BioXCell) was given once i.v. 24h before rechallenge. Anti-IFNγ (100ug, XMG1.2, BioXCell) was administrated i.v. 24 hours before rechallenge and 1h following rechallenge.

In experiments using pertussis toxin (PTX, Quadratech Diagnostics Ltd.), influenza-immune mice were rechallenged with CFP-S-Flu and 2hrs later PTX was administered intravenously at a dose of 400μg/kg as previously reported to impact the in vivo migration of B cells (Fooksman et al., 2010).

ELISA

Influenza-specific IgG was detected by coating Nunc-immmuno MaxiSorp 96-well plates (Fisher Scientific, 10394751) with heat-inactivated X31 influenza overnight at 4C. Hamster IgG was captured with anti-hamster (MAH1.12, R&D Systems MAB011). Wells were blocked with 3% BSA in PBS+0.05% Tween20 at RT for 1hr, then samples were incubated at RT for 2h. For standardisation, mouse IgG (Sigma, I5381) and hamster IgG (BioXCell, ♯BE0260) was used. Secondary staining was performed using biotinylated goat anti-mouse-IgG followed by SA-HRP, or anti-Hamster-HRP(Jackson ImmunoResearch). Ultra-TMB-ELISA Substrate (Sigma, 34028) was used for development and optical densities were quantified using a SpectroSTAR Nano microplate reader (BMG Labtech).

QUANTIFICATION AND STATISTICAL ANALYSIS

Statistical parameters including number of mice and number of replicates are described in figure legends. Error bars represent the mean±SD throughout. Where normal distribution could be determined, comparisons between groups were made using unpaired Student's t-tests (Figures 5C, 6E, 6F, S1C, S2B, S2D, and S4C). When comparing infected and un-infected areas from the same mice we used paired Student's t-tests. For multiple comparisons Ordinary One-way ANOVA was used in Figures 1C, 4E, and 7D, while Kruskal-Wallis test was used in Figures 2D and 6C. Wilcoxon tests were used in comparisons in Figure 7B. In all other cases statistical comparisons were performed using Mann-Whitney U tests.

Graphpad Prism 8 (GraphPad software Inc.) was used to perform statistical analysis and for graphical representation of data. P values were calculated and represented as follows: *($p < 0.5$); **($p < 0.01$); ***($p < 0.001$);

****($p < 0.0001$).

Immunity, Volume *55*

Supplemental information

Secondary influenza challenge triggers resident memory B cell migration and rapid relocation to boost antibody secretion at infected sites

Andrew J. MacLean, Niamh Richmond, Lada Koneva, Moustafa Attar, Cesar A.P. Medina, Emily E. Thornton, Ariane Cruz Gomes, Aadil El-Turabi, Martin F. Bachmann, Pramila Rijal, Tiong Kit Tan, Alain Townsend, Stephen N. Sansom, Oliver Bannard, and Tal I. Arnon

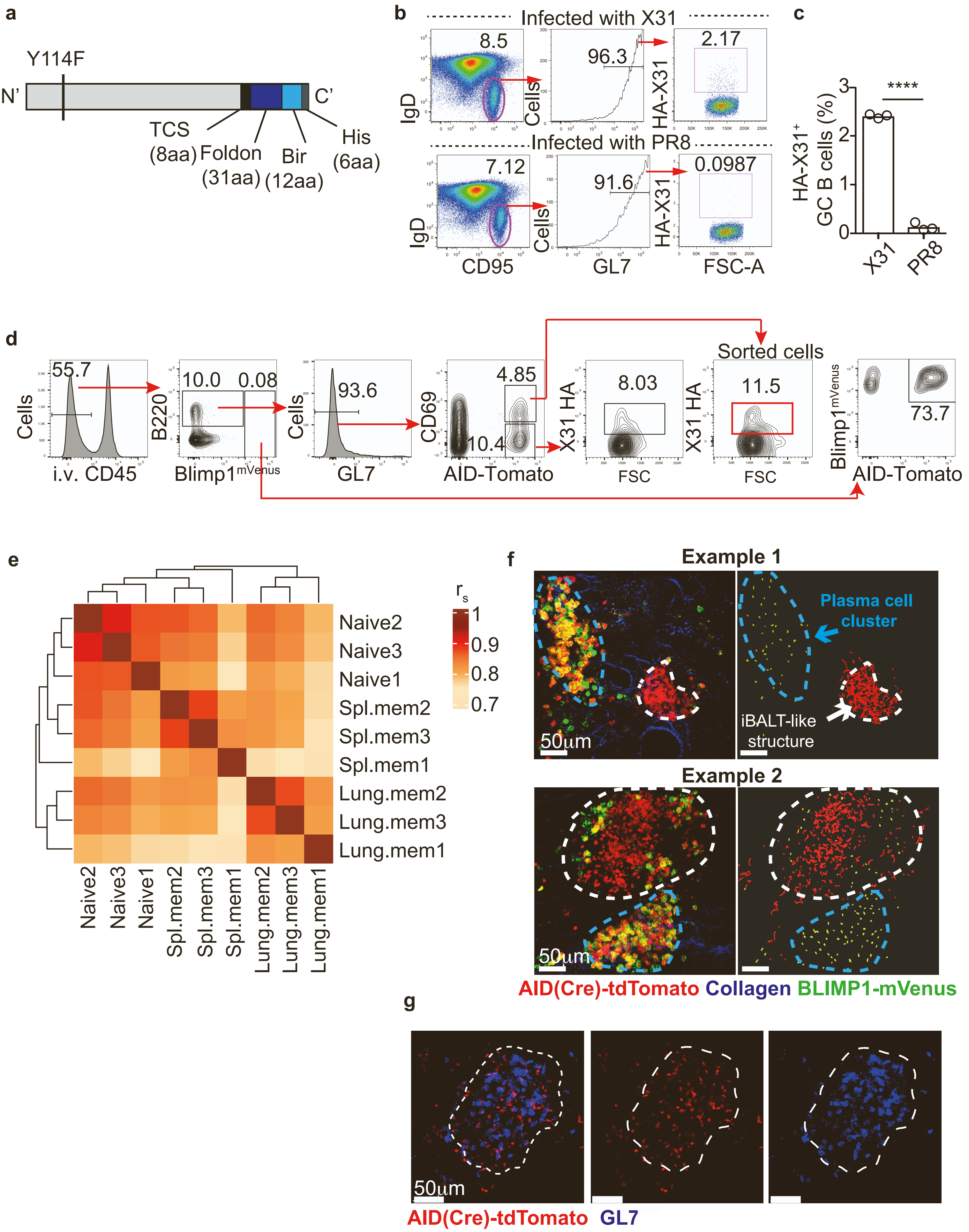

a
Y114F
N'
C'
TCS (8aa)
Foldon (31aa)
Bir (12aa)
His (6aa)
b
Infected with X31
Infected with PR8
8.5
96.3
2.17
7.12
91.6
0.0987
IgD
CD95
Cells
GL7
HA-X31
FSC-A
c
HA-X31+ GC B cells (%)
****
X31
PR8
d
55.7
10.0
0.08
93.6
4.85
10.4
8.03
11.5
73.7
Sorted cells
Cells
i.v. CD45
B220
Blimp1mVenus
GL7
CD69
AID-Tomato
X31 HA
FSC
e
rs
1
0.9
0.8
0.7
Naive2
Naive3
Naive1
Spl.mem2
Spl.mem3
Spl.mem1
Lung.mem2
Lung.mem3
Lung.mem1
f
Example 1
Plasma cell cluster
iBALT-like structure
50μm
Example 2
AID(Cre)-tdTomato Collagen BLIMP1-mVenus
g
50μm
AID(Cre)-tdTomato GL7

Supplementary Fig. 1 Detection of germinal centre B cells in influenza infected mice, related to Fig.1 (a-c) Detection of X31 hemagglutinin (X31-HA) specific B cells. (a) Construct design for HA probe. Y114F mutation (equivalent to Y98F in other strains) to reduce sialic acid binding, thrombin cleavage site (TCS), Foldon domain, BirA biotinylating site, and 6xHis-tag tag to allow purification. (b) Representative FACS plots displaying HA staining (Biotinylated) of GC B cells from mediastinal LN of X31 and PR8-infected mice at day 8 post infection, (c) enumerated for multiple mice. Each circle represents one mouse. (d) FACS gating strategy for sorting lung $CD69^{+}$ HA^{+} BRM cells for RNA-seq analysis and to quantify lung PCs and BRM cells. Figure shows representative FACS plots of lungs from infected BAT chimeras > 6 weeks post infection with X31 influenza. Mice were in vivo labelled with anti-CD45 (i.v. CD45) 4 mins before tissue collection. Plots are pre-gated on live cells. The gate used for sorting of HA^{+} $CD69^{+}$ BRM cells is highlighted in red. (e) Dendrogram and symmetrical heatmap visualisation of bulk RNA-seq correlation matrix showing inter-sample Spearman coefficients (r_s) between naïve, and memory B cells derived from the spleen (Spl.mem) and lung (Lung.mem). Scale bar represents the range of the correlation coefficients displayed. (f) Snapshots from videos of live explant BAT mice during the memory phase (42 days post primary infection) acquired with TPLSM. White dotted line outlines iBALT-like structures. Blue dotted line delineates cluster of PCs. Example 1 shows an iBALT-like structure containing $tdTomato^{+}mVenus^{-}$ cells with no PCs. Example 2 shows an iBALT-like structure containing $tdTomato^{+}mVenus^{-}$ cells surrounded by PCs. In both examples, a PC cluster containing primarily $tdTomato^{+}mVenus^{+}$ PCs is present adjacent to the iBALT-like structure. In each example, snapshots of cell distribution (left) and tracks of migrating cells (right) are shown. (g) Confocal microscopy of lungs from BAT mice stained with anti-GL7 (blue) 42 days post primary infection.

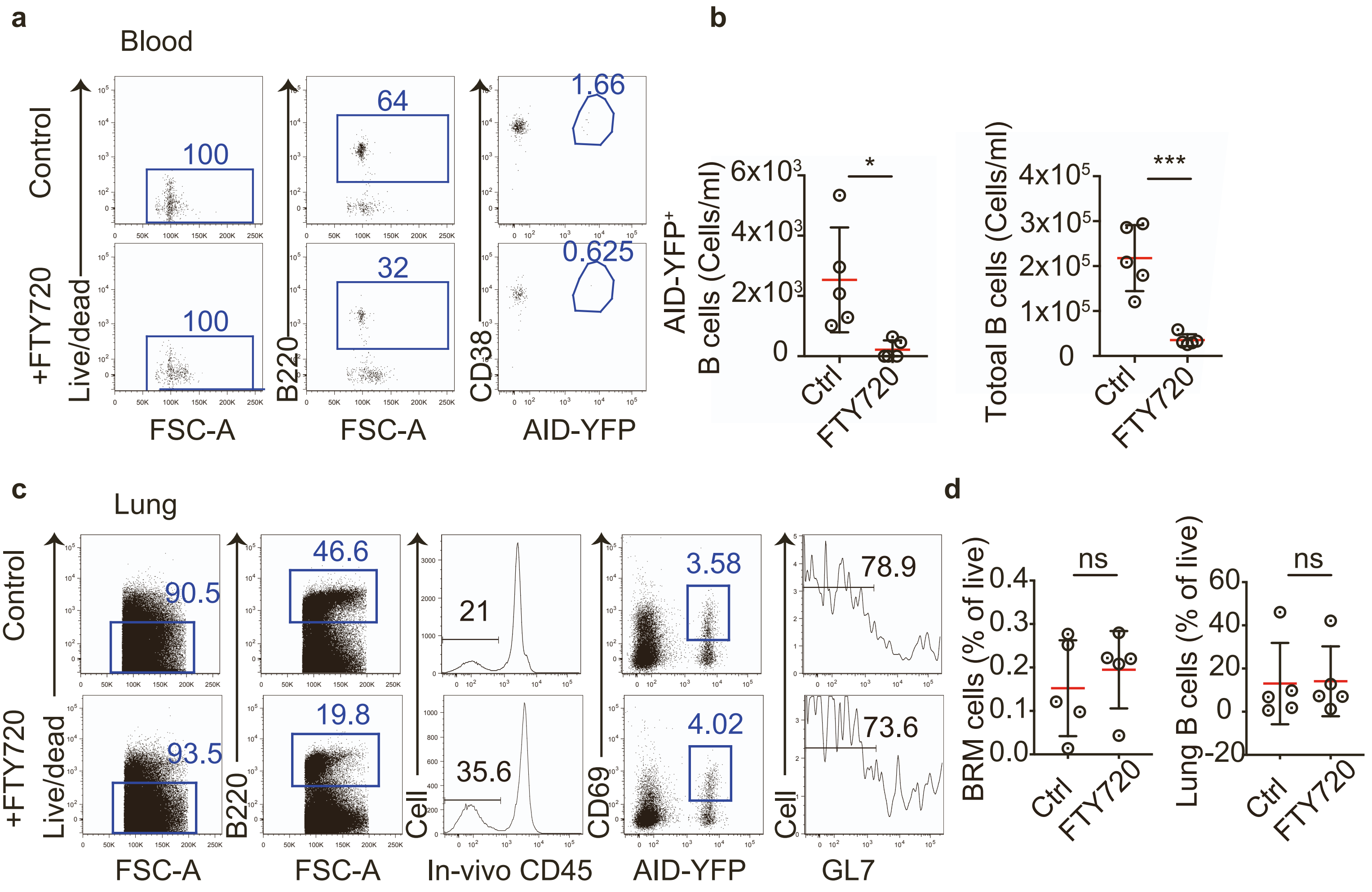

Supplementary Fig. 2. FTY720 treatment sequesters memory B cells in SLOs and removes them from the circulation, related to Fig.2

AID$^{Cre/+}$ Rosa26$^{-stop-EYFP}$ mice were infected with influenza. 45 days later, mice were injected in vivo with anti-CD45 antibodies prior to collection of blood and lung. When administered, FTY720 was injected i.v. 2 days prior to analysis. (a) Representative FACS plots showing abundance of memory/activated B cells (defined as Live B220^{+} CD38^{+} EYFP^{+}) in blood of FTY720 treated and untreated mice. (b) Memory/activated B cells (defined as in 'a') and naïve B cell (defined as Live B220^{+} CD38^{+} YFP^{-}) counts within blood. (c) Representative FACS plots of lung BRM cells (identified as Live B220^{+} i.v. CD45^{-} CD69^{+} YFP^{+} GL7^{-}). (d) Summary of lung BRM cell and total lung B cell (Live B220^{+} i.v. CD45^{-}) frequencies in FTY720 treated and untreated animals. Data shows results of one experiment out of 2 performed. Each circle represents one mouse.

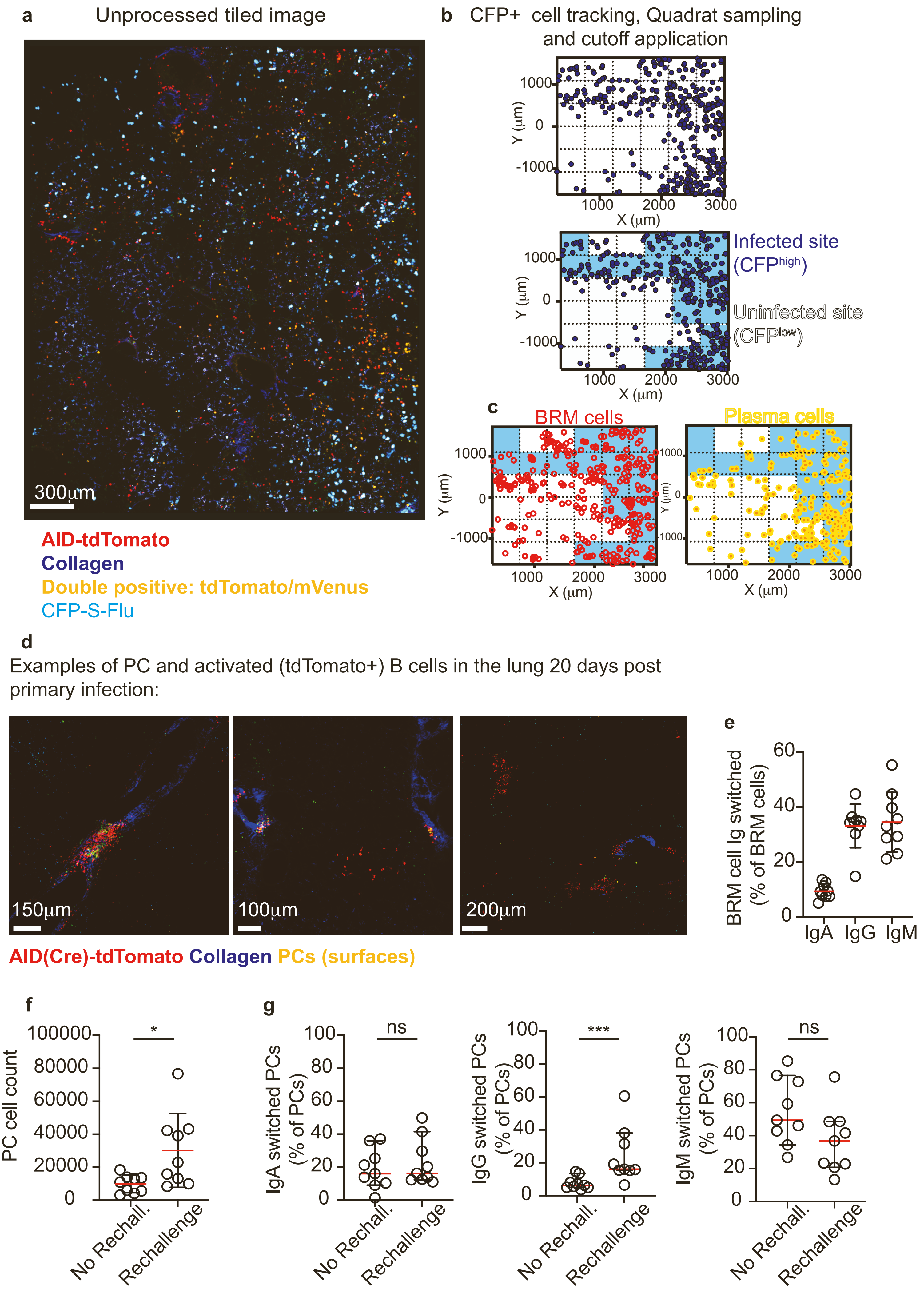

a
Unprocessed tiled image
300μm
AID-tdTomato
Collagen
Double positive: tdTomato/mVenus
CFP-S-Flu
b
CFP+ cell tracking, Quadrat sampling and cutoff application
Y (μm)
X (μm)
1000
0
-1000
1000
2000
3000
Infected site (CFPhigh)
Uninfected site (CFPlow)
c
BRM cells
Plasma cells
d
Examples of PC and activated (tdTomato+) B cells in the lung 20 days post primary infection:
150μm
100μm
200μm
AID(Cre)-tdTomato Collagen PCs (surfaces)
e
BRM cell Ig switched (% of BRM cells)
IgA
IgG
IgM
f
PC cell count
*
No Rechall.
Rechallenge
g
IgA switched PCs (% of PCs)
ns
IgG switched PCs (% of PCs)
***
IgM switched PCs (% of PCs)
ns

Supplementary Fig. 3 Visualization and quantification of PC distribution in influenza infected lungs form BAT mice, related to Fig.3

(a-c) Analysis pipeline for defining infected and uninfected sites and distribution of BRM and PCs within these sites. (a) An example of an unprocessed image of a tiled static lung section collected by TPLSM, 4 days post rechallenge of BAT mice with CFP-S-Flu. Collagen was captured in the second harmonic channel (blue). (b) Spatial point patterns, showing CFP infected cell tracking and positioning (black dots, top). The image was partitioned into sectors, as shown, and the density of CFP^+ cells within each sector was quantified to assign it a value as infected or uninfected (bottom). (c) BRM cells and PCs were identified (red and yellow circles, respectively) and their densities in infected (blue) or uninfected (white) sites was determined. (d) Distribution of PCs during primary infection. Large tiles of static lung sections from BAT mice imaged using TPLSM 20 days post primary infection. At this time point, PCs are detected in the lungs, but they are confined to PC clusters and iBALT-like structures and are not observed within alveoli. Images are representative of 3 mice imaged in 3 independent experiments. (e) Frequencies of IgA, IgM and a combination of IgG1, IgG2b and IgG2a[b] (collectively referred to as 'IgG') isotypes within the BRM cell subset (defined as i.v. $CD45^-$ $B220^+$ $GL7^-$ $tdTomato^+$ $mVenus^-$) in the lung of BAT mice during the memory phase (>6 weeks post primary infection). (f, g) Numbers (f) and Ig isotype frequencies (g) of lung PCs in BAT mice before and 4 days post rechallenge. PCs were defined as i.v. $CD45^-$ $GL7^-$ $tdTomato^+$ $mVenus^+$. Frequencies of IgA, IgM and IgG (including IgG1, IgG2b and IgG2a[b]) within the total PC subset were defined based on intracellular stain with the indicated Ig isotype. Figure shows pooled results of one from 3 independent experiments performed. Each circle represents one mouse.

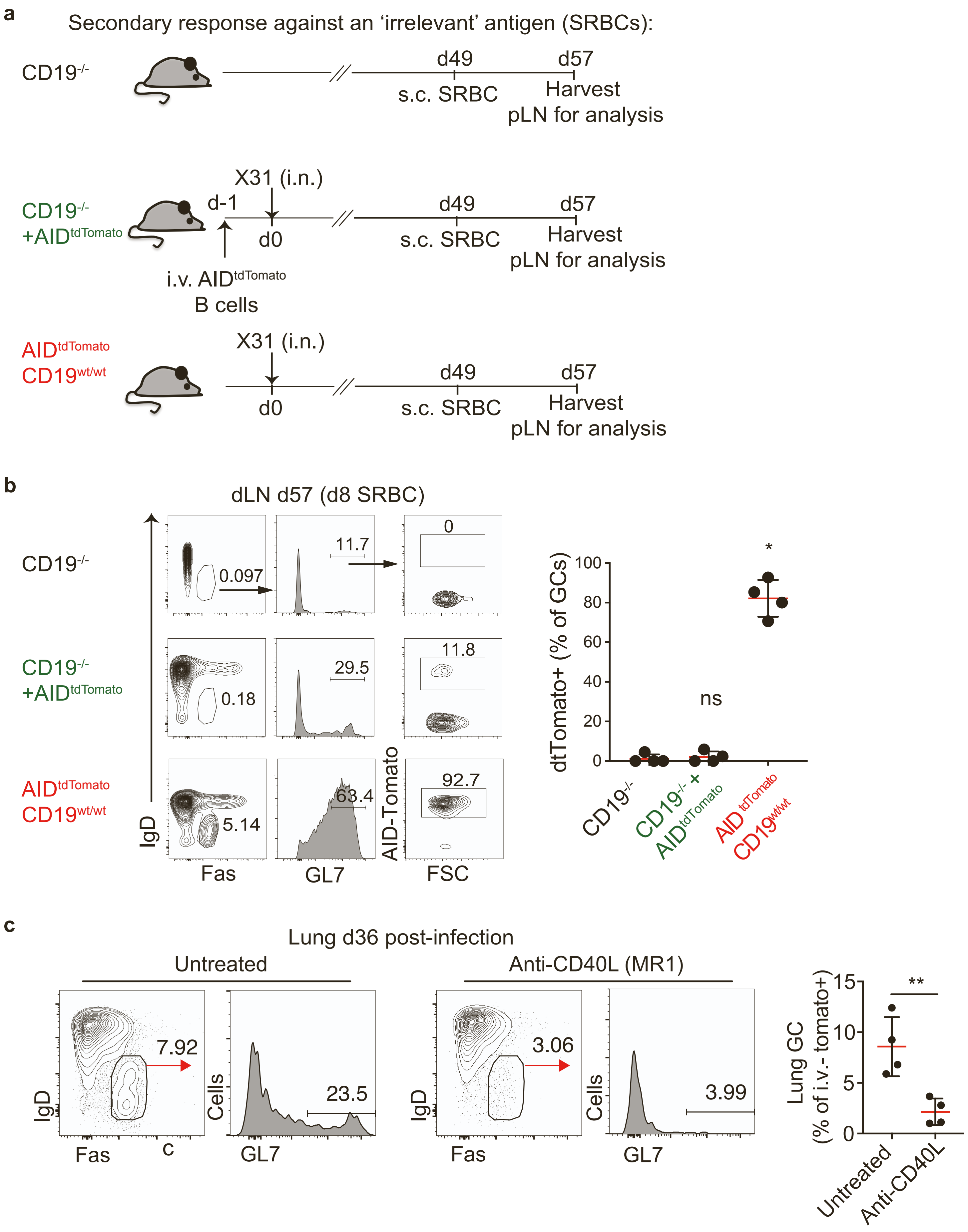

a
Secondary response against an 'irrelevant' antigen (SRBCs):
CD19-/-
d49
d57
s.c. SRBC
Harvest
pLN for analysis
CD19-/-
+AIDtdTomato
X31 (i.n.)
d-1
d0
i.v. AIDtdTomato
B cells
AIDtdTomato
CD19wt/wt
b
dLN d57 (d8 SRBC)
0.097
11.7
0
0.18
29.5
11.8
5.14
63.4
92.7
IgD
Fas
GL7
AID-Tomato
FSC
dtTomato+ (% of GCs)
ns
*
c
Lung d36 post-infection
Untreated
Anti-CD40L (MR1)
7.92
23.5
3.06
3.99
Cells
Lung GC
(% of i.v.- tomato+)
**
Untreated
Anti-CD40L

Supplementary Fig. 4. Validation of approaches to track reactivation of memory B cells in the lung, related to Fig.5

(a-b) Validation of the CD19$^{-/-}$ transfer system as an effective approach to track memory, but not naïve B cell reactivation. Experimental outline. To generate mice in which only transferred labelled cells can generate memory B cell responses, CD19$^{-/-}$ mice were adoptively transferred with CD19^{WT} AID-Cre^{tdTomato} B cells. CD19-/- mice without adoptive transfers were used as negative controls (for mice that cannot generate memory B cells). As a positive control (for mice that are able to generate endogenous memory B cells), we used CD19^{WT} B cells from AID-Cre^{tdTomato} mice. Animals were infected with influenza virus and 45 days later were rechallenged s.c. with SRBCs. 8d later draining LNs were collected for analysis of GC B cells. (b) Left, representative FACS plots and gating strategy to identify tdTomato^{+} cells within GC (defined as IgD^{low} Fas^{+} GL7^{+} tdTomato^{+}). Plots are pre-gated on B220^{+} live cells. Right, quantification of frequencies of tdTomato^{+} cells within GC gates. Figure shows results from one experiment from two performed. Each circle represents one mouse. (c) Validation of anti-CD40L antibody treatment. Mice were infected with X31 influenza and on d26 started treatment with i.v. anti-CD40L antibodies for 10 days as illustrated in Fig. 5e. Mice were in vivo labelled with anti-CD45 4 mins prior to collection of lungs. The frequencies of lung GC B cells were determined by FACS, identified as i.v. CD45^{-} tdTomato^{+} IgD^{low} Fas^{+} GL7^{+} GC B cells, as indicated. Left, shown representative plots, pre-gated on B220^{+} i.v. CD45^{-} live cells. Right, quantification of GC B cell frequencies of i.v. CD45^{-} tdTomato^{+} gate. Figure shows results from one experiment from two performed. Each circle represents one mouse.

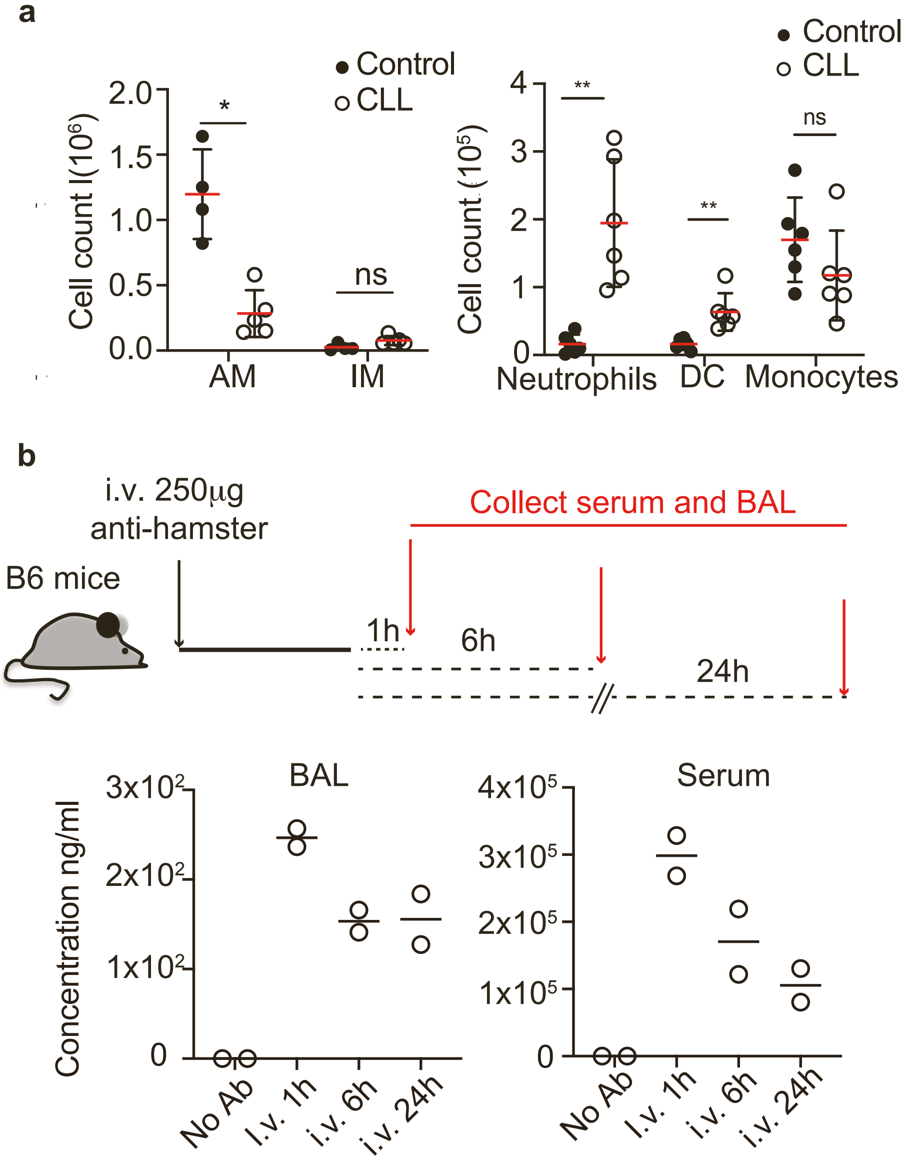

Supplementary Fig.5. Ablation of alveolar macrophages using CLL and kinetics of serum antibody saturation in the BAL, related to Fig.6

(a) Cell counts for alveolar macrophages (AM, i.v. CD45$^-$, ex vivo CD45$^+$ F4/80$^+$ CD11c$^+$ CD11b$^-$ SiglecF$^+$), interstitial macrophages (IM, i.v. CD45$^-$, ex vivo CD45$^+$ F4/80$^+$ CD11c$^+$ CD11b$^+$ SiglecF$^-$), neutrophil (i.v. CD45$^-$, ex vivo CD45$^+$ Ly6G$^+$ CD11b$^+$), dendritic cell (i.v. CD45$^-$, ex vivo CD45$^+$ F4/80$^-$ CD11c^{hi}) and monocyte (i.v. CD45$^-$, ex vivo CD45$^+$ Ly6G$^-$ Ly6C$^+$) in total lungs derived from memory mice treated intranasally with CLL 6 and 3 days prior to tissue collection. Figure shows results of one from three independent experiments performed. (b) Defining the kinetics of i.v. injected antibodies infiltration of the BAL. Top, experimental set up. Mice were injected i.v. with 250μg anti-hamster antibodies. 1, 6 and 24 hours later, blood and BAL were collected. The concentrations of hamster IgG in these compartments were determined by ELISA. Figure shows results of one from two independent experiments performed. Each circle represents one mouse.

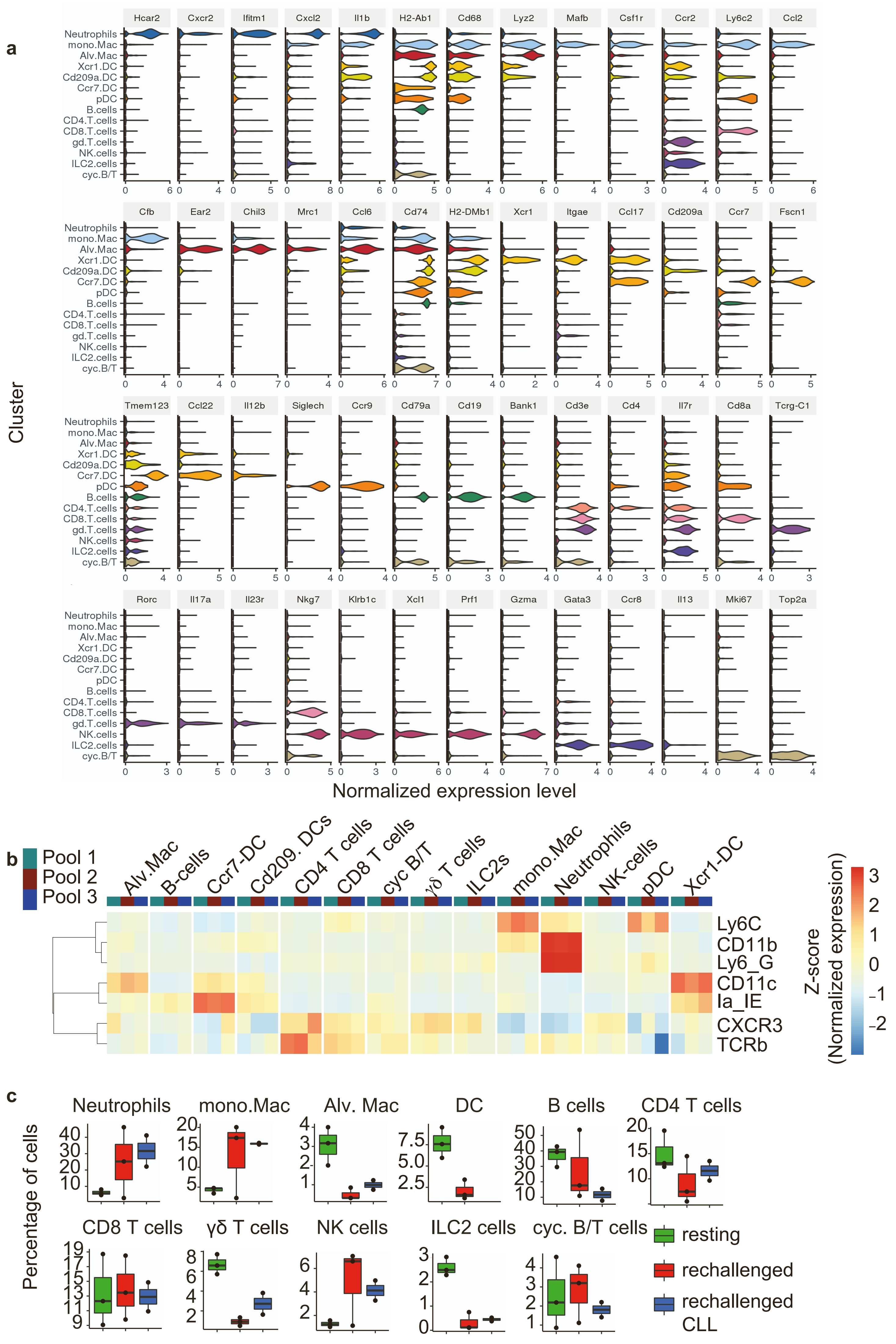
a
Hcar2
Cxcr2
Ifitm1
Cxcl2
Il1b
H2-Ab1
Cd68
Lyz2
Mafb
Csf1r
Ccr2
Ly6c2
Ccl2
Cfb
Ear2
Chil3
Mrc1
Ccl6
Cd74
H2-DMb1
Xcr1
Itgae
Ccl17
Cd209a
Ccr7
Fscn1
Tmem123
Ccl22
Il12b
Siglech
Ccr9
Cd79a
Cd19
Bank1
Cd3e
Cd4
Il7r
Cd8a
Tcrg-C1
Rorc
Il17a
Il23r
Nkg7
Klrb1c
Xcl1
Prf1
Gzma
Gata3
Ccr8
Il13
Mki67
Top2a
Neutrophils
mono.Mac
Alv.Mac
Xcr1.DC
Cd209a.DC
Ccr7.DC
pDC
B.cells
CD4.T.cells
CD8.T.cells
gd.T.cells
NK.cells
ILC2.cells
cyc.B/T
Cluster
Normalized expression level
b
Pool 1
Pool 2
Pool 3
Alv.Mac
B-cells
Ccr7-DC
Cd209. DCs
CD4 T cells
CD8 T cells
cyc B/T
γδ T cells
ILC2s
mono.Mac
Neutrophils
NK-cells
pDC
Xcr1-DC
Ly6C
CD11b
Ly6_G
CD11c
Ia_IE
CXCR3
TCRb
Z-score
(Normalized expression)
c
Neutrophils
mono.Mac
Alv. Mac
DC
B cells
CD4 T cells
CD8 T cells
γδ T cells
NK cells
ILC2 cells
cyc. B/T cells
Percentage of cells
resting
rechallenged
rechallenged CLL

Supplementary Fig.6 Changes in the cellular landscape of rechallenged mice in the presence and absence of alveolar macrophages, related to Fig.7

scRNA-seq analysis of lung leukocytes from mice treated as described in Figs. 7a-c. (a) Violin plots showing marker genes for the cell clusters that are conserved between the three experimental conditions. (b) Heatmap showing the expression of surface proteins by cluster (CITE-seq analysis). (c) Box plots showing the frequencies of the different cell types by experimental group.

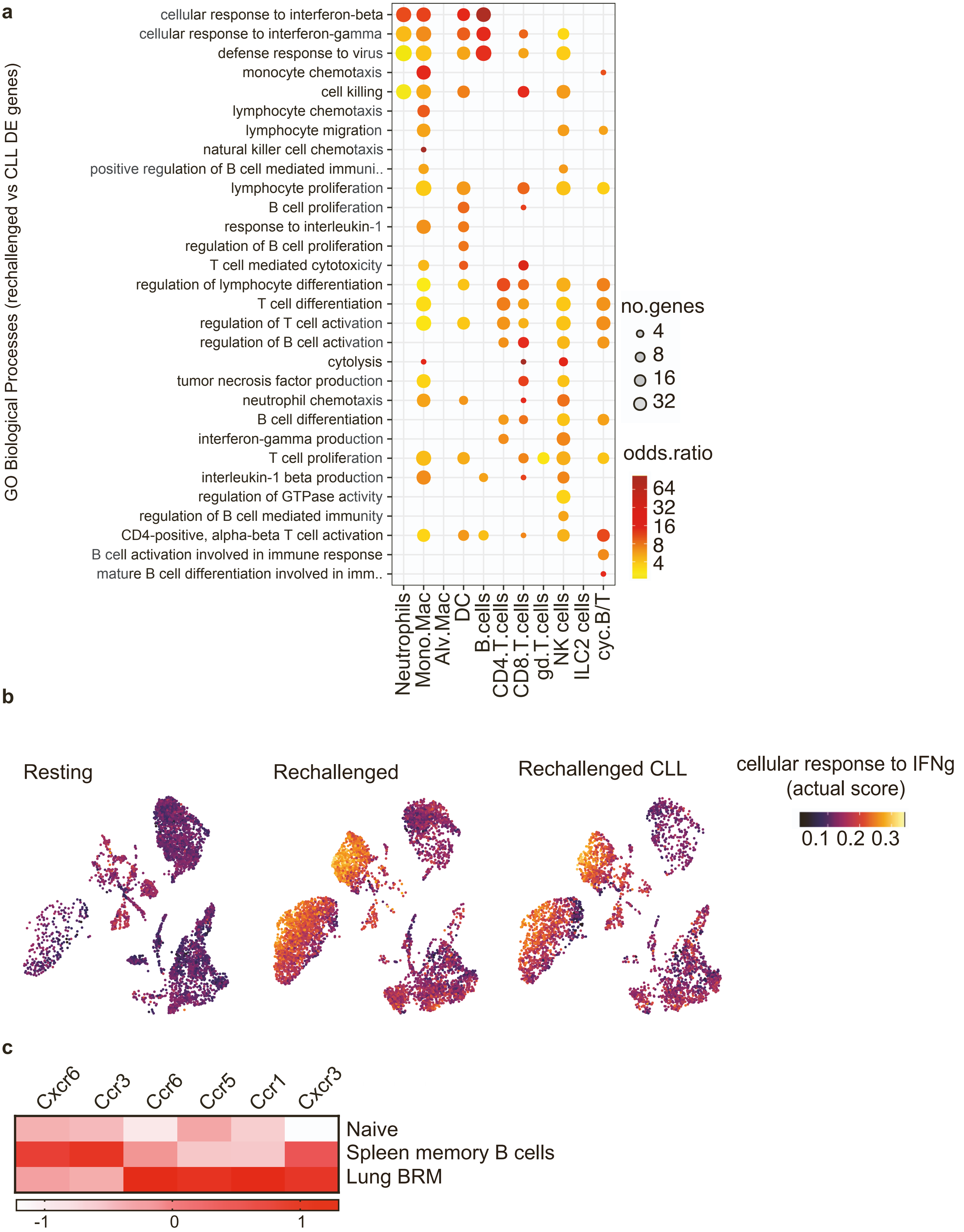
a
GO Biological Processes (rechallenged vs CLL DE genes)
cellular response to interferon-beta
cellular response to interferon-gamma
defense response to virus
monocyte chemotaxis
cell killing
lymphocyte chemotaxis
lymphocyte migration
natural killer cell chemotaxis
positive regulation of B cell mediated immuni..
lymphocyte proliferation
B cell proliferation
response to interleukin-1
regulation of B cell proliferation
T cell mediated cytotoxicity
regulation of lymphocyte differentiation
T cell differentiation
regulation of T cell activation
regulation of B cell activation
cytolysis
tumor necrosis factor production
neutrophil chemotaxis
B cell differentiation
interferon-gamma production
T cell proliferation
interleukin-1 beta production
regulation of GTPase activity
regulation of B cell mediated immunity
CD4-positive, alpha-beta T cell activation
B cell activation involved in immune response
mature B cell differentiation involved in imm..
Neutrophils
Mono.Mac
Alv.Mac
DC
B.cells
CD4.T.cells
CD8.T.cells
gd.T.cells
NK cells
ILC2 cells
cyc.B/T
no.genes
4
8
16
32
odds.ratio
64
32
16
8
4
b
Resting
Rechallenged
Rechallenged CLL
cellular response to IFNg
(actual score)
0.1 0.2 0.3
c
Cxcr6
Ccr3
Ccr6
Ccr5
Ccr1
Cxcr3
Naive
Spleen memory B cells
Lung BRM
-1
0
1

Supplementary Fig.7 Depletion of alveolar macrophages leads to impaired myeloid cell IFNG response, related to Fig.7

scRNA-seq analysis of lung leukocytes from mice treated as described in Figs. 7a-c. (a) Dotplots showing selected Gene Ontology (GO) Biological Processes (BP) that are significantly over-represented in genes differentially expressed between the CLL treated rechallenge and rechallenge conditions within the cell types (One sided Fisher's exact tests; BH adjusted $p < 0.05$). (b) UMAPs showing the expression of the GO BP "cellular response to IFNG" geneset in the cells from each of the three conditions (AUCell analysis). (c) Heatmap showing qPCR expression of the indicated inflammatory chemokine receptors from sorted naïve B cells, splenic memory B cells and lung BRM cells. Data in c represent analysis of the same experiment described in Fig. 1b.
